# Predicting Confined 1D Cell Migration from Parameters Calibrated to a 2D Motor-Clutch Model

**DOI:** 10.1016/j.bpj.2020.01.048

**Published:** 2020-02-25

**Authors:** Louis S. Prahl, Maria R. Stanslaski, Pablo Vargas, Matthieu Piel, David J. Odde

**Affiliations:** 1 Department of Biomedical Engineering, University of Minnesota, Minneapolis, Minnesota; 2 Institut Curie, PSL Research University, CNRS UMR 144 and Institut Pierre-Gilles de Gennes, PSL Research University, Paris, France; 3 INSERM U932 Immunité et Cancer, Institut Curie, PSL Research University, Paris, France; 4 Physical Sciences-Oncology Center, University of Minnesota, Minneapolis, Minnesota

## Abstract

Biological tissues contain micrometer-scale gaps and pores, including those found within extracellular matrix fiber networks, between tightly packed cells, and between blood vessels or nerve bundles and their associated basement membranes. These spaces restrict cell motion to a single-spatial dimension (1D), a feature that is not captured in traditional in vitro cell migration assays performed on flat, unconfined two-dimensional (2D) substrates. Mechanical confinement can variably influence cell migration behaviors, and it is presently unclear whether the mechanisms used for migration in 2D unconfined environments are relevant in 1D confined environments. Here, we assessed whether a cell migration simulator and associated parameters previously measured for cells on 2D unconfined compliant hydrogels could predict 1D confined cell migration in microfluidic channels. We manufactured microfluidic devices with narrow channels (60-*μ*m^2^ rectangular cross-sectional area) and tracked human glioma cells that spontaneously migrated within channels. Cell velocities (v_exp_ = 0.51 ± 0.02 *μ*m min^−1^) were comparable to brain tumor expansion rates measured in the clinic. Using motor-clutch model parameters estimated from cells on unconfined 2D planar hydrogel substrates, simulations predicted similar migration velocities (v_sim_ = 0.37 ± 0.04 *μ*m min^−1^) and also predicted the effects of drugs targeting the motor-clutch system or cytoskeletal assembly. These results are consistent with glioma cells utilizing a motor-clutch system to migrate in confined environments.

## Significance

Cells migrating through dense tissues encounter micrometer-scale pores and track-like structures, which contribute additional forces to the cell that are not present in two-dimensional unconfined environments. It is presently unclear whether established models for cell migration apply to confined environments or whether cells adopt specialized mechanisms of force generation to navigate these environments. Simulated cell migration behaviors using a motor-clutch mechanism are consistent with human glioma cell migration in confined one-dimensional microfluidic channels. Simulations can also predict effects of drugs targeting integrin-mediated adhesion, myosin II motors, or cytoskeletal assembly dynamics. Our results suggest that glioma cells employ similar mechanisms in one-dimensional confined channels as on two-dimensional unconfined surfaces.

## Introduction

Cell migration is involved in numerous physiological functions throughout organismal development and adult life. Aberrant cell migration disperses cancer cells into healthy tissue, which creates significant challenges in treating invasive gliomas (1) and other malignant tumors. The physical mechanism of cell migration involves coordinated dynamics of the actin cytoskeleton and adhesion complexes (2,3). F-actin assembly drives the elongation of cellular protrusions, whereas within protrusions, adhesion receptors (termed “clutches”) assemble into complexes and link cells to extracellular matrix (ECM) ligands. Bound clutches cooperatively transmit contractile actin-myosin forces to the ECM, establishing traction forces that drive locomotion.

Integrated physical models incorporate mathematical expressions for these molecular processes to successfully predict experimentally measured cell migration behaviors (3, 4, 5). Building upon an established motor-clutch model for cell traction (6, 7, 8), a recently developed computational cell migration simulator (CMS) reproduces the characteristic random motility of glioma cells on compliant hydrogels (9,10). Further studies reproduced glioma cell migration behaviors in environments with varying adhesiveness (11) and complex fiber orientation (12,13) by changing relevant environmental parameters. However, these studies did not consider the mechanical confinement encountered by invading glioma cells as they escape the tumor bulk (14) and encounter an ECM composed of hyaluronic acid and densely packed cells (15,16). Cells invading along perivascular spaces and the glia limitans encounter micrometer-sized gaps between these structures and rigid basement membranes composed of collagen IV and laminin (16,17). Confinement is thus an important consideration in developing theoretical models of in vivo cell migration.

In vitro studies reveal varied, and sometimes surprising, cellular responses to confinement, including bleb-based fast amoeboid migration (18, 19, 20) or impaired movement of the nucleus through micrometer-scale pores (21,22). Adding to this complexity, pharmacological agents that inhibit migration on unconfined two-dimensional (2D) substrates are sometimes reported to have little or no effect on cells migrating in confined environments (23, 24, 25). Correspondingly, a number of biophysical theories have been proposed to explain specific behaviors, including adhesion-independent migration by frictional forces (19) and actin-independent protrusive forces provided by osmotic pressure gradients (23,26). These models are largely supported by data obtained in confinement and untested in other contexts.

Traditional cell migration assays are typically performed on glass or polystyrene dishes or hydrogels, which do not confine cells, raising the question of how relevant the mechanisms used in unconfined contexts are to confined environments. ECM proteins deposited into lanes (27), microgrooved silicon wafers (28), and suspended polystyrene fibers (12) replicate the one-dimensional (1D) aligned structures found in these regions (17). Cell contact guidance along these structures notably increases the directional persistence of cells (12,27), but these cells do not experience mechanical confinement as they would in three-dimensional tissue environments. To overcome these limits, photolithography and polydimethylsiloxane (PDMS) replica molding can create confined channels that also permit microscopy-based measurements of intracellular structure and dynamics (21, 22, 23, 24,29, 30, 31, 32, 33, 34).

To test confined cell migration predictions using a motor-clutch model, we created a 1D CMS to recapitulate directional guidance cues found in aligned brain tissue structures and linear microchannel designs. Tracking individual glioma cells in PDMS channels revealed spontaneous and persistent cell migration along the channel axis, which simulations predicted using parameter sets measured for cells on unconfined hydrogels (9,10). By changing relevant parameters, the 1D CMS also successfully predicted migration phenotypes of cells exposed to pharmacological agents targeting various motor-clutch components or cytoskeletal assembly dynamics. These combined results are consistent with glioma cells employing a motor-clutch mechanism to migrate in confined microfluidic channels.

## Materials and Methods

### 1D CMS

The 1D CMS employed in this study is modified from a previously described 2D CMS (9, 10, 11, 12). A detailed model description can be found in the Supporting Materials and Methods. Simulation parameters and their estimated or measured values are reported in Table S1. Simulations were coded and run in MATLAB (The MathWorks, Natick, MA). Cell body position (x_cell_) was recorded from simulations, and mean-squared displacement (MSD) was computed from x_cell_ using the overlap method (35).

### Microchannel devices

Microchannel devices were based on previous designs (36) and were drawn using a computer-aided design software (AutoCAD; Autodesk, San Rafael, CA). Quartz-chrome photomasks containing the device patterns were produced from these designs using Minnesota Nano Center facilities and were used to create master molds for device designs on silicon wafers using standard photolithography techniques. Photolithography, PDMS replica molding, and device assembly are described in detail in the Supporting Materials and Methods.

### Cell culture and imaging

Human U251 glioma cells were cultured at 5% CO_2_ and 37°C. Culture media consisted of Dulbecco’s modified eagle media/F12 (Gibco, Thermo Fisher Scientific, Waltham, MA) supplemented with 10% (v/v) fetal bovine serum (Gibco), 100 U mL^−1^ penicillin (Corning, Corning, NY), and 100 *μ*g mL^−1^ streptomycin (Corning). Cells were passaged and subcultured using 0.25% trypsin-EDTA (Corning). Fluorescent proteins (eGFP-*β*-actin or end-binding protein 1 (EB1)-eGFP) were transiently transfected into cells as described previously (10). Nuclei were labeled using NucBlue Live ReadyProbes reagent (Thermo Fisher Scientific) before seeding cells in device inlets. Images of migrating cells were acquired every 5 min at 20× magnification on a Nikon Eclipse Ti2 or Ti-E microscope (Nikon Instruments, Melville, NY) under control of NIS-Elements Advanced Research software (Nikon). Individual cell nuclei positions were measured at each frame using a custom analysis script (11). MSDs were computed from experimental trajectories in the same way as simulations. Full details on experimental procedures and data analysis are described in the Supporting Materials and Methods.

### Statistical analysis

Numbers of measurements are given in figure legends. Kruskal-Wallis one-way analysis of variance (ANOVA) was used unless otherwise noted.

## Results

### Switch between random and persistent simulated migration in 1D is a function of asymmetric protrusion nucleation probabilities

**Figure 1:**
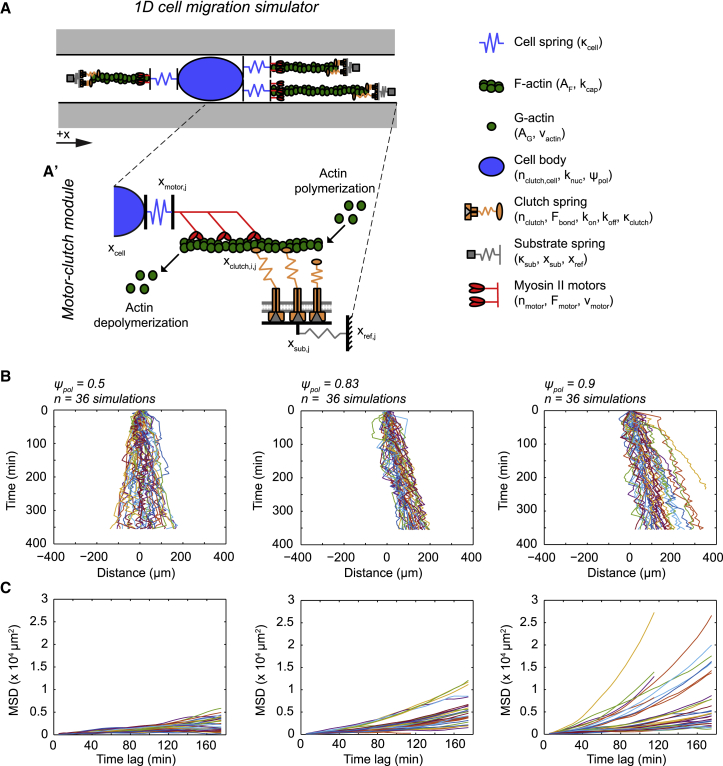
Description and migration dynamics of a 1D CMS. (*A*) A schematic of a 1D CMS within a confined channel whose axis is denoted by the *x* axis is given; gray boxes denote channel walls. Modules containing myosin II motors (n_motor_) and adhesion clutches (n_clutch_) attach to a central cell body through compliant springs. F-actin retrograde flow by myosin II motors and adhesion clutches are governed by similar rules to those described for previous iterations of the motor-clutch model (6,40). Cell body clutches (not pictured) associate with the cell center x_cell_ and undergo binding and unbinding as module clutches but are not subject to direct forces by F-actin retrograde flow. Each module contains an F-actin bundle (A_F,j_ for the length of the j^th^ module bundle) to which clutches bind. The total available G-actin in the cell (A_G_) constrains module nucleation (with base rate constant k_nuc,0_, governed by Eq. S8) and scales actin polymerization speed at the end of modules (maximal speed is v_actin,max_, governed by Eq. S3). Module capping (k_cap_) terminates polymerization and facilitates module shortening and turnover, whereas *ψ*_pol_ gives the probability of new protrusions being generated in the +*x* direction. The number of modules nucleated by a given cell is not constrained, and multiple overlapping modules at the leading or trailing edge of the cell is permitted and denoted by cell springs (*κ*_cell_) drawn in parallel. (*A*′) The inset shows a schematic of a single module (i.e., the j^th^ module) within the simulation. Within the j^th^ module, the distal end of the substrate spring is at a reference point x_ref,j_, whereas the other end serves as the anchoring point for the clutch ensemble at x_sub,j._ The ensemble of n_clutch,j_ clutches within the j^th^ module attaches to the F-actin filament, and x_clutch,j_ represents the average location of the extended clutch springs. Actin polymerizes at the distal end of modules and depolymerizes when it passes the motor ensemble, located at x_motor,j_. Movement of the cell body (x_cell_, pictured as the center of the nucleus) is governed by force balances on each module and the cell body clutches (see, Eqs. S5–S7). (*B*) The simulation position is shown as a function of time for individual 1D CMS runs in which *ψ*_pol_ = 0.5–0.9 (*n* = 36 simulated trajectories for each condition). The initial position is marked at x(*τ* = 0) = 0. (*C*) The MSD versus time lag is shown for the 1D CMS trajectories in (*B*). All simulations were run with n_motor_ = 1000 and n_clutch_ = 750; all other parameter values reported in Table S1.

In the previously described CMS, a force balance between stochastically nucleated cell protrusion modules and the cell body drives random motility in 2D substrates (9). For this study, we modified the CMS to solve for cell coordinates within a single-spatial dimension (1D CMS; Fig. 1 *A*), and the model and underlying equations are fully described in the Supporting Materials and Methods. Each simulated cell contains an ensemble of n_motor_ myosin II motors, n_clutch_ molecular adhesion clutches, and a total pool of F-actin (A_total_) that form the basis for protrusion modules. Individual motors each produce a stall force of F_motor_. The motor ensemble drives retrograde flow of F-actin bundles within modules at a maximal unloaded velocity (v_motor_), which decreases under the load by a linear force-velocity relationship (6), as described in Eq. S2. We note that actin polymerization and myosin II motors both obey a monotonic force-velocity relationship (3), and both produce similar stall forces at the level of individual motor proteins and filaments. This means that both are capable of providing the force driving F-actin retrograde flow in the motor-clutch model, as previously suggested (37,38). Clutches are modeled as elastic springs (with stiffness *κ*_clutch_) that can bind and unbind F-actin at rates k_on_ and k_off_, respectively. k_off_ follows a force-dependent rate law described in Eq. S1 and (6) that also depends on the characteristic bond rupture force F_bond_. Bound clutches transmit forces to the substrate, which is modeled as a linear spring with stiffness *κ*_sub_. The value of *κ*_sub_ was set to 1000 pN nm^−1^ to reflect the rigid modulus of elastomer materials (elastic modulus, E = ∼1000 kPa) used to make microchannel devices (39).

Linear springs representing the nucleo-cytoskeletal compliance (*κ*_cell_) connect modules to the central cell body, which contains an ensemble of clutches (n_clutch,cell_) that follow the same binding and unbinding rules as module clutches. F-actin assembly fuels the module elongation velocity (v_actin_), distributing A_total_ between modules and a soluble pool (A_G_). New modules are nucleated at a rate (k_nuc_) that scales with A_G_ by a power-law relationship (9), described in Eq. S8. Modules are capped at a first-order capping rate (k_cap_). Capped modules no longer extend by actin polymerization but still shorten by retrograde flow until they are destroyed after passing a minimal threshold length (L_min_). Mass balances on n_motor_, n_clutch_, and A_total_ govern their distribution between modules and the cell body. In total, the 1D CMS contains 18 parameters for the cell, eight of which define the motor-clutch system properties (40), plus a variable substrate stiffness (Table S1). The values of these parameters and the constraints on their relationships have been previously described by both our previous work and that of several other labs (6,7,9,40, 41, 42, 43, 44).

In the previous 2D CMS (9), new modules were generated at a random angle in the Cartesian *x*, *y* plane (i.e., between 0 and 2*π* radians). Initially, the 1D CMS assigned modules a random binary orientation along the ±*x* direction (i.e., 0 or *π* radians) with equal probability of nucleating new modules in either orientation. Multiple modules overlapping in one direction is permitted because cells can extend multiple modules in a similar vector direction, such as along parallel-aligned fibers (12). Simulated trajectories obtained from sampling the cell body position (x_cell_) at 5 min intervals (Fig. 1 *B*) yielded approximately linear MSD versus time curves (Fig. 1 *C*), consistent with a 1D random walk (35). This is expected given that earlier versions of the CMS predict a 2D random walk (9). By contrast, previous studies of cell migration in confined microfluidic channels suggest that cells often follow persistent ballistic trajectories (29,32).

To test whether the 1D CMS could produce ballistic trajectories, we added a variable polarity factor *ψ*_pol_ to simulations. As in previous versions of the CMS, module nucleation is a possible event at each simulation step that occurs at a G-actin-dependent rate k_nuc_ (see Eq. S8). The value of *ψ*_pol_ is defined between 0 and 1 and represents the probability that a newly nucleated module will be oriented in the +*x* direction. The corresponding probability (1 − *ψ*_pol_) thus gives the probability that a module is nucleated in the −*x* direction. In other words, the probability that a new module will be nucleated pointing in the +*x* direction follows a binomial distribution with parameters of *ψ*_pol_ and ±*x* as the possible outcomes (Fig. 1 *B*). Starting from the case in which nucleation probability is uniform (*ψ*_pol_ = 0.5), increasing *ψ*_pol_ consistently produced drift in the +*x* direction for individual cell traces (Fig. 1 *B*), which yielded nonlinear (concave up) MSD versus time lag plots (Fig. 1 *C*). This behavior is consistent with previously described superdiffusive models of cell motility, including the persistent random walk (PRW) model that is often used to analyze directed cell migration (45,46). Intriguingly, changing the value of *ψ*_pol_ did not change the average number of protrusions (Fig. S1), confirming that persistent migration can be recapitulated in the 1D CMS as a function of directionally biased module nucleation and turnover.

### Human glioma cells move persistently in confined microchannels

**Figure 2:**
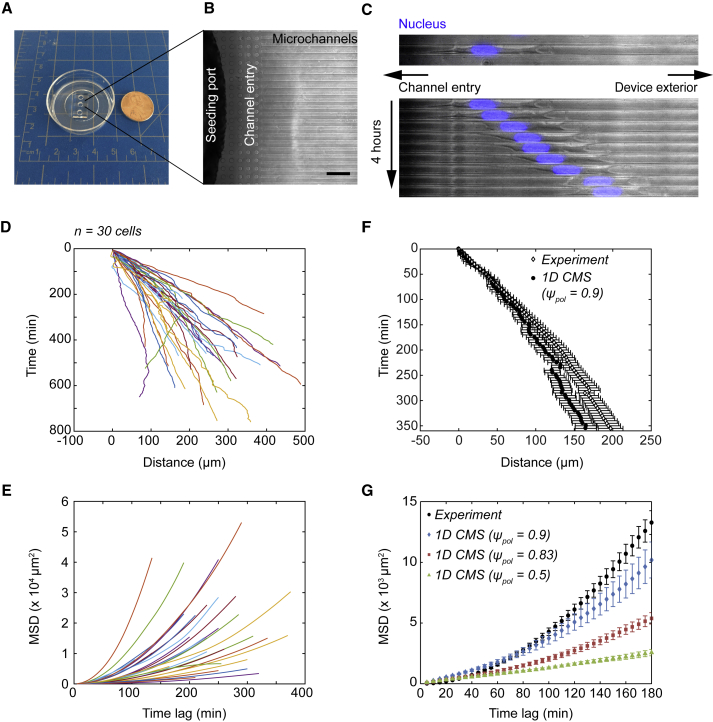
Tracking individual glioma cell nuclei in channels reveals persistent migration behaviors. (*A*) A photograph of an assembled device bonded to a 35-mm glass-bottom dish is shown. Note the channels extending from inlet seeding ports drilled into the PDMS block. A US one-cent coin is shown for scale, and the grid spacing is 1 cm. (*B*) An image of a drilled inlet port in an assembled device showing the entry chamber and 12-*μ*m-wide channels (a height of 5 *μ*m), acquired at 10× magnification using phase contrast optics, is given. Scale bars, 100 *μ*m. (*C*) (*Top*) U251 human glioma cell migrating within a confined 12-*μ*m-wide channel imaged using phase contrast and fluorescence is shown. Nucleus counterstain is shown in blue. Images were acquired at 20× magnification. Scale bars, 50 *μ*m. (*Bottom*) A time-lapse sequence of images acquired for the cell in (*B*) is given. The images represent 4 h of total time displayed at 30-min intervals. (*D*) The nucleus *x*-position versus time as measured for *n* = 30 individual cells from a representative experiment is shown. Coordinates are plotted relative to the initial tracking position for each cell such that x(*τ* = 0) = 0. For display purposes, coordinates of cells moving in the −x direction (*right* to *left*) were reversed. (*E*) The mean MSD versus time lag for the individual cells in (*E*) is shown. For display purposes, error bars are not shown. (*F*) The mean displacement versus time for a representative experiment (*filled circles*, mean ± SEM in (*D*)) and 1D CMS (*open diamonds*, mean ± SEM for *n* = 36 simulations with *ψ*_pol_ = 0.9 in Fig. 1*B*) is shown. (*G*) The mean MSD versus time lag for a pooled control data set (*black circles*, *n* = 403 cells from 12 independent experiments) and 1D CMS with *ψ*_pol_ = 0.9 (*blue diamonds*, *n* = 60 simulations), *ψ*_pol_ = 0.83 (*red squares*, *n* = 36 simulations), or *ψ*_pol_ = 0.5 (*green triangles*, *n* = 40 simulations) is shown. The error bars are mean ± SEM.

We next sought an engineered platform that would enable us to observe and track individual cells migrating within confined 1D channels and compare to simulation predictions. Using photolithography and PDMS replica-molding techniques (36), we created devices that featured 12-*μ*m-wide channels with a height of 5 *μ*m (60-*μ*m^2^ rectangular cross-sectional area) emerging from inlet ports (Fig. 2, *A* and *B*). U251 cells were labeled with a live-cell nucleus-tracking dye before seeding in devices. Seeded cells spontaneously migrated out of the inlet ports and migrated along the channel axis (Fig. 2 *C*). Time-lapse videos were acquired in the phase and DAPI channels every 5 min, and individual nuclei were tracked using a semiautomated script (11). Migrating cells elongated along the channel axis, generally forming long leading protrusions and smaller trailing ones (Fig. 2 *C*; Video S1), and moved away from the inlets toward the device exterior. The nucleus typically filled the lateral width of the channel and maintained a near-constant size and shape (Fig. S2).

Video S1. U251 Glioma Cells with Fluorescently Labeled Nuclei Migrating in Microchannel Devices Time-lapse images were collected every 5 minutes at 20x magnification with 2x2 pixel binning (645 nm spatial sampling). Images were acquired in both the transmitted channel using phase contrast optics and LED fluorescence excitation (395 nm) using a DAPI/FITC/TxRed filter set. Scale bar, 50 μm.

Individual cell trajectories exhibited variability (*n* = 30 cells from an example experiment in Fig. 2 *D*), but most cells moved persistently in one direction (away from the inlet port). Few cells exhibited saltatory motion as observed for glioma cells migrating in ex vivo brain slice cultures (11,14), and complete directional reversals were rare. Individual MSD versus time lag plots for experiments (Fig. 2 *E*) were typically nonlinear (concave up), consistent with superdiffusive or quasiballistic cell migration models (12,45,46). Mean displacement and MSD were also consistent with the 1D CMS predictions (*ψ*_pol_ = 0.9; Fig. 2, *F* and *G*). Similar simulated cell behaviors were observed when the substrate spring constant was increased to *κ*_sub_ = 10^6^ pN nm^−1^ (Fig. S3), suggesting that the 1D CMS predicts a similar behavior for cells adhering to the PDMS device walls and glass bottom.

### A diffusion-convection model describes 1D confined cell migration, whereas a PRW model yields unrealistic fitting parameters

Directed cell migration behaviors are often analyzed using a PRW model (12,46). The PRW model relates MSD $\left(\langle r^{2}\left(t\right)\rangle \right)$ for a given time lag (t) to two fitting parameters: cell speed (S) and characteristic persistence time (P).

(1) $$\langle r^{2}\left(t\right)\rangle =nS^{2}P^{2}\left(e^{\frac{-t}{P}}+\frac{t}{P}-1\right).$$

Fitting a pooled control data set (*n* = 403 cells from 12 independent experiments) to Eq. 1, we obtained a mean speed (S = 0.74 ± 0.05 *μ*m min^−1^; Fig. S4) that was comparable to other tumor cell lines (25,31,33) and stem cells (24) but much slower than immune cells (22,29,36) in channels of similar size. Mean persistence times measured from individual cell fits to Eq. 1 (1258 min; Fig. S4) exceeded the maximal imaging window duration (t_exp_ = 18 h = 1080 min). In contrast, glioma cells migrating along suspended polystyrene fibers have persistence times in the range of ∼100 min, well within the bounds of typical microscopy experiments (12). Caution should thus be exercised in interpreting results obtained with a PRW model to avoid overfitting parameters.

Diffusion-convection or diffusion-drift models are also applied to study molecular-cellular scale motion, such as biopolymer filament assembly (47,48). Equation 2 relates MSD to a linear term (*μ*) that is analogous to a diffusion or motility coefficient and a quadratic term (v) representing drift velocity.

(2) $$\langle r^{2}\left(t\right)\rangle =2n\mu t+v^{2}t^{2}.$$

**Figure 3:**
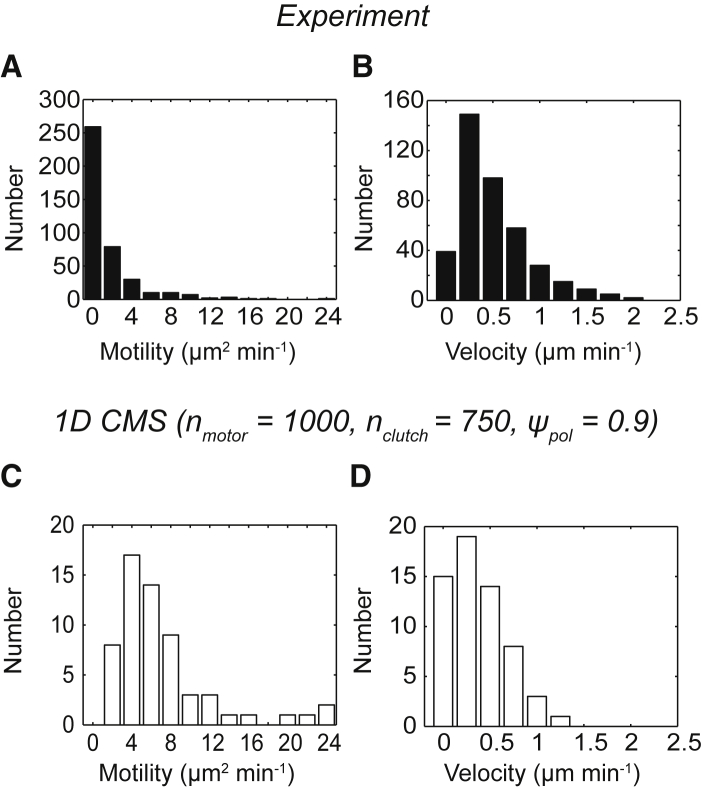
Estimates of motility coefficient and velocity from experimental and simulated data. (*A*) The histogram of the motility coefficients obtained for *n* = 403 cells, *μ*_exp_ = 1.61 ± 0.14 *μ*m^2^ min^−1^ (mean ± standard error (SE)), is shown. (*B*) The histogram of the velocities obtained for the cells in (*A*), v_exp_ = 0.51 ± 0.02 *μ*m min^−1^ (mean ± SE), is shown. (*C*) The histogram of the motility coefficients obtained for *n* = 60 individual simulated trajectories with *ψ*_pol_ = 0.9, *μ*_sim_ = 7.27 ± 0.80 *μ*m^2^ min^−1^ (mean ± SE) is shown. (*D*) The histogram of the velocities obtained for *n* = 60 individual simulated trajectories with *ψ*_pol_ = 0.9, v_sim_ = 0.37 ± 0.04 *μ*m min^−1^ (mean ± SE) is shown. Individual motility coefficients and velocities were obtained from fits to Eq. 2. All simulations were run with n_motor_ = 1000, n_clutch_ = 750, and *ψ*_pol_ = 0.9, and all other parameter values reported in Table S1.

Fitting individual cell trajectories, Eq. 2 revealed cell-to-cell variability in both the motility coefficient (Fig. 3 *A*) and velocity (Fig. 3 *B*). A mean velocity of v_exp_ = 0.51 ± 0.02 *μ*m min^−1^ (or v_exp_ = 8.5 ± 0.3 nm s^−1^) agrees with the speeds obtained using the PRW model (Fig. S4).

Analyzing CMS results using Eq. 2 revealed very little difference in the motility coefficient between the three values of *ψ*_pol_, whereas the velocity increased for larger values of *ψ*_pol_ (Fig. S1), corresponding to increasingly persistent migration. Comparing the most similar conditions, the overall mean motility coefficient was larger for simulations (*ψ*_pol_ = 0.9) than experiments (*μ*_exp_ = 1.61 ± 0.14 *μ*m^2^ min^−1^ vs. *μ*_sim_ = 7.27 ± 0.80 *μ*m^2^ min^−1^). This discrepancy is likely due to the short-timescale reversals in cell position that were only seen in simulations (Fig. 1 *B*) because the diffusion term would be more sensitive to noise at short time lags. Simulated cell velocities were similar to experiments (v_exp_ = 0.51 ± 0.02 *μ*m min^−1^ vs. v_sim_ = 0.37 ± 0.04 *μ*m min^−1^), explaining the consistency between the experiment and simulation (Fig. 2 *G*), because the quadratic velocity term dominates the behavior of Eq. 2 for longer time lags.

### CMS predicts the effects of integrin-mediated adhesion and myosin II inhibition on confined glioma cell migration

**Figure 4:**
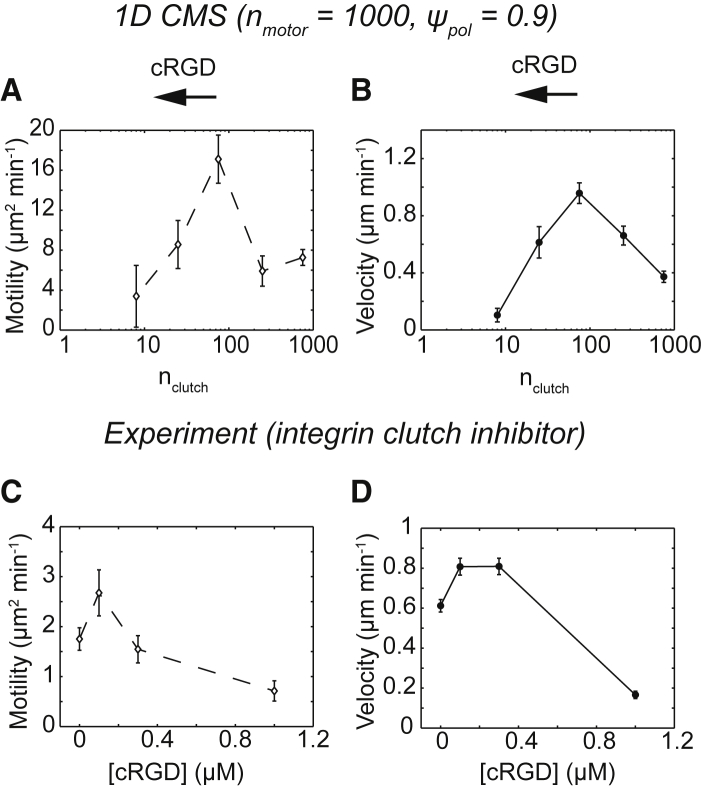
Biphasic relationship between velocity and integrin clutch number for simulations and confined glioma cells. (*A*) The motility coefficients from simulations in which n_clutch_ was varied independently of other parameters are shown (n_clutch_ = 8, 25, 75, 250, and 750 and *n* = 8, 8, 16, 16, and 60 simulated cells). All simulations were run with n_motor_ = 1000 and *ψ*_pol_ = 0.9, and all other parameter values were reported in Table S1. (*B*) The velocities from the simulation conditions in (*A*) are shown. (*C*) The motility coefficients for U251 glioma cells treated with complete media (control) or 0.1, 0.3, or 1 *μ*M cRGD (n ≥ 72 cells) are given. (*D*) The velocities from the experimental conditions in (*C*) are shown. Individual motility coefficients and velocities were obtained from fits to Eq. 2. The error bars represent mean ± SEM. Pairwise statistics are reported in Table S2.

Motor-clutch-based cell migration on 2D hydrogel substrates involves integrin clutches and myosin II motor activity (9), but it is unclear what roles these components play in confined environments. In particular, reducing adhesiveness (achieved by either reducing receptor expression or ligand availability) is theoretically predicted to reduce motility (4,11) but is experimentally shown to increase motility in vitro for some cell types in confinement (18). Earlier studies using the CMS recapitulate a biphasic relationship between adhesiveness and motility (11) in which cells achieve maximal motility at intermediate adhesiveness. We therefore sought to test whether the 1D CMS would produce similar results by reducing the value of n_clutch_. Starting with the base parameter set (n_motor_ = 1000, n_clutch_ = 750, *ψ*_pol_ = 0.9; Table S1), independently reducing n_clutch_ produced a biphasic trend in both the motility coefficient (Fig. 4 *A*) and the velocity (Fig. 4 *B*). Consistent with 2D simulations, the largest value of either quantity was obtained at n_clutch_ = 75 or a 10-fold reduction from the base parameter value (Table S1).

As an experimental test of the model, we treated U251 cells in microchannels with cyclo-(Arg-Gly-Asp) peptide (cRGD), a competitive inhibitor of *α*_v_*β*_3_ integrin-fibronectin interactions (49). On 2D hydrogel substrates, cRGD reduces traction force and migration, consistent with 2D CMS predictions (9). Cells exposed to 0.1–1 *μ*M cRGD demonstrated biphasic trends in the motility coefficient (Fig. 4 *C*) and velocity (Fig. 4 *D*), with the largest values observed at 0.1–0.3 *μ*M. U251 cells thus rely on integrin clutches in confined microchannels, and their migration behavior is consistent with a biphasic adhesiveness relationship (4,11).

**Figure 5:**
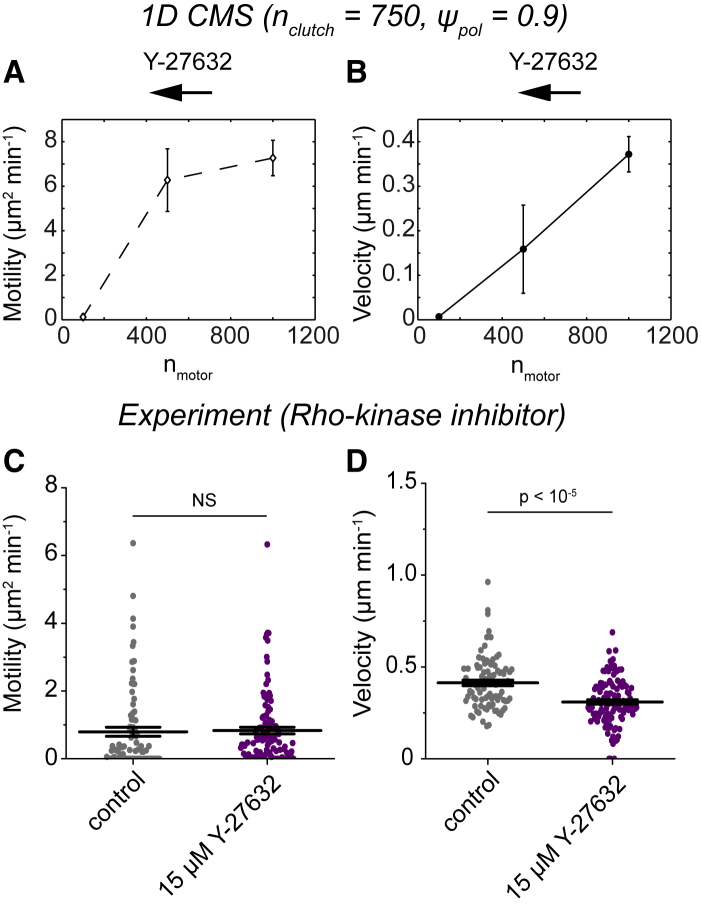
Monotonic relationship between velocity and myosin II motor number for simulations and confined glioma cells. (*A*) The motility coefficients from simulations in which n_motor_ was varied independently of other parameters are shown (n_motor_ = 100, 500, and 1000 and *n* = 8, 8, and 60 simulated cells). All simulations were run with n_clutch_ = 750 and *ψ*_pol_ = 0.9, and all other parameter values were reported in Table S1. (*B*) The velocities from the simulation conditions in (*A*) are shown. (*C*) The motility coefficients for U251 glioma cells treated with complete media (control) or 15 *μ*M Y-27632 (*n* = 87, 112 cells) are given.(*D*) The velocities from the experimental conditions in (*C*) are shown. Individual motility coefficients and velocities were obtained from fits to Eq. 2. The error bars represent mean ± SEM, NS denotes no significant difference, and *p* > 0.01 by one-way Kruskal-Wallis ANOVA. Pairwise statistics for (*A* and *B*) are reported in Table S2. To see this figure in color, go online.

Myosin II inhibition slows glioma cell migration on 2D hydrogels (9) and stalls cell migration in an ex vivo brain slice culture (14,50). Independently reducing simulated myosin II motor number (n_motor_) from its base value (n_motor_ = 1000; Table S1) nearly monotonically reduced the motility coefficient (Fig. 5 *A*) and velocity (Fig. 5 *B*). Our imaging approaches are not compatible with the myosin II inhibitor blebbistatin because imaging at near-ultraviolet wavelengths causes drug inactivation and cytotoxicity (51). Rho-associated kinase (ROCK) is one of the major pathways for myosin II activation in U251 cells (14), so as an orthogonal approach, we inhibited this pathway using 15 *μ*M Y-27632. Compared to controls, Y-27632 had little effect on the motility coefficient (Fig. 5 *C*) but significantly reduced the velocity (Fig. 5 *D*). This reduction in velocity can be explained by reducing n_motor_ in the 1D CMS, suggesting that U251 cells use ROCK-mediated myosin II force generation in confinement. In other studies, confined cells were insensitive to ROCK inhibitors (24,25,52), suggesting either 1) myosin II activation is controlled independently of the ROCK pathway in these cells or 2) other force generation mechanisms such as actin polymerization or osmotic pressure gradients (23) are the dominant means of force generation.

### Actin polymerization and dynamic microtubules are required for confined glioma migration

**Figure 6:**
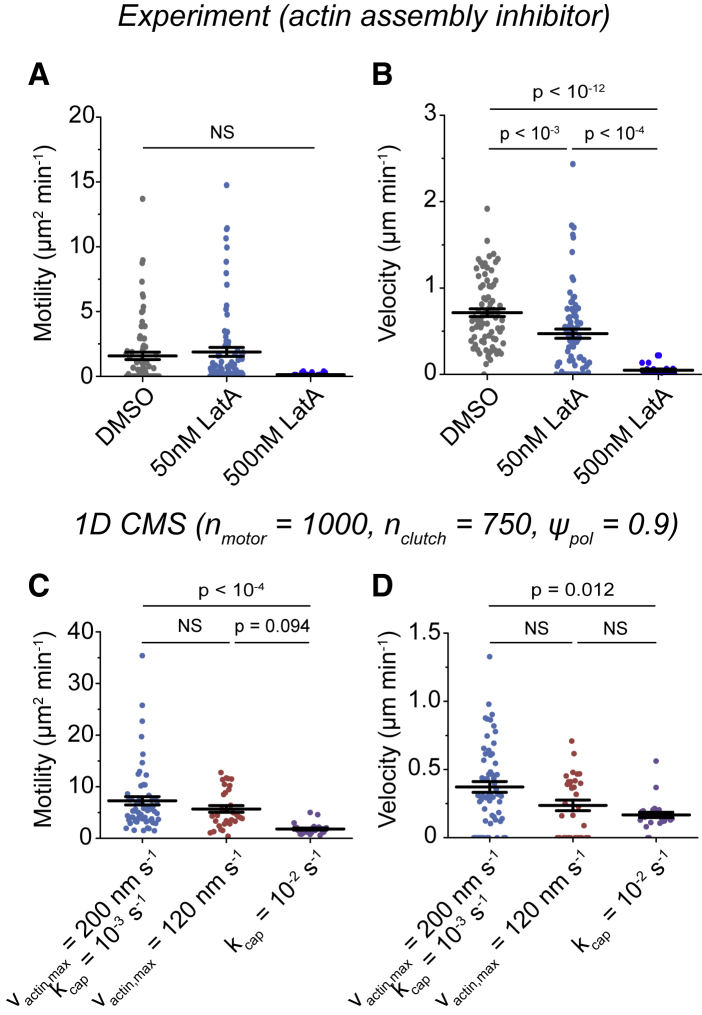
Simulated and experimental predictions of the effects of actin polymerization inhibitors on confined glioma cell migration. (*A*) The motility coefficients for U251 cells expressing eGFP-*β*-actin and treated with vehicle control (DMSO) or 50 or 500 nM LatA are shown (*n* = 78, 81, 23 cells). (*B*) The velocities for the experimental conditions in (*A*) are shown. (*C*) The motility coefficients from simulations using a reference parameter set (Table S1; v_actin,max_ = 200 nm s^−1^, k_cap_ = 0.001 s^−1^) or simulations in which v_actin,max_ was reduced (v_actin,max_ = 120 nm s^−1^) or in which k_cap_ was increased (k_cap_ = 0.01 s^−1^) are shown (*n* = 60, 28, and 24 simulations). All simulations had n_motor_ = 1000, n_clutch_ = 750, and *ψ*_pol_ = 0.9, and all other parameter values are reported in Table S1. (*D*) The velocities from the simulations in (*C*) are shown. Individual motility coefficients and velocities were obtained from fits to Eq. 2. The error bars represent mean ± SEM, NS denotes no significant difference, and *p* > 0.01 by one-way Kruskal-Wallis ANOVA. To see this figure in color, go online.

Actin polymerization is critical for cell migration on unconfined 2D substrates, but its role in confined migration is controversial (23, 24, 25). Notably, confined migration driven by osmotic pressures is insensitive to latrunculin A (LatA), a potent inhibitor of actin polymerization (23). We tested the actin-dependence of confined U251 cell migration by treating cells with either vehicle controls (dimethyl sulfoxide (DMSO)) or varying doses of LatA. Treating cells with 50 nM LatA slowed movement, whereas 500 nM LatA nearly completely stalled motion (Video S2). Tracking cells and quantifying results using Eq. 2 revealed corresponding dose-dependent decreases in the motility coefficient (Fig. 6 *A*) and velocity (Fig. 6 *B*).

Video S2. U251 Glioma Cells Expressing EGFP-Actin and Treated with Vehicle Control or LatA Migrating in Microchannel Devices Time-lapse images were collected every 5 minutes at 20x magnification with 2x2 pixel binning (645 nm spatial sampling). Images were acquired in both the transmitted channel using phase contrast optics and using LED fluorescence excitation (395 nm and 470 nm) through a DAPI/FITC/TxRed filter set. Conditions include DMSO vehicle (top), 50 nM latrunculin A (middle), and 500 nM latrunculin A (bottom). Scale bar, 50 μm.

Actin polymerization drives protrusion extension in the 1D CMS and scales a maximal polymerization rate from its base value (v_actin,max_ = 200 nm s^−1^; Table S1). Reducing the maximum actin polymerization rate (v_actin,max_ = 120 nm s^−1^) impairs motility on 2D substrates (10), and the same parameter value change in the 1D CMS also reduced the cell motility coefficient (Fig. 6 *C*) and velocity (Fig. 6 *D*). As an alternative mechanism, the 1D CMS features a stochastic capping rate for modules (k_cap_ = 10^−3^ s^−1^; Table S1) as a mechanism to facilitate module turnover. Because LatA prevents actin subunits from binding to F-actin barbed ends (53), we tested the possibility that increased module capping would produce similar results. Increasing k_cap_ by an order of magnitude (k_cap_ = 10^−2^ s^−1^) reduced the simulated motility coefficient (Fig. 6 *C*) and velocity (Fig. 6 *D*), similar to decreasing v_actin,max_ but contrasting with our earlier results on 2D substrates (10). Actin protrusion and turnover rates thus regulate migration speed in the 1D CMS, consistent with experimental measurements of cells treated with LatA.

**Figure 7:**
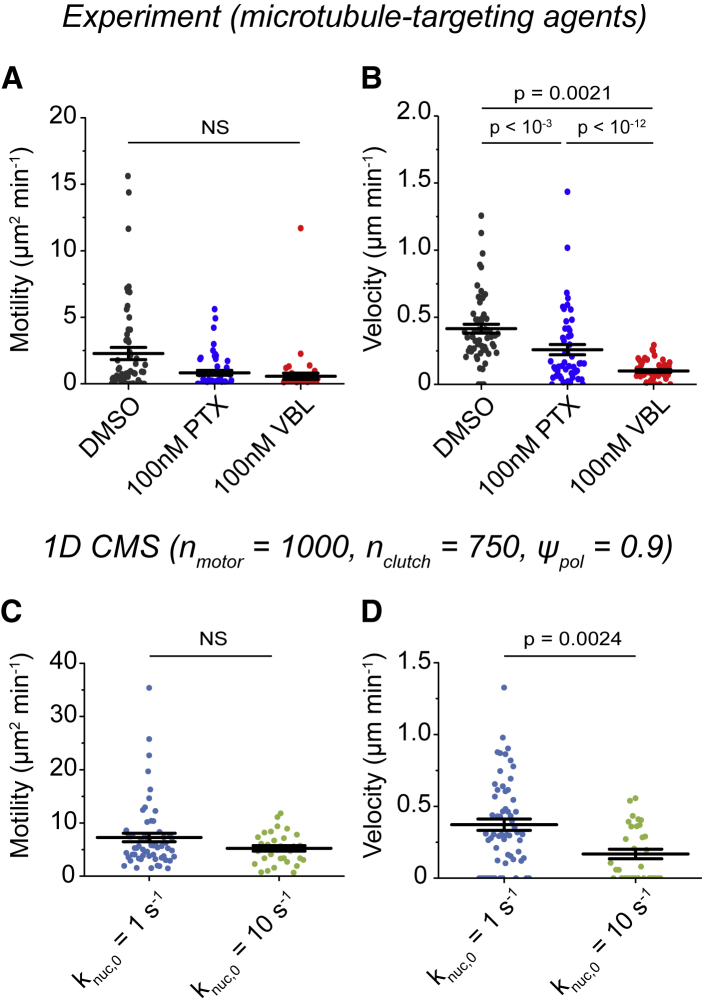
Simulated and experimental predictions of the effects of MTAs on confined glioma cell migration. (*A*) The motility coefficients for U251 cells treated with vehicle control (DMSO), 100 nM PTX, or 100 nM VBL (*n* = 58, 52, and 50 cells) are shown. (*B*) The velocities from the experiments in (*A*) are shown. (*C*) The motility coefficients from simulations using a reference parameter set (Table S1; k_nuc,0_ = 1 s^−1^) or in which k_nuc,0_ was increased (k_nuc,0_ = 10 s^−1^) are shown (*n* = 60, 28 simulations). All simulations had n_motor_ = 1000, n_clutch_ = 750, and *ψ*_pol_ = 0.9, and all other parameter values were reported in Table S1. (*D*). The velocities from the simulations in (*C*) are shown. Individual motility coefficients and velocities were obtained from fits to Eq. 2. The error bars represent mean ± SEM, NS denotes no significant difference, and *p* > 0.01 by Kruskal-Wallis one-way ANOVA. To see this figure in color, go online.

Dynamic microtubules establish and maintain the polarity of migrating cells (54), whereas microtubule-targeting agents (MTAs) disrupt microtubule-dependent polarity and directed migration (10). MTAs are widely used in chemotherapy and are distinguished by their effects on polymer assembly: 1) assembly promoters such as paclitaxel (PTX) and 2) disassembly promoters such as vinblastine (VBL) (55). MTAs’ most pronounced effect is kinetic stabilization, which reduces the accumulation of microtubule tip-tracking proteins such as EB1 to microtubule ends and is a common effect of both assembly-promoting and disassembly-promoting MTAs (55). We confirmed that U251 cells expressing EB1-eGFP had dynamic microtubules (Fig. S5), and microtubules exhibited growth speeds similar to earlier measurements in U251 cells on 2D unconfined substrates (10). Tracking U251 cells treated with either PTX or VBL (at 100 nM) revealed that both MTAs had little effect on motility coefficient (Fig. 7 *A*), but each significantly reduced velocity (Fig. 7 *B*) compared to vehicle (DMSO) controls. Interestingly, neither PTX nor VBL significantly affected either the cell length or nucleus position in the channels (Fig. S5), suggesting that MTAs do not significantly disrupt nucleus positioning or cell polarity and may influence cell migration through other means.

Balzer et al. (25) observed that EB1-labeled microtubule arrival at the leading edge was concomitant with leading edge protrusion in confinement, suggesting a direct correlation between microtubule impact and forward cell protrusion. We have also previously suggested that MTAs reduce maximal protrusion velocity (v_actin,max_) to impair migration in 2D (10), and our earlier 1D CMS results in which v_actin,max_ was reduced (Fig. 6, *A* and *B*) are in line with both of these observations. Alternatively, our earlier study (10) suggested that microtubules can influence the basal nucleation rate for new modules (k_nuc,0_ = 1 s^−1^; Table S1). Namely, these results suggest that increasing the number of protrusions slows motility in 2D, consistent with experimental MTA effects (10). Increasing the basal nucleation rate for new modules (k_nuc,0_ = 10 s^−1^) reduced the simulated cell motility coefficient (Fig. 7 *C*) and velocity (Fig. 7 *D*), suggesting that MTAs inhibit glioma cell migration by similar mechanisms on 2D substrates and in 1D confinement. We conclude that the 1D CMS can accurately predict mechanistic behaviors of MTAs and actin assembly inhibitors and that glioma cell migration in confinement relies upon dynamic cytoskeletal self-assembly.

## Discussion

Cells experience mechanical confinement while invading dense tissues in vivo. Similar confinement can be reliably reproduced in PDMS microchannel assays, and live-cell-tracking measurements can be compared to biophysical simulations of cell migration mechanics. Using simulation parameters calibrated to previous 2D measurements of human glioma cells as a starting point, we reproduced mean cell migration speeds close to ∼0.5 *μ*m per min^−1^, consistent with experiments. This corresponds to a tumor growth rate of 25 cm per year^−1^, within the range of tumor growth rates measured in the clinic (56). The 1D CMS also predicts the effects of pharmacological agents targeting components of the motor-clutch system, including a biphasic relationship between substrate adhesiveness and speed. These results suggest that glioma cells (and potentially other cell types) employ a motor-clutch force transmission mechanism to migrate within confined spaces.

In vivo, glioma cells likely employ CD44 clutches when engaging with the hyaluronic-acid-rich stroma (11). Integrins are also likely involved as cells interact with basement membranes that contain collagen and laminin (17). Invading cells could also employ friction-based amoeboid migration in environments with low ligand density. Frictional forces could be incorporated in the motor-clutch model by reducing the characteristic clutch bond force (F_bond_) and increasing the binding and unbinding rates (k_on_ and k_off_, respectively). Confinement appears to be required for adhesion-independent migration because the same cells move slowly in unconfined environments lacking adhesion molecules (18). To simulate this, the clutch number (n_clutch_) could be increased proportionally to the increase in contact area, increasing cell speed as they enter the “optimal” adhesion regime.

F-actin forms the basis of cellular protrusions in many cells, and the 1D CMS captures the dynamics of actin assembly and disassembly within modules. F-actin in modules is under tension between motor-based pulling forces and substrate deformation, so the model considers it to be rigid in accordance with the observed strain stiffening behavior of stress fibers and cross-linked actin gels (57). Furthermore, we consider F-actin to behave elastically because clutch binding and unbinding events occur on the ∼10^−1^–10^−3^ s timescale, which is faster than typical intracellular cross-linker lifetimes that give rise to viscoelastic behaviors within F-actin networks (57). Other physical models explicitly model F-actin networks as a viscoelastic fluid, accounting for energy dissipation through cross-linker binding and unbinding (26,58). Although this version of the 1D CMS does not include a viscoelastic term for the cytoskeleton, future works could address this by including time-varying mechanical elements within the cell body instead of the current elastic cell spring term (*κ*_cell_).

Osmotic-pressure-driven migration of tumor cells has previously been proposed as a mechanism for glioma dispersion (16). In osmotic-pressure-driven models of cell migration (23), asymmetrically distributed ion pumps at the leading and trailing edge create a net protrusive force on the forward-facing cell membrane, whereas friction between the flowing cortex and channel wall transmits forces. In this context, migrating cells are insensitive to actin polymerization inhibitors (such as LatA), suggesting that the osmotic engine supplants actin-based migration when hydraulic resistance would otherwise stall actin polymerization (26). Contrasting with this result, we conclude that hydrodynamic drag forces are minimal in our system (see Supporting Materials and Methods) and find that LatA stalled U251 cell migration in these channels (Fig. 6, *A* and *B*; Video S2). There are several nonexclusive explanations for these contrasting observations. First, the channels in (23) have a smaller cross-sectional area (30 *μ*m^2^ compared to 60 *μ*m^2^ in this study), suggesting that hydrodynamic drag forces are smaller in our system. Using the mean velocity that we experimentally measured for U251 cells (Fig. 3 *B*), fluid drag forces on individual cells in our channels are on the order of ∼6.5 pN (see calculation in Supporting Materials and Methods). This is orders of magnitude smaller than the total stall force in simulations (Table S1) or traction forces produced by adherent cells (30,37), suggesting that U251 cells can readily overcome hydrodynamic drag. Deformation of larger organelles, such as the nucleus, may effectively increase drag forces in narrower channels as well. Second, the osmotic engine appears to require particular ion transport and water flux proteins (23) that may vary in expression between cell lines and contribute to differential sensitivity to hydrodynamic drag. Third, some cells can internalize fluid through macropinocytosis to minimize hydrodynamic drag (59).

The 1D CMS includes a polarity parameter (*ψ*_pol_) to represent a directional bias along the channel axis. Empirically, we found that *ψ*_pol_ = 0.9 produced reasonably close fits to experimental data (see Fig. 2, *F* and *G*) and used this as our base value for the U251 parameter set in simulations (Table S1). Although we note that the constant polarity value may be a simplification of the underlying biological mechanism, removing the directional bias in polarity (*ψ*_pol_ = 0.5) yielded diffusive, nonpersistent simulated cell migration that was not observed experimentally (Fig. 1, *B* and *C*). Several examples of asymmetric cytoskeletal regulation exist in the literature that may point to a biological mechanism. Confinement polarizes the distribution of F-actin stress fibers and phosphorylated myosin II light chain in migrating glioma cells, which, in turn, polarize force generation along the channel axis (60). Microtubules are also involved in polarized cell migration in confined channels (25,32), where they may influence the activity of signaling proteins that regulate F-actin dynamics, such as Rho-family GTPases (54). A recent study examined the effect of microtubule-based delivery of Rho guanine nucleotide exchange factor (GEF) H1 on the dynamics of cellular protrusions (61). Asymmetric delivery of Rho GEF H1 by microtubules could consistently activate Rho GTPase at the leading edge of cells, thus driving asymmetric protrusion nucleation and polarized migration. We note that MTAs slowed U251 migration in channels (Fig. 7, *A* and *B*), although future work will be required to identify the signaling factors involved in this response. Regardless of the mechanism, recapitulating the behavior of cells in the channels is sensitive to the value of *ψ*_pol_, an effect that is not required for simulations of cells on 2D substrates using the same physical model of migration (9). This confinement-induced polarity may play a role in tumor progression by biasing cell movements away from the tumor bulk and into healthy tissue, as individual glioma cells are often observed migrating away from tumors in ex vivo slice cultures (11,13,14).

Photolithography and PDMS replica molding enabled production of micrometer-scale channels for parallel analysis of a large number of individual cell trajectories (∼1000 cells in a single study). One disadvantage of PDMS molding techniques is that devices are often made from materials with a high elastic modulus (E = ∼1000 kPa) and bound to glass dishes (E = ∼10^7^ kPa). These values are above the limit of stiffness sensitivity for U251 cells (9,40) and prohibit the measurement of traction forces, although we note that certain PDMS mixtures can yield significantly softer moduli (39). Pathak and Kumar (60) used photolithography to manufacture confined channels in polyacrylamide hydrogels, enabling them to independently control the channel width (w = 10–40 *μ*m) and device stiffness (E = 0.4–120 kPa). Interestingly, they reported biphasic cell speed as a function of hydrogel modulus, consistent with CMS predictions (9). However, the channels produced with this method only laterally confined the cell, and they did not produce any channel structures with dimensions <10 *μ*m, which are easily produced by PDMS replica molding.

Previous studies have inferred relationships between biophysical measurements in 2D substrates and confined migration behaviors (30,31) but did not employ simulated migration to test those predictions. In this study, we used motor-clutch parameters for glioma cells measured on 2D unconfined substrates (9,10) to predict confined migration behaviors. Altogether, our results suggest that the CMS can predict confined tumor cell migration, as well as antimotility therapy, using extant cell migration data. Future work could connect these individual cell behaviors to tumor-scale mathematical models (62,63), which could, in turn, provide inputs for multiscale models of tissue invasion and avenues for therapeutic intervention.

## Author Contributions

Conceptualization, L.S.P., P.V., M.P., and D.J.O.; methodology, all authors; software, L.S.P.; validation, L.S.P. and M.R.S.; formal analysis, L.S.P., M.R.S., and D.J.O.; resources, P.V., M.P., and D.J.O.; writing—original draft, L.S.P. and D.J.O.; writing—review and editing, all authors; visualization, L.S.P.; supervision, P.V., M.P., and D.J.O.; and funding acquisition, L.S.P., M.P., and D.J.O.

## Supporting Citations

References (64, 65, 66, 67, 68, 69, 70, 71, 72, 73) appear in the Supporting Material.

## Supporting Material

Document S1. Supporting Materials and Methods, Figs. S1–S5, and Tables S1–S2

Document S2. Article plus Supporting Material
