# Supplementary material for: Predicting Confined 1D Cell Migration from Parameters Calibrated to a 2D Motor-Clutch Model
Source: Biophys J. 2020 Feb 25;118(7):1709–20. doi: 10.1016/j.bpj.2020.01.048 (PMC7136340; doi:10.1016/j.bpj.2020.01.048)
Supplement: Document S1. Supporting Materials and Methods, Figs. S1–S5, and Tables S1–S2 [file mmc1.pdf]

Biophysical Journal, Volume *118*

Supplemental Information

Predicting Confined 1D Cell Migration from Parameters Calibrated to a 2D Motor-Clutch Model

Louis S. Prahl, Maria R. Stanslaski, Pablo Vargas, Matthieu Piel, and David J. Odde

Supporting Methods

1D cell migration simulator

Previous implementations of the cell migration simulator (CMS) either considered a cell migrating on a 2D unconfined surface with varying adhesiveness or mechanics (1–3), or explicitly modeled the underlying fiber network geometry (4). In the present study, we modified the CMS to model cell migration in a single spatial dimension, hereafter referred to as the 1D CMS (schematic is shown in **Figure 1A**). As with previous versions, the 1D CMS contains a number of protrusion modules (j represents the module number), each functioning as an instance of the motor-clutch model (5). Modules contain $n_{clutch,j}$ adhesion clutches, each of which is modeled as an elastic spring with stiffness κ_{clutch}. Clutches bind to a compliant substrate (also an elastic spring with stiffness κ_{sub}) at a reference point $x_{ref,j}$. Clutch binding occurs at a rate k_{on}, which is assumed to be a first-order reaction. Unbinding of the ith clutch in a particular ensemble occurs at a rate $k_{off,i}$ that scales with force by a single exponential (6), also known as a slip bond.

$$k_{off,i} = k_{off} exp\left(\frac{F_{clutch,i}}{F_{bond}}\right) \qquad \textbf{Eqn. S1}$$

Here, k_{off} is the unloaded unbinding rate, F_{bond} is the characteristic bond force, and $F_{clutch,i}$ is the force on the ith clutch. Modules also contain $n_{motor,j}$ myosin II motors, each of which is capable of generating F_{motor} stall force. Motors slide an F-actin bundle to generate retrograde flow (v_{flow}), which extends clutches that are bound to the substrate. As forces build on the substrate through bound clutches, F-actin flow slows from the myosin II motor unloaded velocity v_{motor} by a linear force-velocity relationship (5).

$$v_{flow} = v_{motor}\left(1 - \frac{\kappa_{sub}\Delta x_{sub,j}}{F_{motor}n_{motor,j}}\right) \qquad \textbf{Eqn. S2}$$

We note that individual actin filaments polymerizing against a tensed plasma membrane are also capable of generating F-actin retrograde flow and traction forces, so each filament can be regarded as a motor capable of generating pN-scale forces and velocities of ~100 nm s^{-1} in the absence of load. In addition, polymerizing actin filaments also obey a monotonic force-velocity relationship that can be approximated by **Eqn. S2**. Substrate spring displacement on the jth module is $\Delta x_{sub,j}$. Each module contains a rigid bar of F-actin with length $A_{F,j}$. Modules extend by actin polymerization (v_{actin}) from a soluble pool of G-actin subunits, A_G. Actin polymerization (v_{actin}) is defined by a maximum actin polymerization velocity $v_{actin,max}$, A_G, and the total available actin length in the cell, A_{total}.

$$v_{actin} = v_{actin,max}\left(\frac{A_G}{A_{total}}\right) \quad \textbf{Eqn. S3}$$

A mass balance constrains A_{total} and A_G in a simulated cell with n_{module} modules.

$$A_{total} = A_G + \sum_{j=1}^{n_{module}} A_{F,j} \quad \textbf{Eqn. S4}$$

F-actin is depolymerized when it passes the motor position within modules ($x_{motor,j}$) and returns to the G-actin pool in the cell. Module clutches also unbind when their bound position ($x_{clutch,i}$) extends past $x_{motor,j}$. Modules contain a compliant cell spring with stiffness κ_{cell} that represents the nucleo-cytoskeletal compliance of the cell and connects the module motors to the central cell body. A force balance applies to each module spring, clutches, and cell spring.

$$F_{module,j} = \kappa_{cell}\Delta x_{cell,j} = \kappa_{clutch}\sum_{i=1}^{n_{clutch,j}} \Delta x_{clutch,i} = \kappa_{sub}\Delta x_{sub,j} \quad \textbf{Eqn. S5}$$

Within the cell body, an ensemble of $n_{clutch,cell}$ clutches transmit force to the substrate under the cell body in the same way as module clutches. F_{cell} represents the force from extension of the cell substrate spring $\Delta x_{sub,cell}$

$$F_{cell} = \kappa_{clutch}\sum_{i=1}^{n_{clutch,j}} \Delta x_{clutch,i} = \kappa_{sub}\Delta x_{sub,cell} \quad \textbf{Eqn. S6}$$

Cell body clutches are not subject to direct myosin II forces from F-actin flow but extend as the cell position x_{cell} is updated by force balance on modules and the cell body.

$$F_{cell} + \sum_{j=1}^{n_{module}} F_{module,j} = 0 \quad \textbf{Eqn. S7}$$

Module capping occurs at a first-order rate k_{cap} and capped modules no longer extend by actin polymerization. Nucleation of new modules occurs at a rate k_{nuc}, and k_{nuc} is a function of the basal nucleation rate ($k_{nuc,0}$) and the available G-actin pool raised to the fourth power (7).

$$k_{nuc} = k_{nuc,0}\left(\frac{A_G}{A_{total}}\right)^4 \quad \textbf{Eqn. S8}$$

New modules have initial length L_{cell} and are assigned motors and clutches as a fraction of the available pool of each within the cell (1). Modules that shorten past a minimum length L_{min} are eliminated and their contents (motors, clutches, F-actin) are returned to the common cellular pool.

In the previous CMS, modules are randomly nucleated at a uniformly distributed angle θ within the 2D plane. Module nucleation in 1D can only occur in the +x or –x direction, with the probability of a new module being generated in the +x direction defined by a cytoskeletal polarity factor ψ_{pol}. The complementary probability 1-ψ_{pol} is the probability that a new module is generated in the –x direction. The case where ψ_{pol} = 0.5 represents an equal likelihood of module generation in the +x and –x directions, which can be thought of as a “non-polarized” cell. In a cell where ψ_{pol} < 0.5, the polarity would bias module nucleation in the –x direction. Essentially, the probability of module nucleation in the +x direction follows a Bernoulli distribution with parameter ψ_{pol}. All simulation parameter values are reported in **Table S1** and changes for specific conditions are described in the figure legends.

Simulation implementation and analysis

Event selection was determined using kinetic Monte Carlo (8) using a unique, randomly generated seed for each run. The order of events in each step of the model algorithm is previously described (1). Simulation initial conditions included two modules oriented in the +x direction and one module oriented in the –x direction (each module of length L_{cell}) and all clutches unbound. Individual runs represent data collected over 5-7 hours of simulated time. Simulations were run in Matlab (The MathWorks Inc., Natick, MA) on in-house computing cores or using high performance computing cores in the Minnesota Supercomputing Institute.

To quantify simulated cell migration, cell body position (x_{cell}) was sampled every 5 minutes of simulated time. We excluded the first hour of simulated data from analysis and subsequent data were filtered to remove large displacements in cell position that result in velocities >1 μm s^{-1} (1). MSD was calculated from cell body position using the overlap method (1, 9) and fit to cell motility models in order to extract parameters related to cell migration (described in the main text) using a custom Matlab analysis script. For motility model fitting, we used the first half of MSD versus time lag data for each individual cell (1).

Cell culture and transfection

Human U251 glioma cells were cultured in DMEM/F-12 (Gibco, ThermoFisher Scientific, Waltham, MA) supplemented with 10% (v/v) fetal bovine serum (Gibco) and 100 μg mL^{-1} penicillin/streptomycin antibiotic (Corning, Inc., Corning, NY), maintained in a humid incubator at 37°C and 5% CO_2, and passaged using 0.25% trypsin-EDTA (Corning). Transfection of fluorescent proteins was performed using FuGENE® HD transfection reagent (Promega Corporation, Madison, WI) at least 24 hours prior to seeding cells in devices, as described previously (3). Fluorescent fusion plasmids included eGFP-β-actin (gift from Paul Letourneau, University of Minnesota) and EB1-eGFP (gift from Lynne Cassimeris, Lehigh University; (10)).

Pharmacological agents

Microtubule-targeting agents vinblastine sulfate (Sigma-Aldrich Corp., St. Louis, MO) and paclitaxel (Sigma) were stored in stock concentrations of 1 mM in DMSO, Y-27632 dihydrochloride (Sigma) was stored as 15 mM stock in water, cyclo-(Arg-Gly-Asp) peptide (cRGD; Enzo Life Sciences Inc., Farmingdale, NY) was stored at 4 mM in PBS, and latrunculin A (Sigma) was stored at 0.5 mM in DMSO. All drug stocks were kept frozen at -20°C. DMSO volume added to dishes did not exceed 0.1% in any condition.

Microchannel device design and assembly

Devices feature three rectangular seeding ports (shown in **Figure 2A**) with 15 μm diameter pillars evenly spaced at 25 μm intervals and with opposite rows of channels aligned towards the device exterior. Entrances to channels are 100 μm long funnels that taper from an initial width of 18 μm to a final channel width of 12 μm. Chromium-plated quartz photomasks were generated by the Minnesota Nano Center (MNC) facilities. Device molds were manufactured on 4” silicon wafers using photolithography with epoxy-based SU-8 2005 negative photoresist (MicroChem Corp., Wesborough, MA).

Briefly, a 5 μm thick photoresist layer was spin coated on wafers using a CEE 100 spin coater (Brewer Science, Rolla, MO), followed by a 2 minute soft bake at 95°C. Wafer surfaces were exposed to UV radiation at 12 mJ s cm^{-2} for 10 seconds using a MA6 mask aligner (Karl Süss MicroTec, Garching, Germany). Following a 3-minute post-exposure bake at 95°C, excess photoresist was removed using SU-8 developer (MicroChem). After curing, the height of individual photoresist structures was verified using a P-16 stylus profilometer (KLA-Tencor, Milpitas, CA). To minimize PDMS adhesion and facilitate device removal, wafers were treated with trichloro(1H,1H,2H,2H-perfluorooctyl)silane (Sigma, St. Louis, MO) under vacuum before casting.

Sylgard® 184 PDMS elastomer (Dow Corning, Midland, MI) was mixed in a 10:1 (base:curing agent) ratio, poured on wafers or epoxy replicas and allowed to cure at 75°C for 2 hours. Devices were cut and peeled from molds, seeding ports were added using a 3 mm radius circular punch (Ted Pella Inc., Redding, CA), and devices were manually resized to a 1.5 x 1.0 cm footprint centered around the seeding ports using a razor blade. Cut and resized devices were cleaned with adhesive tape and 70% ethanol in a sonic bath for 1 minute and then allowed to dry under a stream of compressed air. Devices and 35 mm glass bottom dishes (20 mm No. 0 coverglass, MatTek Corp., Waltham, MA) were activated with a plasma cleaner (PDC-32 G, Harrick, Ithaca, NY) for 30 seconds, then bonded at 75°C for 1 hour.

Assembled devices were plasma treated for 1 minute and a solution of 10 μg mL^{-1} bovine plasma fibronectin (Sigma) in water was directly added to seeding ports. After 1 hour, devices were washed with PBS and stored overnight at 4°C before seeding cells. Devices were pre-incubated in media (containing drugs or DMSO, if appropriate) at 37°C for at least 1 hour prior to cell seeding. Cells were detached from flasks using 0.25% trypsin-EDTA (Corning), re-suspended in culture media, and nuclei were labeled using a nucleus counterstain (NucBlue Live, ThermoFisher) according to manufacturer instructions. Following labeling, cells were re-suspended in media to a density of $20x10^6$ cells per mL. Media was removed from device dishes and seeding ports, and a 5 μL aliquot of cell suspension (10^5 cells) was added to each seeding port. Devices containing cells were returned to the incubator for 30 minutes before adding sufficient media to cover the device inlets.

Time-lapse imaging and image processing

Devices were pre-incubated in media (containing drugs or DMSO) at 37°C for at least 1 hour prior to cell seeding. Cells were detached from flasks using 0.25% trypsin-EDTA (Corning) and re-suspended in culture media. Nuclei were labeled with NucBlue Live counterstain (ThermoFisher) according to manufacturer instructions. After centrifuging and re-suspending cells to a density of $20x10^6$ cells per mL, a 5 μL aliquot (10^5 cells) was added to each seeding port. Devices containing cells were returned to the incubator for 30 minutes before adding sufficient drug-containing media (~2 mL) to cover the entire device.

Time-lapse images were acquired on either a Nikon TiE or Nikon Ti2 epifluorescence microscope under control of NIS Elements software (Nikon Instruments Inc., Melville, NY) a ProScan III motorized stage (Prior Scientific Inc., Rockland, MA), a white light transmitted LED (CoolLED Ltd., Andover, UK), LED fluorescence illumination (Spectra X; Lumencor, Beaverton, OR), and DAPI/FITC/TxRed filter set (Chroma, Cat#89014), and a Zyla 4.2 or 5.5 sCMOS camera (Andor Technologies Ltd., Belfast, UK). Stable environmental control (5% CO_2 at 37°C) was provided using a stagetop incubator (OkoLab Llc., Ottaviano, Italy). Time-lapse images of cells in channels were acquired in the transmitted and DAPI channels every 5 minutes over 8-18 hours using a 20x/0.45NA phase I lens with 2x2 pixel binning (645 nm per pixel).

Images for analyzing cell length and nucleus position were acquired using a 40x/0.95NA phase II lens with no pixel binning (163 nm per pixel) in the FITC and DAPI channels. Cell length and nucleus position were measured manually using Fiji (11). First, cell images were oriented, so the leading and trailing edges were on the right and left of the image panel, respectively. Direction of motion was ascertained from a short time-lapse acquisition. Next, we subtracted background, and

a rectangular box was manually drawn around the cell boundaries. Cell and nucleus coordinates were measured from the center of mass using the measurement tool in Fiji. Normalized cell coordinates were defined as 0 being the cell rear (leftmost protrusion) and 1 being the cell front (rightmost protrusion), and normalized nucleus position was calculated as the ratio of the distance between the cell rear and nucleus centroid and the total length of the cell. Movies for microtubule growth rate were obtained from the FITC channel through a 100x/1.49 NA lens using streaming acquisition at 300 milliseconds per frame. Microtubule tip position for individual EB1-eGFP decorated plus-ends were measured with sub-pixel accuracy using TipTracker_v3 without modification (12). Individual microtubule growth velocities were calculated by dividing the mean length increment change by the acquisition time (0.3 s) as previously described (3).

Confocal z-stacks

Confocal z-stacks were acquired on an LSM7 LIVE swept-field confocal microscope (Carl Zeiss Microscopy, LLC; Jena, Germany) using a 40x/0.95 NA objective. The 488 nm and 405 nm lasers and BP 520-555 and BP 415-480 filters enabled simultaneous acquisition of the blue and green channels. Images were digitized using a 512-pixel 11-bit linear detector. Zen software (Zeiss) controlled the microscope system during imaging.

Semi-automated nucleus tracking

Nucleus position coordinates were obtained from the DAPI channel of image sequences using custom semi-automated image segmentation and tracking Matlab scripts (MathWorks) as previously described for tracking cells expressing fluorescent labels (2). Image sequences were cropped to include a single cell that was not in contact with another cell, as ascertained from phase contrast images. Skipped frames (e.g. due to contact with another cell or object in the channel) were omitted during analysis, as described previously (1). MSD were calculated from cell coordinates using the overlap method (9) and cell trajectories were analyzed using the same motility models as simulated cell trajectories.

Statistical analysis

Experimental data represent accumulated numbers of cells from $N \geq 2$ independent experiments for each condition. Numbers of simulations are reported in the figure legends; simulations start from a randomly generated seed and each simulation is considered independent for the calculation of n. For comparisons, a non-parametric Kruskal-Wallis one-way analysis of variance (ANOVA) was used since data did not typically follow a normal distribution. The Dunn-Sidăk test was employed to correct for multiple comparisons. Statistical significance for a particular comparison was reached when $p < 0.01$. All statistical analysis of experimental or simulated data was performed using the Matlab Statistics Toolbox (MathWorks).

Supplemental Notes

Mechanical properties and elastic behavior of F-actin, clutches and the cell spring

As with previous iterations of the motor-clutch model (5), F-actin is assumed to be rigid and inextensible. The cell spring (κ_{cell}) is connected in series with the F-actin bar, clutches, and substrate, representing the nucleo-cytoskeletal compliance of the cell. The chosen cell spring constant value (κ_{cell} = 10^4 pN nm^{-1}; **Table S1**) was used in previous studies employing the CMS (1), and is consistent with the rigid modulus measured for isolated actin filaments and stress fibers (E ~ 10^4 kPa) (13).

We assumed elastic behavior of the cell spring, since simulations generate ~10-100 pN forces (based on the total number of myosin II motors allocated to a module), which result in small (<1 nm) deformations. Larger forces that could significantly deform the cell may induce non-linear (strain stiffening) or visco-elastic behaviors of F-actin networks (13). Modeling these would require more detailed theoretical treatment of the F-actin properties, but in the present study we did not observe any cell deformations that could not be explained due to actin-based extension and retraction of cell protrusions. The microfluidic devices used in the present study are assembled from rigid PDMS (E = 10^3 kPa as measured in (14)) and glass (E ~ 10^6 kPa) and thus are not significantly deformed by cells, so we assumed an elastic spring best captured their behavior. Clutches are similarly treated as elastic springs, since individual clutch binding and unbinding occurs on the milliseconds timescale, allowing us to ignore short viscous relaxation times (5).

Estimation of hydrodynamic drag within channels

Some cells are reportedly able to use osmotic pressure-based motility to drive actin-independent confined migration (15). This enables them to migrate even through micrometer-scale pores that limit protrusion by F-actin, and where hydrostatic pressure would typically preclude movement. In the present study, we report that U251 cells are sensitive to actin polymerization inhibition by latrunculin A (**Figure 6**) causing us to conclude that these cells do not use an osmotic pressure-based migration mechanism. Nevertheless, we sought to determine the magnitude of hydrodynamic drag in U251 cells to evaluate whether it should be included as a force in the model.

Li and Sun (16) estimate the hydrodynamic drag coefficient (d_g) for a cell within a square channel with dimension b and length l, filled with a medium of viscosity μ.

$$d_g = \frac{12\mu L}{b^2} \qquad \textbf{Eqn. S9}$$

The channels in our devices have a horizontal width of 12 μm and 5 μm height, yielding a 60 μm^2 cross-sectional area. Approximating the channel cross section as a 7.5 μm square, and estimating 1 mm total channel length, and medium viscosity equal to water ($\mu = 10^{-3}$ Pa s), **Eqn. S9** yields d_g = 0.21 pN s μm^{-3} for hydrodynamic drag coefficient.

Hydrodynamic force can be calculated for a cell moving with velocity v_{cell} through a channel with cross sectional area A.

$$F_{drag} = -d_g A v_{cell}$$ **Eqn. S10**

Using the value of d_g calculated by **Eqn. S9**, the mean velocity obtained from experiments (v_{cell} = 0.51 μm s^{-1}; **Figure 3B**) and assuming the same cross-sectional area as before, **Eqn. S10** produces a drag force F_{drag} = -6.5 pN. This value is of similar magnitude to the force generated by ~4 myosin II motors, and much smaller than cellular traction force estimates (17), so we consider hydrodynamic drag as negligible in our experimental system.

Supporting Tables

Table S1. 1D CMS base parameter set.

Parameter	Description	Value	Source
Motor parameters			
n_{motor}	Number of myosin II motors per cell	10^3	Adjustable, ~10^2-10^5 required for nN total traction force (17)
$n_{motor,module}$	Maximum number of myosin II motors per module	100	Adjustable, constrained to 0.1*n_{motor} (1)
F_{motor}	Stall force for a single myosin II motor	2 pN	Measured *in vitro* by optical tweezers (18)
v_{motor}	Maximum (unloaded) myosin II sliding velocity on F-actin	120 nm s^{-1}	Measured in (1); ~100 nm s^{-1} observed in other cells (5, 19)
Clutch parameters			
n_{clutch}	Number of clutch bonds (e.g. integrin-mediated adhesion complexes) per cell	750	Adjustable, $n_{clutch} \approx n_{motor}$ by theoretical estimate (20)
$n_{clutch,module}$	Maximum number of clutch bonds per module	75	Adjustable, 0.1*n_{clutch} (1)
F_{bond}	Characteristic slip bond force for clutches	2 pN	Measured *in vitro* by optical tweezers (21)
κ_{clutch}	Spring constant of the clutch spring	0.8 pN nm^{-1}	Theoretical estimate, on the order of 1 pN nm^{-1} (20)
k_{on}	Pseudo-first order association rate between clutches and F-actin	1 s^{-1}	Theoretical estimate, 10*k_{off} (22)
k_{off}	Basal (unloaded) first-order clutch dissociation rate from F-actin	10^{-1} s^{-1}	Minimum unbinding rate measured by photobleaching (23)
Substrate parameters			
$\kappa_{csubstrate}$	Substrate spring stiffness	10^3 pN nm^{-1}	Estimated PDMS modulus (14)
Cell body and actin parameters			
k_{cap}	Module capping rate	10^{-3} s^{-1}	Experimental estimate (1, 24)
$k_{nuc,0}$	Maximum module nucleation rate	1 s^{-1}	Experimental estimate (1, 3)
$v_{actin,max}$	Maximum actin polymerization velocity	200 nm s^{-1}	Experimental estimate (3, 5)
A_{total}	Total actin pool available for protrusions	100 µm	Estimate of total cell protrusion length (1)
κ_{cell}	Spring constant of the cell spring	10^4 pN nm^{-1}	Adjustable, estimated in (1)
L_{cell}	Initial module F-actin length	5 µm	Simulation initial condition
L_{min}	Minimum module F-actin length	100 nm	Adjustable
$n_{clutch,cell}$	Number of cell body clutches	10	Adjustable, estimated in (1)
ψ_{pol}	Cell polarity factor	0.9	This study

Table S2. Pairwise p-values for the experimental and simulation conditions involving inhibitors of integrin clutches and myosin II activation. Related to **Figure 4** and **Figure 5**.

Figure 6A. 1D CMS motility (n_{motor} = 1000, n_{clutch} varies; ψ_{pol} = 0.9)

n_{clutch}	25	75	250	750
8	0.95	0.02	1	0.06
25		0.01	0.98	0.63
75			0.80	10^{-4}
250				0.21

Figure 6B. 1D CMS velocity (n_{motor} = 1000; n_{clutch} varies; ψ_{pol} = 0.9)

n_{clutch}	25	75	250	750
8	0.05	10^{-8}	0.63	0.56
25		0.37	0.99	0.007
75			0.39	10^{-5}
250				0.09

Figure 6C. cRGD experiment motility

[cRGD]	0.1	0.3	1
0	0.54	0.97	0.67
0.1		0.99	0.09
0.3			0.41
1			

Figure 6D. cRGD experiment velocity

[cRGD]	0.1	0.3	1
0	0.0012	0.00088	10^{-14}
0.1		0.99	$<10^{-15}$
0.3			$<10^{-15}$
1			

Figure 6E. 1D CMS motility (n_{motor} = 1000; n_{clutch} = 750; ψ_{pol} = 0.9)

n_{motor}	500	750
100	0.99	10^{-4}
500		0.0028
750		

Figure 6F. 1D CMS velocity (n_{motor} = 1000; n_{clutch} = 750; ψ_{pol} = 0.9)

n_{motor}	500	750
100	0.099	0.0079
500		0.88
750		

Supporting Figures

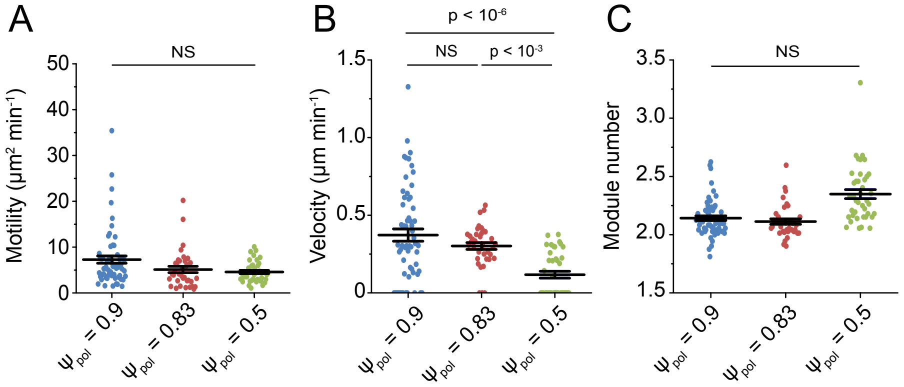

Figure S1. Simulated motility coefficient, velocity, and module number for a 1D cell migration simulator with variable polarity. Related to **Figure 1**.

Motility coefficients (**left**), velocities (**center**), and module number (**right**) for the simulations in **Figure 1** where ψ_{pol} = 0.5, 0.83, or 0.9, n = 60, 36, 40 simulations. Individual motility coefficients and velocities were obtained from fits to **Eqn. 2** in the **Main Text**. Error bars represent mean ± SEM. Pairwise statistics were computed by a Kruskal-Wallis one-way ANOVA with Dunn-Sidăk correction for multiple comparisons, NS = no significant difference between groups, $p > 0.01$. All simulations were run with n_{motor} = 1000 and n_{clutch} = 750, all other parameter values reported in **Table S1**.

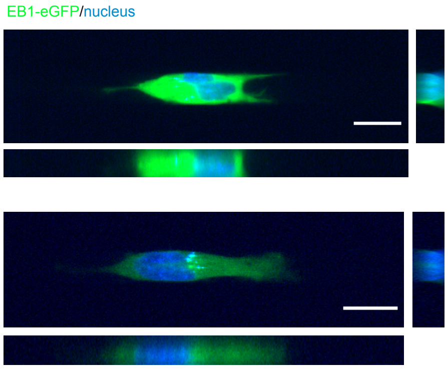

Figure S2. Confocal image stacks of cells in microchannels. Related to **Figure 2**.

A. Example confocal z-stacks of U251 cells expressing EB1-eGFP (green) and nucleus stain (blue). Images were acquired at 40x magnification. Images were oriented such that the channel inlets are to the left, while the outlets are to the right and adjusted for brightness/contrast in both green and blue channels. A view of the x-y plane is shown left, and an x-y slice through the center is shown at right. Total z-stack height, 11.43 μm; horizontal scale, 20 μm.

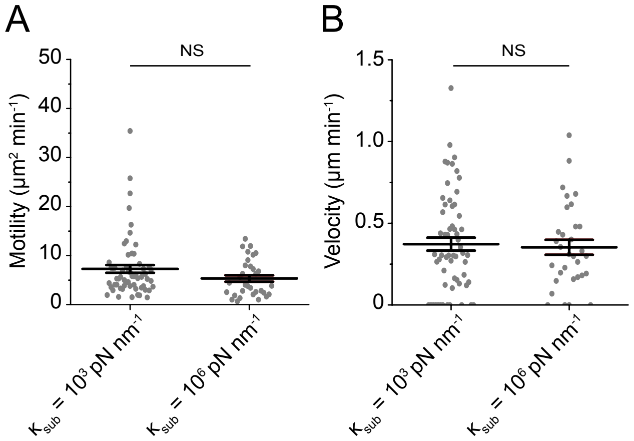

Figure S3. Effects of varying substrate spring constant on simulated cell motility coefficient and speed. Related to **Figure 1**.

Motility coefficients (**left**) and velocities (**right**) for simulations where $\kappa_{sub} = 10^3$ pN nm^{-1} or $\kappa_{sub} = 10^6$ pN nm^{-1}, n = 60, 24 simulations. Individual motility coefficients and velocities were obtained from fits to **Eqn. 2** in the **Main Text**. Error bars represent mean ± SEM. Pairwise statistics were computed by the Mann-Whitney U test, NS = no significant difference between groups, $p > 0.01$. All simulations were run with $n_{motor} = 1000$, $n_{clutch} = 750$, and $\psi_{pol} = 0.9$, all other parameter values reported in **Table S1**.

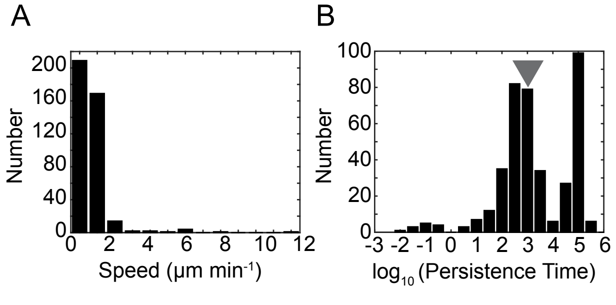

Figure S4. Persistent random walk fits predict persistence times that are longer than the experimental imaging duration. Related to **Figure 2** and **Figure 3.**

A. Speeds obtained from fits to a persistent random walk model (**Eqn. 1** in the **Main Text**), n = 403 cells from 12 independent experiments. S = 0.74 ± 0.05 μm min^{-1} or S = 12.3 ± 0.8 nm s^{-1} (mean ± SEM).

B. Persistence time (log_{10}-transformed) for the data in panel **A**. Arrow indicates the maximum experimental imaging duration of 18 hours or 1080 minutes ($log_{10}(1080) = 3.03$). Individual cell trajectories yielded a mean log_{10}-transformed persistence time of 3.1 ± 0.25 (mean ± SEM), corresponding to 1258 minutes.

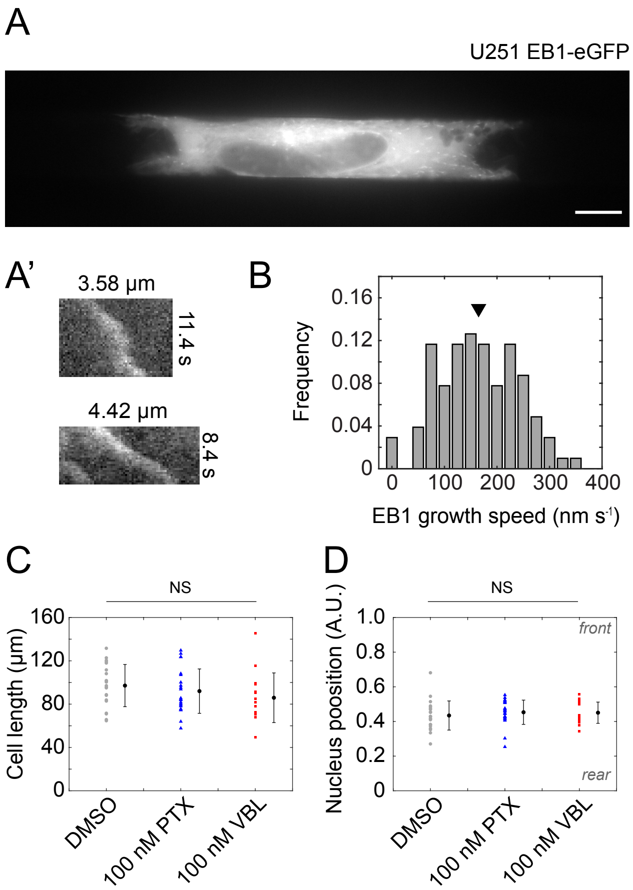

Figure S5. Measurements of microtubule dynamics as related to cell length and nucleus position in channels. Related to **Figure 7**.

A. Example image of a U251 cell in a microchannel device expressing EB1-eGFP. Image was captured at 100x magnification. Scale bar, 10 μm. **A'**. Example kymographs showing microtubule plus-end dynamics in U251 cells within channels. Microtubule growth speeds were measured for n = 103 microtubules in N = 5 cells using TipTracker software (12).

B. Histogram of microtubule growth speeds measured in U251 cells expressing EB1-eGFP. Black arrow marks a mean microtubule growth speed of 165 ± 7.5 nm s^{-1} (± S.E.M.).

C. Lengths of cells expressing EB1-eGFP and stained with NucBlue nucleus dye and treated with vehicle (DMSO), 100 nM PTX, or 100 nM VBL. Data points represent n = 21, 22, 15 measurements collected from 3 independent experiments.

D. Nucleus position for the cells in panel **C**. Cell lengths are normalized so that 0 marks the cell rear (trailing protrusion) and 1 denotes the cell front (leading protrusion). Error bars in panels **C** and **D** are mean ± standard deviation. Kruskal-Wallis one-way ANOVA revealed no significant differences (NS) between groups in panels **C** and **D**.

Supporting Movies

Movie S1. U251 glioma cells with fluorescently labeled nuclei migrating in microchannel devices.

Time-lapse images were collected every 5 minutes at 20x magnification with 2x2 binning (645 nm spatial sampling). Images were acquired in both the transmitted channel using phase contrast optics and LED fluorescence excitation (395 nm) using a DAPI/FITC/TxRed filter set. Scale bar, 50 μm.

Movie S2. U251 glioma cells expressing EGFP-actin and treated with vehicle control or latrunculin A migrating in microchannel devices.

Time-lapse images were collected every 5 minutes at 20x magnification with 2x2 binning (645 nm spatial sampling). Images were acquired in both the transmitted channel using phase contrast optics and using LED fluorescence excitation (395 nm and 470 nm) through a DAPI/FITC/TxRed filter set. Conditions include DMSO vehicle (top), 50 nM latrunculin A (middle), and 500 nM latrunculin A (bottom). Scale bar, 50 μm.

Supporting References

1. Bangasser, B.L., G.A. Shamsan, C.E. Chan, K.N. Opoku, E. Tüzel, B.W. Schlichtmann, J.A. Kasim, B.J. Fuller, B.R. McCullough, S.S. Rosenfeld, and D.J. Odde. 2017. Shifting the optimal stiffness for cell migration. Nat. Commun. 8: 1–10.
2. Klank, R.L., S.A. Decker Grunke, B.L. Bangasser, C.L. Forster, M.A. Price, T.J. Odde, K.S. SantaCruz, S.S. Rosenfeld, P. Canoll, E.A. Turley, J.B. McCarthy, J.R. Ohlfest, and D.J. Odde. 2017. Biphasic Dependence of Glioma Survival and Cell Migration on CD44 Expression Level. Cell Rep. 18: 23–31.
3. Prahl, L.S., P.F. Bangasser, L.E. Stopfer, M. Hemmat, F.M. White, S.S. Rosenfeld, and D.J. Odde. 2018. Microtubule-Based Control of Motor-Clutch System Mechanics in Glioma Cell Migration. Cell Rep. 25: 2591-2604.e8.
4. Estabridis, H.M., A. Jana, A. Nain, and D.J. Odde. 2018. Cell Migration in 1D and 2D Nanofiber Microenvironments. Ann. Biomed. Eng. 46: 392–403.
5. Chan, C.E., and D.J. Odde. 2008. Traction dynamics of filopodia on compliant substrates. Science (80-.). 322: 1687–1691.
6. Bell, G.I. 1978. Models for the specific adhesion of cells to cells. Science. 200: 618–27.
7. Tobacman, L.S., and E.D. Korn. 1983. The kinetics of actin nucleation and polymerization. J. Biol. Chem. . 258: 3207–3214.
8. Gillespie, D.T. 1977. Exact stochastic simulation of coupled chemical reactions. J. Phys. Chem. 81: 2340–2361.
9. Dickinson, R.B., and R.T. Tranquillo. 1993. Optimal estimation of cell movement indices

from the statistical analysis of cell tracking data. AIChE J. 39: 1995–2010.

10. Seetapun, D., B.T. Castle, A.J. McIntyre, P.T. Tran, and D.J. Odde. 2012. Estimating the microtubule GTP cap size in vivo. Curr. Biol. 22: 1681–1687.
11. Schindelin, J., I. Arganda-Carreras, E. Frise, V. Kaynig, M. Longair, T. Pietzsch, S. Preibisch, C. Rueden, S. Saalfeld, B. Schmid, J.-Y. Tinevez, D.J. White, V. Hartenstein, K. Eliceiri, P. Tomancak, and A. Cardona. 2012. Fiji: an open-source platform for biological-image analysis. Nat. Methods. 9: 676–82.
12. Prahl, L.S., B.T. Castle, M.K. Gardner, and D.J. Odde. 2014. Quantitative analysis of microtubule self-assembly kinetics and tip structure. Methods Enzymol. 540: 35–52.
13. Stricker, J., T. Falzone, and M.L. Gardel. 2010. Mechanics of the F-actin cytoskeleton. J. Biomech. 43: 9–14.
14. Steucke, K.E., P. V Tracy, E.S. Hald, J.L. Hall, and P.W. Alford. 2015. Vascular smooth muscle cell functional contractility depends on extracellular mechanical properties. J. Biomech. 48: 3044–3051.
15. Stroka, K.M., H. Jiang, S.-H.H. Chen, Z. Tong, D. Wirtz, S.X. Sun, and K. Konstantopoulos. 2014. Water permeation drives tumor cell migration in confined microenvironments. Cell. 157: 611–623.
16. Li, Y., and S.X. Sun. 2018. Transition from actin-driven to water-driven cell migration depends on external hydraulic resistance. Biophys. J. 114: 2965–2973.
17. Mekhdjian, A.H., F. Kai, M.G. Rubashkin, L.S. Prahl, L.M. Przybyla, A.L. McGregor, E.S. Bell, J.M. Barnes, C.C. DuFort, G. Ou, A.C. Chang, L. Cassereau, S.J. Tan, M.W. Pickup, J.N. Lakins, X. Ye, M.W. Davidson, J. Lammerding, D.J. Odde, A.R. Dunn, and V.M. Weaver. 2017. Integrin-mediated traction force enhances paxillin molecular associations and adhesion dynamics that increase the invasiveness of tumor cells into a three-dimensional extracellular matrix. Mol. Biol. Cell. 28: 1467–1488.
18. Molloy, J.E., J.E. Burns, B. Kendrick-Jones, R.T. Tregear, and D.C.S. White. 1995. Movement and force produced by a single myosin head. Nature. 378: 209–212.
19. Elosegui-Artola, A., R. Oria, Y. Chen, A. Kosmalska, C. Pérez-González, N. Castro, C. Zhu, X. Trepat, and P. Roca-Cusachs. 2016. Mechanical regulation of a molecular clutch defines force transmission and transduction in response to matrix rigidity. Nat. Cell Biol. 18: 540–548.
20. Bangasser, B.L., S.S. Rosenfeld, and D.J. Odde. 2013. Determinants of maximal force transmission in a motor-clutch model of cell traction in a compliant microenvironment. Biophys. J. 105: 581–592.
21. Jiang, G., G. Giannone, D.R. Critchley, E. Fukumoto, and M.P. Sheet. 2003. Two-piconewton slip bond between fibronectin and the cytoskeleton depends on talin. Nature. 424: 334–337.

22. Bangasser, B.L., and D.J. Odde. 2013. Master equation-based analysis of a motor-clutch model for cell traction force. Cell. Mol. Bioeng. 6: 449–459.
23. Lele, T.P., C.K. Thodeti, J. Pendse, and D.E. Ingber. 2008. Investigating complexity of protein-protein interactions in focal adhesions. Biochem. Biophys. Res. Commun. 369: 929–934.
24. Schafer, D.A., P.B. Jennings, and J.A. Cooper. 1996. Dynamics of capping protein and actin assembly in vitro: Uncapping barbed ends by polyphosphoinositides. J. Cell Biol. 135: 169–179.
