# Supplementary material for: Predicting Confined 1D Cell Migration from Parameters Calibrated to a 2D Motor-Clutch Model
Source: Biophys J. 2020 Feb 25;118(7):1709–20. doi: 10.1016/j.bpj.2020.01.048 (PMC7136340; doi:10.1016/j.bpj.2020.01.048)
Supplement: Document S2. Article plus Supporting Material [file mmc4.pdf]

Biophysical Journal
Article

Predicting Confined 1D Cell Migration from Parameters Calibrated to a 2D Motor-Clutch Model

Louis S. Prahl,[1] Maria R. Stanslaski,[1] Pablo Vargas,[2,3] Matthieu Piel,[2] and David J. Odde[1,4,*]
[1]Department of Biomedical Engineering, University of Minnesota, Minneapolis, Minnesota; [2]Institut Curie, PSL Research University, CNRS UMR 144 and Institut Pierre-Gilles de Gennes, PSL Research University, Paris, France; [3]INSERM U932 Immunité et Cancer, Institut Curie, PSL Research University, Paris, France; and [4]Physical Sciences-Oncology Center, University of Minnesota, Minneapolis, Minnesota

ABSTRACT Biological tissues contain micrometer-scale gaps and pores, including those found within extracellular matrix fiber networks, between tightly packed cells, and between blood vessels or nerve bundles and their associated basement membranes. These spaces restrict cell motion to a single-spatial dimension (1D), a feature that is not captured in traditional in vitro cell migration assays performed on flat, unconfined two-dimensional (2D) substrates. Mechanical confinement can variably influence cell migration behaviors, and it is presently unclear whether the mechanisms used for migration in 2D unconfined environments are relevant in 1D confined environments. Here, we assessed whether a cell migration simulator and associated parameters previously measured for cells on 2D unconfined compliant hydrogels could predict 1D confined cell migration in microfluidic channels. We manufactured microfluidic devices with narrow channels (60-μm^2 rectangular cross-sectional area) and tracked human glioma cells that spontaneously migrated within channels. Cell velocities ($v_{exp} = 0.51 \pm 0.02$ μm min^{-1}) were comparable to brain tumor expansion rates measured in the clinic. Using motor-clutch model parameters estimated from cells on unconfined 2D planar hydrogel substrates, simulations predicted similar migration velocities ($v_{sim} = 0.37 \pm 0.04$ μm min^{-1}) and also predicted the effects of drugs targeting the motor-clutch system or cytoskeletal assembly. These results are consistent with glioma cells utilizing a motor-clutch system to migrate in confined environments.

SIGNIFICANCE Cells migrating through dense tissues encounter micrometer-scale pores and track-like structures, which contribute additional forces to the cell that are not present in two-dimensional unconfined environments. It is presently unclear whether established models for cell migration apply to confined environments or whether cells adopt specialized mechanisms of force generation to navigate these environments. Simulated cell migration behaviors using a motor-clutch mechanism are consistent with human glioma cell migration in confined one-dimensional microfluidic channels. Simulations can also predict effects of drugs targeting integrin-mediated adhesion, myosin II motors, or cytoskeletal assembly dynamics. Our results suggest that glioma cells employ similar mechanisms in one-dimensional confined channels as on two-dimensional unconfined surfaces.

INTRODUCTION

Cell migration is involved in numerous physiological functions throughout organismal development and adult life. Aberrant cell migration disperses cancer cells into healthy tissue, which creates significant challenges in treating invasive gliomas (1) and other malignant tumors. The physical mechanism of cell migration involves coordinated dynamics of the actin cytoskeleton and adhesion complexes (2,3). F-actin assembly drives the elongation of cellular protrusions, whereas within protrusions, adhesion receptors (termed "clutches") assemble into complexes and link cells to extracellular matrix (ECM) ligands. Bound clutches cooperatively transmit contractile actin-myosin forces to the ECM, establishing traction forces that drive locomotion.

Integrated physical models incorporate mathematical expressions for these molecular processes to successfully predict experimentally measured cell migration behaviors (3–5). Building upon an established motor-clutch model for cell traction (6–8), a recently developed computational cell migration simulator (CMS) reproduces the characteristic random motility of glioma cells on compliant

Submitted January 2, 2019, and accepted for publication January 27, 2020.
*Correspondence: oddex002@umn.edu
Louis S. Prahl's present address is Department of Bioengineering, University of Pennsylvania, Philadelphia, Pennsylvania.
Editor: Vivek Shenoy.
https://doi.org/10.1016/j.bpj.2020.01.048
© 2020 Biophysical Society.
This is an open access article under the CC BY license (http://creativecommons.org/licenses/by/4.0/).

hydrogels (9,10). Further studies reproduced glioma cell migration behaviors in environments with varying adhesiveness (11) and complex fiber orientation (12,13) by changing relevant environmental parameters. However, these studies did not consider the mechanical confinement encountered by invading glioma cells as they escape the tumor bulk (14) and encounter an ECM composed of hyaluronic acid and densely packed cells (15,16). Cells invading along perivascular spaces and the glia limitans encounter micrometer-sized gaps between these structures and rigid basement membranes composed of collagen IV and laminin (16,17). Confinement is thus an important consideration in developing theoretical models of in vivo cell migration.

In vitro studies reveal varied, and sometimes surprising, cellular responses to confinement, including bleb-based fast amoeboid migration (18–20) or impaired movement of the nucleus through micrometer-scale pores (21,22). Adding to this complexity, pharmacological agents that inhibit migration on unconfined two-dimensional (2D) substrates are sometimes reported to have little or no effect on cells migrating in confined environments (23–25). Correspondingly, a number of biophysical theories have been proposed to explain specific behaviors, including adhesion-independent migration by frictional forces (19) and actin-independent protrusive forces provided by osmotic pressure gradients (23,26). These models are largely supported by data obtained in confinement and untested in other contexts.

Traditional cell migration assays are typically performed on glass or polystyrene dishes or hydrogels, which do not confine cells, raising the question of how relevant the mechanisms used in unconfined contexts are to confined environments. ECM proteins deposited into lanes (27), microgrooved silicon wafers (28), and suspended polystyrene fibers (12) replicate the one-dimensional (1D) aligned structures found in these regions (17). Cell contact guidance along these structures notably increases the directional persistence of cells (12,27), but these cells do not experience mechanical confinement as they would in three-dimensional tissue environments. To overcome these limits, photolithography and polydimethylsiloxane (PDMS) replica molding can create confined channels that also permit microscopy-based measurements of intracellular structure and dynamics (21–24,29–34).

To test confined cell migration predictions using a motor-clutch model, we created a 1D CMS to recapitulate directional guidance cues found in aligned brain tissue structures and linear microchannel designs. Tracking individual glioma cells in PDMS channels revealed spontaneous and persistent cell migration along the channel axis, which simulations predicted using parameter sets measured for cells on unconfined hydrogels (9,10). By changing relevant parameters, the 1D CMS also successfully predicted migration phenotypes of cells exposed to pharmacological agents targeting various motor-clutch components or cytoskeletal assembly dynamics. These combined results are consistent with glioma cells employing a motor-clutch mechanism to migrate in confined microfluidic channels.

MATERIALS AND METHODS

1D CMS

The 1D CMS employed in this study is modified from a previously described 2D CMS (9–12). A detailed model description can be found in the Supporting Materials and Methods. Simulation parameters and their estimated or measured values are reported in Table S1. Simulations were coded and run in MATLAB (The MathWorks, Natick, MA). Cell body position (x_{cell}) was recorded from simulations, and mean-squared displacement (MSD) was computed from x_{cell} using the overlap method (35).

Microchannel devices

Microchannel devices were based on previous designs (36) and were drawn using a computer-aided design software (AutoCAD; Autodesk, San Rafael, CA). Quartz-chrome photomasks containing the device patterns were produced from these designs using Minnesota Nano Center facilities and were used to create master molds for device designs on silicon wafers using standard photolithography techniques. Photolithography, PDMS replica molding, and device assembly are described in detail in the Supporting Materials and Methods.

Cell culture and imaging

Human U251 glioma cells were cultured at 5% CO_2 and 37°C. Culture media consisted of Dulbecco's modified eagle media/F12 (Gibco, Thermo Fisher Scientific, Waltham, MA) supplemented with 10% (v/v) fetal bovine serum (Gibco), 100 U mL^{-1} penicillin (Corning, Corning, NY), and 100 μg mL^{-1} streptomycin (Corning). Cells were passaged and subcultured using 0.25% trypsin-EDTA (Corning). Fluorescent proteins (eGFP-β-actin or end-binding protein 1 (EB1)-eGFP) were transiently transfected into cells as described previously (10). Nuclei were labeled using NucBlue Live ReadyProbes reagent (Thermo Fisher Scientific) before seeding cells in device inlets. Images of migrating cells were acquired every 5 min at 20× magnification on a Nikon Eclipse Ti2 or Ti-E microscope (Nikon Instruments, Melville, NY) under control of NIS-Elements Advanced Research software (Nikon). Individual cell nuclei positions were measured at each frame using a custom analysis script (11). MSDs were computed from experimental trajectories in the same way as simulations. Full details on experimental procedures and data analysis are described in the Supporting Materials and Methods.

Statistical analysis

Numbers of measurements are given in figure legends. Kruskal-Wallis one-way analysis of variance (ANOVA) was used unless otherwise noted.

RESULTS

Switch between random and persistent simulated migration in 1D is a function of asymmetric protrusion nucleation probabilities

In the previously described CMS, a force balance between stochastically nucleated cell protrusion modules and the cell body drives random motility in 2D substrates (9). For this study, we modified the CMS to solve for cell coordinates

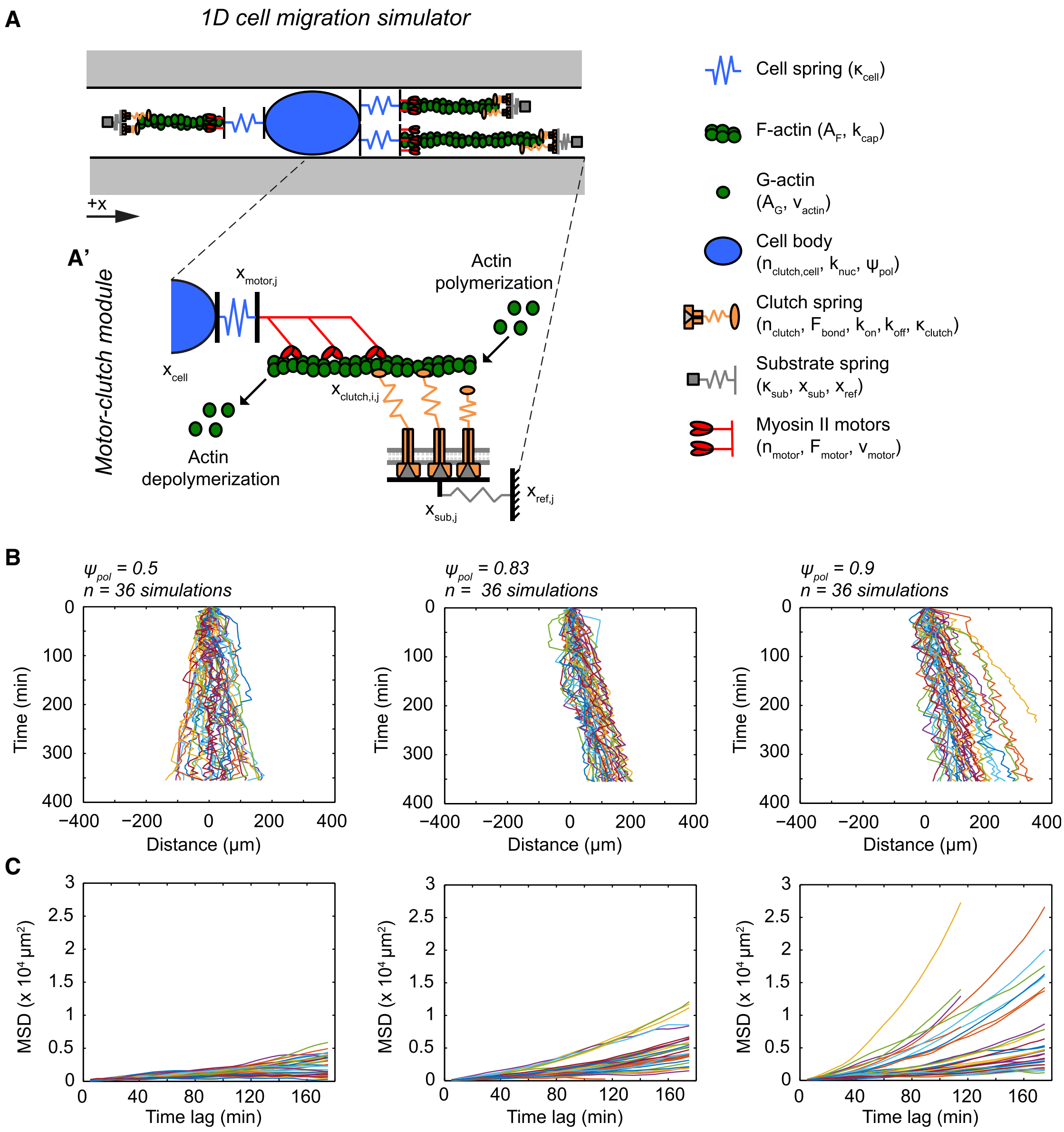

FIGURE 1 Description and migration dynamics of a 1D CMS. (*A*) A schematic of a 1D CMS within a confined channel whose axis is denoted by the *x* axis is given; gray boxes denote channel walls. Modules containing myosin II motors (n_{motor}) and adhesion clutches (n_{clutch}) attach to a central cell body through compliant springs. F-actin retrograde flow by myosin II motors and adhesion clutches are governed by similar rules to those described for previous iterations of the motor-clutch model (6,40). Cell body clutches (not pictured) associate with the cell center x_{cell} and undergo binding and unbinding as module clutches but are not subject to direct forces by F-actin retrograde flow. Each module contains an F-actin bundle ($A_{F,j}$ for the length of the jth module bundle) to which clutches bind. The total available G-actin in the cell (A_G) constrains module nucleation (with base rate constant $k_{nuc,0}$, governed by Eq. S8) and scales actin polymerization speed at the end of modules (maximal speed is $v_{actin,max}$, governed by Eq. S3). Module capping (k_{cap}) terminates polymerization and facilitates module shortening and turnover, whereas ψ_{pol} gives the probability of new protrusions being generated in the $+x$ direction. The number of modules nucleated by a given cell is not constrained, and multiple overlapping modules at the leading or trailing edge of the cell is permitted and denoted by cell springs (κ_{cell}) drawn in parallel. (*A′*) The inset shows a schematic of a single module (i.e., the jth module) within the simulation. Within the jth module, the distal end of the substrate spring is at a reference point $x_{ref,j}$, whereas the other end serves as the anchoring point for the clutch ensemble at $x_{sub,j}$. The ensemble of $n_{clutch,j}$ clutches within the jth module attaches to the F-actin filament, and $x_{clutch,j}$ represents the average location of the extended clutch springs. Actin polymerizes at the distal end of modules and depolymerizes when it passes the motor ensemble, located at $x_{motor,j}$. Movement of the cell body (x_{cell}, pictured as the center of the nucleus) is governed by force balances on each module and the cell body clutches (see, Eqs. S5–S7). (*B*) The simulation position is shown as a function of time for individual 1D CMS runs in which $\psi_{pol} = 0.5$–0.9 ($n = 36$ simulated trajectories for each condition). The initial position is marked at $x(\tau = 0) = 0$. (*C*) The MSD versus time lag is shown for the 1D CMS trajectories in (*B*). All simulations were run with $n_{motor} = 1000$ and $n_{clutch} = 750$; all other parameter values reported in Table S1.

within a single-spatial dimension (1D CMS; Fig. 1 *A*), and the model and underlying equations are fully described in the Supporting Materials and Methods. Each simulated cell contains an ensemble of n_{motor} myosin II motors, n_{clutch} molecular adhesion clutches, and a total pool of F-actin (A_{total}) that form the basis for protrusion modules. Individual motors each produce a stall force of F_{motor}. The motor ensemble drives retrograde flow of F-actin bundles within modules at a maximal unloaded velocity (v_{motor}), which decreases under the load by a linear force-velocity relationship (6), as described in Eq. S2. We note that actin polymerization and myosin II motors both obey a monotonic force-velocity relationship (3), and both produce similar stall forces at the level of individual motor proteins and filaments. This means that both are capable of providing the force driving F-actin retrograde flow in the motor-clutch model, as previously suggested (37,38). Clutches are modeled as elastic springs (with stiffness κ_{clutch}) that can bind and unbind F-actin at rates k_{on} and k_{off}, respectively. k_{off} follows a force-dependent rate law described in Eq. S1 and (6) that also depends on the characteristic bond rupture force F_{bond}. Bound clutches transmit forces to the substrate, which is modeled as a linear spring with stiffness κ_{sub}. The value of κ_{sub} was set to 1000 pN nm^{-1} to reflect the rigid modulus of elastomer materials (elastic modulus, E = ~1000 kPa) used to make microchannel devices (39).

Linear springs representing the nucleo-cytoskeletal compliance (κ_{cell}) connect modules to the central cell body, which contains an ensemble of clutches ($n_{clutch,cell}$) that follow the same binding and unbinding rules as module clutches. F-actin assembly fuels the module elongation velocity (v_{actin}), distributing A_{total} between modules and a soluble pool (A_G). New modules are nucleated at a rate (k_{nuc}) that scales with A_G by a power-law relationship (9), described in Eq. S8. Modules are capped at a first-order capping rate (k_{cap}). Capped modules no longer extend by actin polymerization but still shorten by retrograde flow until they are destroyed after passing a minimal threshold length (L_{min}). Mass balances on n_{motor}, n_{clutch}, and A_{total} govern their distribution between modules and the cell body. In total, the 1D CMS contains 18 parameters for the cell, eight of which define the motor-clutch system properties (40), plus a variable substrate stiffness (Table S1). The values of these parameters and the constraints on their relationships have been previously described by both our previous work and that of several other labs (6,7,9,40–44).

In the previous 2D CMS (9), new modules were generated at a random angle in the Cartesian *x*, *y* plane (i.e., between 0 and 2π radians). Initially, the 1D CMS assigned modules a random binary orientation along the $\pm x$ direction (i.e., 0 or π radians) with equal probability of nucleating new modules in either orientation. Multiple modules overlapping in one direction is permitted because cells can extend multiple modules in a similar vector direction, such as along parallel-aligned fibers (12). Simulated trajectories obtained from sampling the cell body position (x_{cell}) at 5 min intervals (Fig. 1 *B*) yielded approximately linear MSD versus time curves (Fig. 1 *C*), consistent with a 1D random walk (35). This is expected given that earlier versions of the CMS predict a 2D random walk (9). By contrast, previous studies of cell migration in confined microfluidic channels suggest that cells often follow persistent ballistic trajectories (29,32).

To test whether the 1D CMS could produce ballistic trajectories, we added a variable polarity factor ψ_{pol} to simulations. As in previous versions of the CMS, module nucleation is a possible event at each simulation step that occurs at a G-actin-dependent rate k_{nuc} (see Eq. S8). The value of ψ_{pol} is defined between 0 and 1 and represents the probability that a newly nucleated module will be oriented in the $+x$ direction. The corresponding probability ($1 - \psi_{pol}$) thus gives the probability that a module is nucleated in the $-x$ direction. In other words, the probability that a new module will be nucleated pointing in the $+x$ direction follows a binomial distribution with parameters of ψ_{pol} and $\pm x$ as the possible outcomes (Fig. 1 *B*). Starting from the case in which nucleation probability is uniform ($\psi_{pol} = 0.5$), increasing ψ_{pol} consistently produced drift in the $+x$ direction for individual cell traces (Fig. 1 *B*), which yielded nonlinear (concave up) MSD versus time lag plots (Fig. 1 *C*). This behavior is consistent with previously described superdiffusive models of cell motility, including the persistent random walk (PRW) model that is often used to analyze directed cell migration (45,46). Intriguingly, changing the value of ψ_{pol} did not change the average number of protrusions (Fig. S1), confirming that persistent migration can be recapitulated in the 1D CMS as a function of directionally biased module nucleation and turnover.

Human glioma cells move persistently in confined microchannels

We next sought an engineered platform that would enable us to observe and track individual cells migrating within confined 1D channels and compare to simulation predictions. Using photolithography and PDMS replica-molding techniques (36), we created devices that featured 12-μm-wide channels with a height of 5 μm (60-μm^2 rectangular cross-sectional area) emerging from inlet ports (Fig. 2, *A* and *B*). U251 cells were labeled with a live-cell nucleus-tracking dye before seeding in devices. Seeded cells spontaneously migrated out of the inlet ports and migrated along the channel axis (Fig. 2 *C*). Time-lapse videos were acquired in the phase and DAPI channels every 5 min, and individual nuclei were tracked using a semiautomated script (11). Migrating cells elongated along the channel axis, generally forming long leading protrusions and smaller trailing ones (Fig. 2 *C*; Video S1), and moved away from the inlets toward the device exterior. The nucleus typically filled the lateral width of the channel and maintained a near-constant size and shape (Fig. S2).

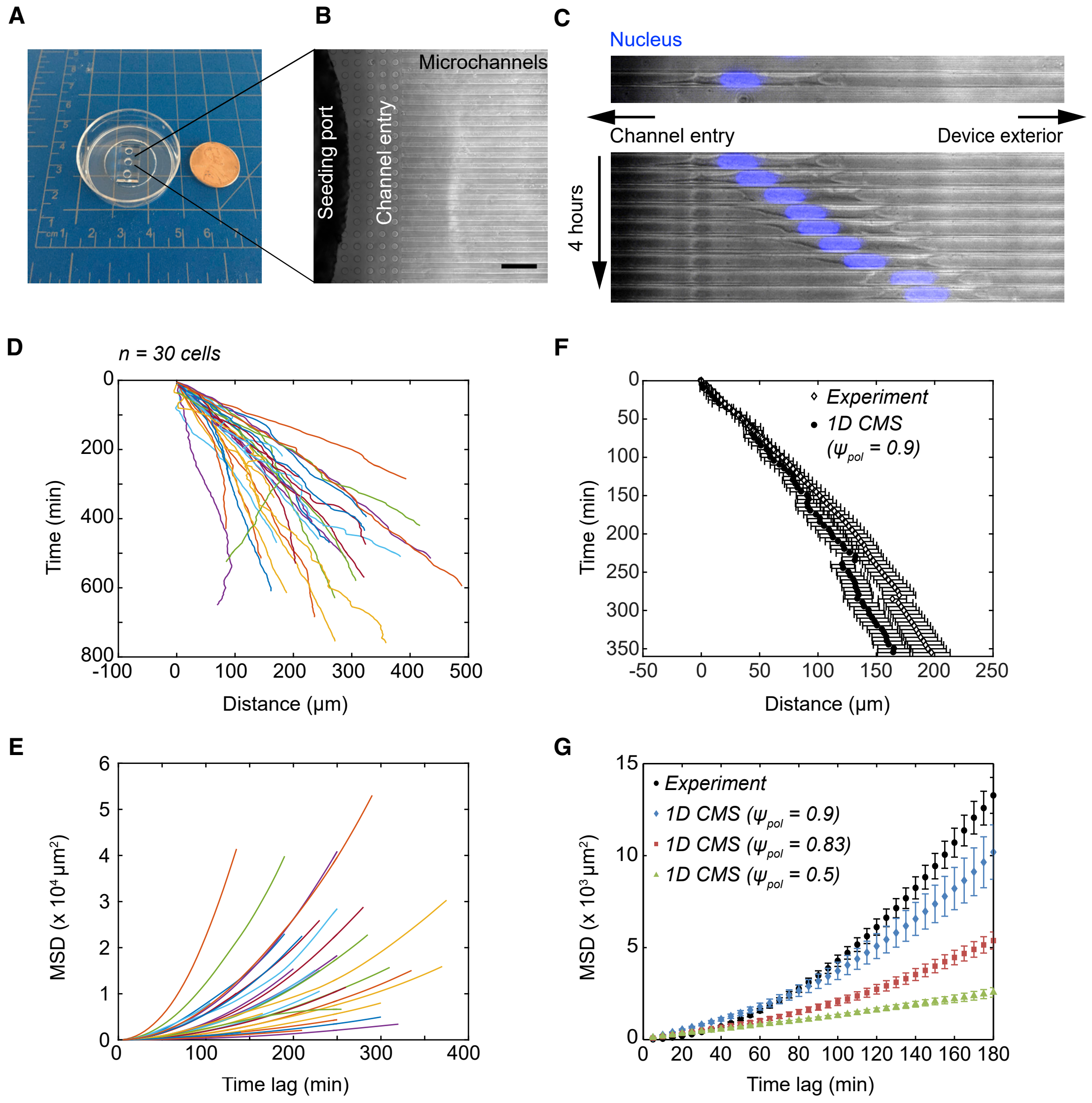

FIGURE 2 Tracking individual glioma cell nuclei in channels reveals persistent migration behaviors. (*A*) A photograph of an assembled device bonded to a 35-mm glass-bottom dish is shown. Note the channels extending from inlet seeding ports drilled into the PDMS block. A US one-cent coin is shown for scale, and the grid spacing is 1 cm. (*B*) An image of a drilled inlet port in an assembled device showing the entry chamber and 12-μm-wide channels (a height of 5 μm), acquired at 10× magnification using phase contrast optics, is given. Scale bars, 100 μm. (*C*) (*Top*) U251 human glioma cell migrating within a confined 12-μm-wide channel imaged using phase contrast and fluorescence is shown. Nucleus counterstain is shown in blue. Images were acquired at 20× magnification. Scale bars, 50 μm. (*Bottom*) A time-lapse sequence of images acquired for the cell in (*B*) is given. The images represent 4 h of total time displayed at 30-min intervals. (*D*) The nucleus x-position versus time as measured for $n = 30$ individual cells from a representative experiment is shown. Coordinates are plotted relative to the initial tracking position for each cell such that $x(\tau = 0) = 0$. For display purposes, coordinates of cells moving in the $-$x direction (*right* to *left*) were reversed. (*E*) The mean MSD versus time lag for the individual cells in (*E*) is shown. For display purposes, error bars are not shown. (*F*) The mean displacement versus time for a representative experiment (*filled circles*, mean ± SEM in (*D*)) and 1D CMS (*open diamonds*, mean ± SEM for $n = 36$ simulations with $\psi_{pol} = 0.9$ in Fig. 1 *B*) is shown. (*G*) The mean MSD versus time lag for a pooled control data set (*black circles*, $n =$ 403 cells from 12 independent experiments) and 1D CMS with $\psi_{pol} = 0.9$ (*blue diamonds*, $n = 60$ simulations), $\psi_{pol} = 0.83$ (*red squares*, $n = 36$ simulations), or $\psi_{pol} = 0.5$ (*green triangles*, $n = 40$ simulations) is shown. The error bars are mean ± SEM.

Individual cell trajectories exhibited variability ($n = 30$ cells from an example experiment in Fig. 2 *D*), but most cells moved persistently in one direction (away from the inlet port). Few cells exhibited saltatory motion as observed for glioma cells migrating in ex vivo brain slice cultures (11,14), and complete directional reversals were rare. Individual MSD versus time lag plots for experiments (Fig. 2 *E*) were typically nonlinear (concave up), consistent with superdiffusive or quasiballistic cell migration models (12,45,46). Mean displacement and MSD were also consistent with the 1D CMS predictions ($\psi_{pol} = 0.9$; Fig. 2, *F* and *G*). Similar simulated cell behaviors were observed when the substrate spring constant was increased to $\kappa_{sub} =$ 10^6 pN nm^{-1} (Fig. S3), suggesting that the 1D CMS predicts

a similar behavior for cells adhering to the PDMS device walls and glass bottom.

A diffusion-convection model describes 1D confined cell migration, whereas a PRW model yields unrealistic fitting parameters

Directed cell migration behaviors are often analyzed using a PRW model (12,46). The PRW model relates MSD ($\langle r^2(t) \rangle$) for a given time lag (t) to two fitting parameters: cell speed (S) and characteristic persistence time (P).

$$\langle r^2(t) \rangle = nS^2P^2\left(e^{\frac{-t}{P}} + \frac{t}{P} - 1\right). \tag{1}$$

Fitting a pooled control data set ($n = 403$ cells from 12 independent experiments) to Eq. 1, we obtained a mean speed ($S = 0.74 \pm 0.05$ μm min^{-1}; Fig. S4) that was comparable to other tumor cell lines (25,31,33) and stem cells (24) but much slower than immune cells (22,29,36) in channels of similar size. Mean persistence times measured from individual cell fits to Eq. 1 (1258 min; Fig. S4) exceeded the maximal imaging window duration (t_{exp} = 18 h = 1080 min). In contrast, glioma cells migrating along suspended polystyrene fibers have persistence times in the range of ~100 min, well within the bounds of typical microscopy experiments (12). Caution should thus be exercised in interpreting results obtained with a PRW model to avoid overfitting parameters.

Diffusion-convection or diffusion-drift models are also applied to study molecular-cellular scale motion, such as biopolymer filament assembly (47,48). Equation 2 relates MSD to a linear term (μ) that is analogous to a diffusion or motility coefficient and a quadratic term (v) representing drift velocity.

$$\langle r^2(t) \rangle = 2n\mu t + v^2t^2. \tag{2}$$

Fitting individual cell trajectories, Eq. 2 revealed cell-to-cell variability in both the motility coefficient (Fig. 3 *A*) and velocity (Fig. 3 *B*). A mean velocity of $v_{exp} = 0.51 \pm 0.02$ μm min^{-1} (or $v_{exp} = 8.5 \pm 0.3$ nm s^{-1}) agrees with the speeds obtained using the PRW model (Fig. S4).

Analyzing CMS results using Eq. 2 revealed very little difference in the motility coefficient between the three values of ψ_{pol}, whereas the velocity increased for larger values of ψ_{pol} (Fig. S1), corresponding to increasingly persistent migration. Comparing the most similar conditions, the overall mean motility coefficient was larger for simulations ($\psi_{pol} = 0.9$) than experiments ($\mu_{exp} = 1.61 \pm 0.14$ μm^2 min^{-1} vs. $\mu_{sim} = 7.27 \pm 0.80$ μm^2 min^{-1}). This discrepancy is likely due to the short-timescale reversals in cell position that were only seen in simulations (Fig. 1 *B*) because the diffusion term would be more sensitive to noise at short time lags. Simulated cell velocities

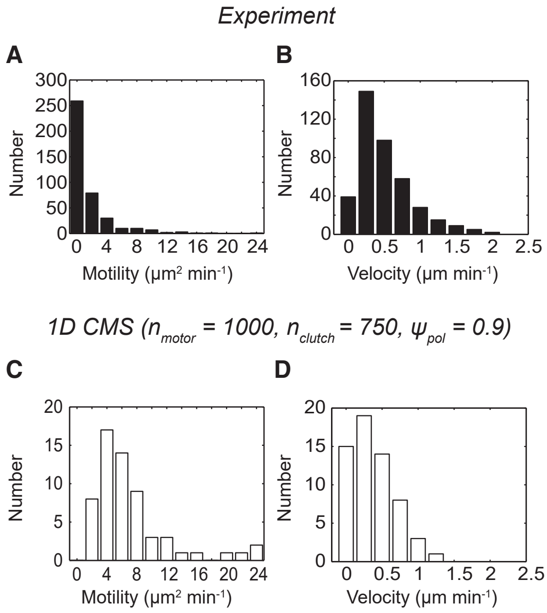

FIGURE 3 Estimates of motility coefficient and velocity from experimental and simulated data. (*A*) The histogram of the motility coefficients obtained for $n = 403$ cells, $\mu_{exp} = 1.61 \pm 0.14$ μm^2 min^{-1} (mean $\pm$ standard error (SE)), is shown. (*B*) The histogram of the velocities obtained for the cells in (*A*), $v_{exp} = 0.51 \pm 0.02$ μm min^{-1} (mean $\pm$ SE), is shown. (*C*) The histogram of the motility coefficients obtained for $n = 60$ individual simulated trajectories with $\psi_{pol} = 0.9$, $\mu_{sim} = 7.27 \pm 0.80$ μm^2 min^{-1} (mean $\pm$ SE) is shown. (*D*) The histogram of the velocities obtained for $n = 60$ individual simulated trajectories with $\psi_{pol} = 0.9$, $v_{sim} = 0.37 \pm 0.04$ μm min^{-1} (mean $\pm$ SE) is shown. Individual motility coefficients and velocities were obtained from fits to Eq. 2. All simulations were run with $n_{motor} = 1000$, $n_{clutch} = 750$, and $\psi_{pol} = 0.9$, and all other parameter values reported in Table S1.

were similar to experiments ($v_{exp} = 0.51 \pm 0.02$ μm min^{-1} vs. $v_{sim} = 0.37 \pm 0.04$ μm min^{-1}), explaining the consistency between the experiment and simulation (Fig. 2 *G*), because the quadratic velocity term dominates the behavior of Eq. 2 for longer time lags.

CMS predicts the effects of integrin-mediated adhesion and myosin II inhibition on confined glioma cell migration

Motor-clutch-based cell migration on 2D hydrogel substrates involves integrin clutches and myosin II motor activity (9), but it is unclear what roles these components play in confined environments. In particular, reducing adhesiveness (achieved by either reducing receptor expression or ligand availability) is theoretically predicted to reduce motility (4,11) but is experimentally shown to increase motility in vitro for some cell types in confinement (18). Earlier studies using the CMS recapitulate a biphasic relationship between adhesiveness and motility (11) in which cells achieve maximal motility at intermediate adhesiveness. We therefore sought to test whether the 1D CMS would produce similar results by reducing the value of n_{clutch}. Starting

with the base parameter set (n_{motor} = 1000, n_{clutch} = 750, ψ_{pol} = 0.9; Table S1), independently reducing n_{clutch} produced a biphasic trend in both the motility coefficient (Fig. 4 *A*) and the velocity (Fig. 4 *B*). Consistent with 2D simulations, the largest value of either quantity was obtained at n_{clutch} = 75 or a 10-fold reduction from the base parameter value (Table S1).

As an experimental test of the model, we treated U251 cells in microchannels with cyclo-(Arg-Gly-Asp) peptide (cRGD), a competitive inhibitor of $\alpha_v\beta_3$ integrin-fibronectin interactions (49). On 2D hydrogel substrates, cRGD reduces traction force and migration, consistent with 2D CMS predictions (9). Cells exposed to 0.1–1 μM cRGD demonstrated biphasic trends in the motility coefficient (Fig. 4 *C*) and velocity (Fig. 4 *D*), with the largest values observed at 0.1–0.3 μM. U251 cells thus rely on integrin clutches in confined microchannels, and their migration behavior is consistent with a biphasic adhesiveness relationship (4,11).

Myosin II inhibition slows glioma cell migration on 2D hydrogels (9) and stalls cell migration in an ex vivo brain slice culture (14,50). Independently reducing simulated myosin II motor number (n_{motor}) from its base value (n_{motor} = 1000; Table S1) nearly monotonically reduced the motility coefficient (Fig. 5 *A*) and velocity (Fig. 5 *B*). Our imaging approaches are not compatible with the myosin II inhibitor blebbistatin because imaging at near-ultraviolet wavelengths causes drug inactivation and cytotoxicity (51). Rho-associated kinase (ROCK) is one of the major pathways for myosin II activation in U251 cells (14), so as an orthogonal approach, we inhibited this pathway using 15 μM Y-27632. Compared to controls, Y-27632 had little effect on the motility coefficient (Fig. 5 *C*) but significantly reduced the velocity (Fig. 5 *D*). This reduction in velocity can be explained by reducing n_{motor} in the 1D CMS, suggesting that U251 cells use ROCK-mediated myosin II force generation in confinement. In other studies, confined cells were insensitive to ROCK inhibitors (24,25,52), suggesting either 1) myosin II activation is controlled independently of the ROCK pathway in these cells or 2) other force generation mechanisms such as actin polymerization or osmotic pressure gradients (23) are the dominant means of force generation.

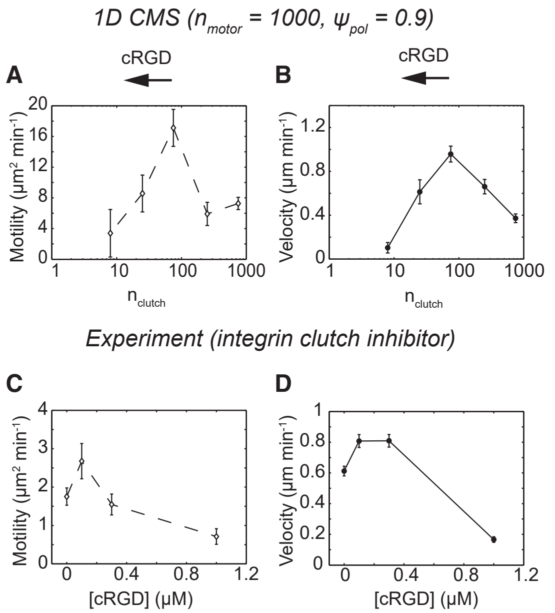

FIGURE 4 Biphasic relationship between velocity and integrin clutch number for simulations and confined glioma cells. (*A*) The motility coefficients from simulations in which n_{clutch} was varied independently of other parameters are shown (n_{clutch} = 8, 25, 75, 250, and 750 and n = 8, 8, 16, 16, and 60 simulated cells). All simulations were run with n_{motor} = 1000 and ψ_{pol} = 0.9, and all other parameter values were reported in Table S1. (*B*) The velocities from the simulation conditions in (*A*) are shown. (*C*) The motility coefficients for U251 glioma cells treated with complete media (control) or 0.1, 0.3, or 1 μM cRGD (n $\geq$ 72 cells) are given. (*D*) The velocities from the experimental conditions in (*C*) are shown. Individual motility coefficients and velocities were obtained from fits to Eq. 2. The error bars represent mean $\pm$ SEM. Pairwise statistics are reported in Table S2.

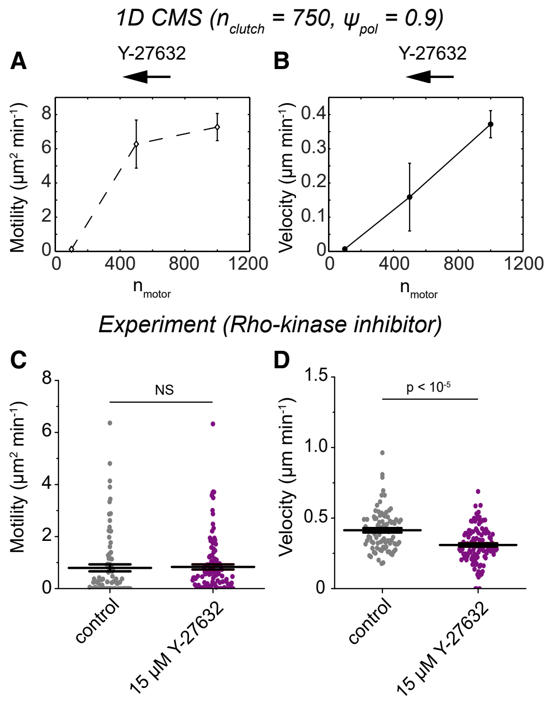

FIGURE 5 Monotonic relationship between velocity and myosin II motor number for simulations and confined glioma cells. (*A*) The motility coefficients from simulations in which n_{motor} was varied independently of other parameters are shown (n_{motor} = 100, 500, and 1000 and n = 8, 8, and 60 simulated cells). All simulations were run with n_{clutch} = 750 and ψ_{pol} = 0.9, and all other parameter values were reported in Table S1. (*B*) The velocities from the simulation conditions in (*A*) are shown. (*C*) The motility coefficients for U251 glioma cells treated with complete media (control) or 15 μM Y-27632 (n = 87, 112 cells) are given.(*D*) The velocities from the experimental conditions in (*C*) are shown. Individual motility coefficients and velocities were obtained from fits to Eq. 2. The error bars represent mean $\pm$ SEM, NS denotes no significant difference, and $p > 0.01$ by one-way Kruskal-Wallis ANOVA. Pairwise statistics for (*A* and *B*) are reported in Table S2. To see this figure in color, go online.

Actin polymerization and dynamic microtubules are required for confined glioma migration

Actin polymerization is critical for cell migration on unconfined 2D substrates, but its role in confined migration is controversial (23–25). Notably, confined migration driven by osmotic pressures is insensitive to latrunculin A (LatA), a potent inhibitor of actin polymerization (23). We tested the actin-dependence of confined U251 cell migration by treating cells with either vehicle controls (dimethyl sulfoxide (DMSO)) or varying doses of LatA. Treating cells with 50 nM LatA slowed movement, whereas 500 nM LatA nearly completely stalled motion (Video S2). Tracking cells and quantifying results using Eq. 2 revealed corresponding dose-dependent decreases in the motility coefficient (Fig. 6 *A*) and velocity (Fig. 6 *B*).

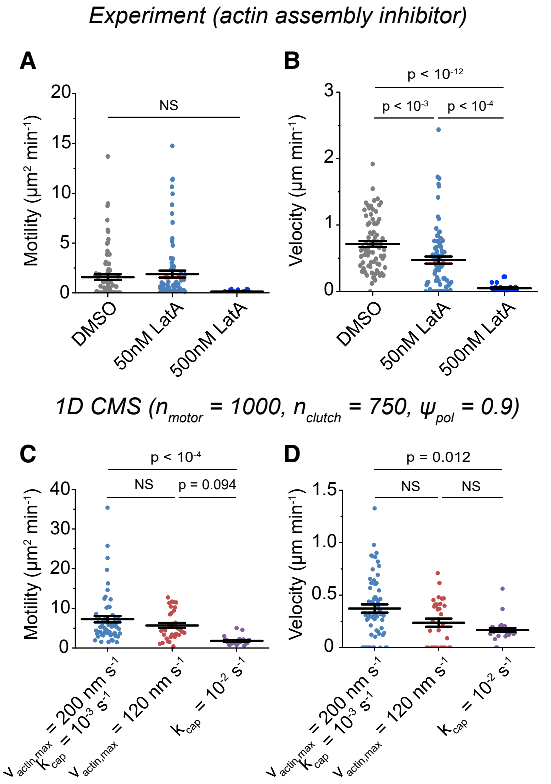

FIGURE 6 Simulated and experimental predictions of the effects of actin polymerization inhibitors on confined glioma cell migration. (*A*) The motility coefficients for U251 cells expressing eGFP-β-actin and treated with vehicle control (DMSO) or 50 or 500 nM LatA are shown ($n = 78, 81, 23$ cells). (*B*) The velocities for the experimental conditions in (*A*) are shown. (*C*) The motility coefficients from simulations using a reference parameter set (Table S1; $v_{actin,max} = 200$ nm s^{-1}, $k_{cap} = 0.001$ s^{-1}) or simulations in which $v_{actin,max}$ was reduced ($v_{actin,max} = 120$ nm s^{-1}) or in which k_{cap} was increased ($k_{cap} = 0.01$ s^{-1}) are shown ($n = 60$, 28, and 24 simulations). All simulations had $n_{motor} = 1000$, $n_{clutch} = 750$, and $\psi_{pol} = 0.9$, and all other parameter values are reported in Table S1. (*D*) The velocities from the simulations in (*C*) are shown. Individual motility coefficients and velocities were obtained from fits to Eq. 2. The error bars represent mean ± SEM, NS denotes no significant difference, and $p > 0.01$ by one-way Kruskal-Wallis ANOVA. To see this figure in color, go online.

Actin polymerization drives protrusion extension in the 1D CMS and scales a maximal polymerization rate from its base value ($v_{actin,max} = 200$ nm s^{-1}; Table S1). Reducing the maximum actin polymerization rate ($v_{actin,max} = 120$ nm s^{-1}) impairs motility on 2D substrates (10), and the same parameter value change in the 1D CMS also reduced the cell motility coefficient (Fig. 6 *C*) and velocity (Fig. 6 *D*). As an alternative mechanism, the 1D CMS features a stochastic capping rate for modules ($k_{cap} = 10^{-3}$ s^{-1}; Table S1) as a mechanism to facilitate module turnover. Because LatA prevents actin subunits from binding to F-actin barbed ends (53), we tested the possibility that increased module capping would produce similar results. Increasing k_{cap} by an order of magnitude ($k_{cap} = 10^{-2}$ s^{-1}) reduced the simulated motility coefficient (Fig. 6 *C*) and velocity (Fig. 6 *D*), similar to decreasing $v_{actin,max}$ but contrasting with our earlier results on 2D substrates (10). Actin protrusion and turnover rates thus regulate migration speed in the 1D CMS, consistent with experimental measurements of cells treated with LatA.

Dynamic microtubules establish and maintain the polarity of migrating cells (54), whereas microtubule-targeting agents (MTAs) disrupt microtubule-dependent polarity and directed migration (10). MTAs are widely used in chemotherapy and are distinguished by their effects on polymer assembly: 1) assembly promoters such as paclitaxel (PTX) and 2) disassembly promoters such as vinblastine (VBL) (55). MTAs' most pronounced effect is kinetic stabilization, which reduces the accumulation of microtubule tip-tracking proteins such as EB1 to microtubule ends and is a common effect of both assembly-promoting and disassembly-promoting MTAs (55). We confirmed that U251 cells expressing EB1-eGFP had dynamic microtubules (Fig. S5), and microtubules exhibited growth speeds similar to earlier measurements in U251 cells on 2D unconfined substrates (10). Tracking U251 cells treated with either PTX or VBL (at 100 nM) revealed that both MTAs had little effect on motility coefficient (Fig. 7 *A*), but each significantly reduced velocity (Fig. 7 *B*) compared to vehicle (DMSO) controls. Interestingly, neither PTX nor VBL significantly affected either the cell length or nucleus position in the channels (Fig. S5), suggesting that MTAs do not significantly disrupt nucleus positioning or cell polarity and may influence cell migration through other means.

Balzer et al. (25) observed that EB1-labeled microtubule arrival at the leading edge was concomitant with leading edge protrusion in confinement, suggesting a direct correlation between microtubule impact and forward cell protrusion. We have also previously suggested that MTAs reduce maximal protrusion velocity ($v_{actin,max}$) to impair migration in 2D (10), and our earlier 1D CMS results in which $v_{actin,max}$ was reduced (Fig. 6, *A* and *B*) are in line with both of these observations. Alternatively, our earlier study (10) suggested that microtubules can influence the basal nucleation rate for new modules ($k_{nuc,0} = 1$ s^{-1}; Table S1). Namely, these

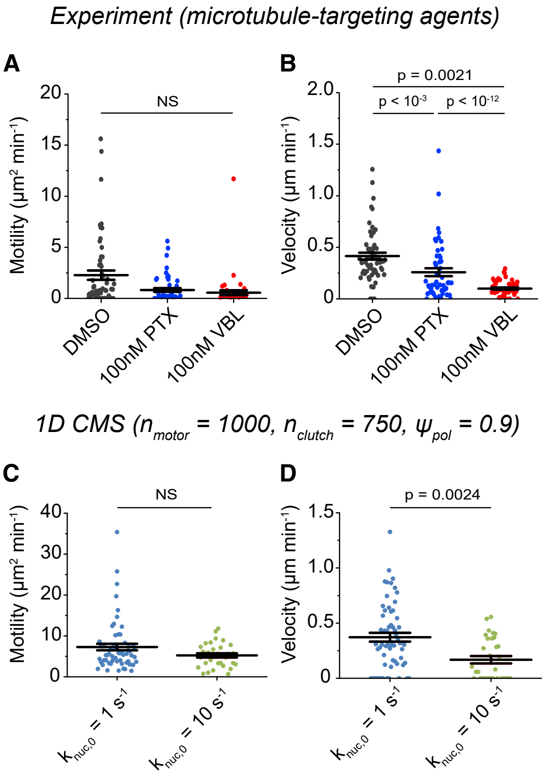

FIGURE 7 Simulated and experimental predictions of the effects of MTAs on confined glioma cell migration. (*A*) The motility coefficients for U251 cells treated with vehicle control (DMSO), 100 nM PTX, or 100 nM VBL ($n = 58$, 52, and 50 cells) are shown. (*B*) The velocities from the experiments in (*A*) are shown. (*C*) The motility coefficients from simulations using a reference parameter set (Table S1; $k_{nuc,0} = 1\ s^{-1}$) or in which $k_{nuc,0}$ was increased ($k_{nuc,0} = 10\ s^{-1}$) are shown ($n = 60$, 28 simulations). All simulations had $n_{motor} = 1000$, $n_{clutch} = 750$, and $\psi_{pol} = 0.9$, and all other parameter values were reported in Table S1. (*D*). The velocities from the simulations in (*C*) are shown. Individual motility coefficients and velocities were obtained from fits to Eq. 2. The error bars represent mean ± SEM, NS denotes no significant difference, and $p > 0.01$ by Kruskal-Wallis one-way ANOVA. To see this figure in color, go online.

results suggest that increasing the number of protrusions slows motility in 2D, consistent with experimental MTA effects (10). Increasing the basal nucleation rate for new modules ($k_{nuc,0} = 10\ s^{-1}$) reduced the simulated cell motility coefficient (Fig. 7 *C*) and velocity (Fig. 7 *D*), suggesting that MTAs inhibit glioma cell migration by similar mechanisms on 2D substrates and in 1D confinement. We conclude that the 1D CMS can accurately predict mechanistic behaviors of MTAs and actin assembly inhibitors and that glioma cell migration in confinement relies upon dynamic cytoskeletal self-assembly.

DISCUSSION

Cells experience mechanical confinement while invading dense tissues in vivo. Similar confinement can be reliably reproduced in PDMS microchannel assays, and live-cell-tracking measurements can be compared to biophysical simulations of cell migration mechanics. Using simulation parameters calibrated to previous 2D measurements of human glioma cells as a starting point, we reproduced mean cell migration speeds close to ~0.5 μm per min^{-1}, consistent with experiments. This corresponds to a tumor growth rate of 25 cm per year^{-1}, within the range of tumor growth rates measured in the clinic (56). The 1D CMS also predicts the effects of pharmacological agents targeting components of the motor-clutch system, including a biphasic relationship between substrate adhesiveness and speed. These results suggest that glioma cells (and potentially other cell types) employ a motor-clutch force transmission mechanism to migrate within confined spaces.

In vivo, glioma cells likely employ CD44 clutches when engaging with the hyaluronic-acid-rich stroma (11). Integrins are also likely involved as cells interact with basement membranes that contain collagen and laminin (17). Invading cells could also employ friction-based amoeboid migration in environments with low ligand density. Frictional forces could be incorporated in the motor-clutch model by reducing the characteristic clutch bond force (F_{bond}) and increasing the binding and unbinding rates (k_{on} and k_{off}, respectively). Confinement appears to be required for adhesion-independent migration because the same cells move slowly in unconfined environments lacking adhesion molecules (18). To simulate this, the clutch number (n_{clutch}) could be increased proportionally to the increase in contact area, increasing cell speed as they enter the "optimal" adhesion regime.

F-actin forms the basis of cellular protrusions in many cells, and the 1D CMS captures the dynamics of actin assembly and disassembly within modules. F-actin in modules is under tension between motor-based pulling forces and substrate deformation, so the model considers it to be rigid in accordance with the observed strain stiffening behavior of stress fibers and cross-linked actin gels (57). Furthermore, we consider F-actin to behave elastically because clutch binding and unbinding events occur on the ~10^{-1}–10^{-3} s timescale, which is faster than typical intracellular cross-linker lifetimes that give rise to viscoelastic behaviors within F-actin networks (57). Other physical models explicitly model F-actin networks as a viscoelastic fluid, accounting for energy dissipation through cross-linker binding and unbinding (26,58). Although this version of the 1D CMS does not include a viscoelastic term for the cytoskeleton, future works could address this by including time-varying mechanical elements within the cell body instead of the current elastic cell spring term (κ_{cell}).

Osmotic-pressure-driven migration of tumor cells has previously been proposed as a mechanism for glioma dispersion (16). In osmotic-pressure-driven models of cell migration (23), asymmetrically distributed ion pumps at the leading and trailing edge create a net protrusive force on the forward-facing cell membrane, whereas friction between the flowing cortex and channel wall transmits forces. In this context, migrating cells are insensitive to actin

polymerization inhibitors (such as LatA), suggesting that the osmotic engine supplants actin-based migration when hydraulic resistance would otherwise stall actin polymerization (26). Contrasting with this result, we conclude that hydrodynamic drag forces are minimal in our system (see Supporting Materials and Methods) and find that LatA stalled U251 cell migration in these channels (Fig. 6, *A* and *B*; Video S2). There are several nonexclusive explanations for these contrasting observations. First, the channels in (23) have a smaller cross-sectional area (30 μm^2 compared to 60 μm^2 in this study), suggesting that hydrodynamic drag forces are smaller in our system. Using the mean velocity that we experimentally measured for U251 cells (Fig. 3 *B*), fluid drag forces on individual cells in our channels are on the order of ~6.5 pN (see calculation in Supporting Materials and Methods). This is orders of magnitude smaller than the total stall force in simulations (Table S1) or traction forces produced by adherent cells (30,37), suggesting that U251 cells can readily overcome hydrodynamic drag. Deformation of larger organelles, such as the nucleus, may effectively increase drag forces in narrower channels as well. Second, the osmotic engine appears to require particular ion transport and water flux proteins (23) that may vary in expression between cell lines and contribute to differential sensitivity to hydrodynamic drag. Third, some cells can internalize fluid through macropinocytosis to minimize hydrodynamic drag (59).

The 1D CMS includes a polarity parameter (ψ_{pol}) to represent a directional bias along the channel axis. Empirically, we found that $\psi_{pol} = 0.9$ produced reasonably close fits to experimental data (see Fig. 2, *F* and *G*) and used this as our base value for the U251 parameter set in simulations (Table S1). Although we note that the constant polarity value may be a simplification of the underlying biological mechanism, removing the directional bias in polarity ($\psi_{pol} = 0.5$) yielded diffusive, nonpersistent simulated cell migration that was not observed experimentally (Fig. 1, *B* and *C*). Several examples of asymmetric cytoskeletal regulation exist in the literature that may point to a biological mechanism. Confinement polarizes the distribution of F-actin stress fibers and phosphorylated myosin II light chain in migrating glioma cells, which, in turn, polarize force generation along the channel axis (60). Microtubules are also involved in polarized cell migration in confined channels (25,32), where they may influence the activity of signaling proteins that regulate F-actin dynamics, such as Rho-family GTPases (54). A recent study examined the effect of microtubule-based delivery of Rho guanine nucleotide exchange factor (GEF) H1 on the dynamics of cellular protrusions (61). Asymmetric delivery of Rho GEF H1 by microtubules could consistently activate Rho GTPase at the leading edge of cells, thus driving asymmetric protrusion nucleation and polarized migration. We note that MTAs slowed U251 migration in channels (Fig. 7, *A* and *B*), although future work will be required to identify the signaling factors involved in this response. Regardless of the mechanism, recapitulating the behavior of cells in the channels is sensitive to the value of ψ_{pol}, an effect that is not required for simulations of cells on 2D substrates using the same physical model of migration (9). This confinement-induced polarity may play a role in tumor progression by biasing cell movements away from the tumor bulk and into healthy tissue, as individual glioma cells are often observed migrating away from tumors in ex vivo slice cultures (11,13,14).

Photolithography and PDMS replica molding enabled production of micrometer-scale channels for parallel analysis of a large number of individual cell trajectories (~1000 cells in a single study). One disadvantage of PDMS molding techniques is that devices are often made from materials with a high elastic modulus (E = ~1000 kPa) and bound to glass dishes (E = ~10^7 kPa). These values are above the limit of stiffness sensitivity for U251 cells (9,40) and prohibit the measurement of traction forces, although we note that certain PDMS mixtures can yield significantly softer moduli (39). Pathak and Kumar (60) used photolithography to manufacture confined channels in polyacrylamide hydrogels, enabling them to independently control the channel width (w = 10–40 μm) and device stiffness (E = 0.4–120 kPa). Interestingly, they reported biphasic cell speed as a function of hydrogel modulus, consistent with CMS predictions (9). However, the channels produced with this method only laterally confined the cell, and they did not produce any channel structures with dimensions <10 μm, which are easily produced by PDMS replica molding.

Previous studies have inferred relationships between biophysical measurements in 2D substrates and confined migration behaviors (30,31) but did not employ simulated migration to test those predictions. In this study, we used motor-clutch parameters for glioma cells measured on 2D unconfined substrates (9,10) to predict confined migration behaviors. Altogether, our results suggest that the CMS can predict confined tumor cell migration, as well as antimotility therapy, using extant cell migration data. Future work could connect these individual cell behaviors to tumor-scale mathematical models (62,63), which could, in turn, provide inputs for multiscale models of tissue invasion and avenues for therapeutic intervention.

SUPPORTING MATERIAL

Supporting Material can be found online at https://doi.org/10.1016/j.bpj.2020.01.048.

AUTHOR CONTRIBUTIONS

Conceptualization, L.S.P., P.V., M.P., and D.J.O.; methodology, all authors; software, L.S.P.; validation, L.S.P. and M.R.S.; formal analysis, L.S.P., M.R.S., and D.J.O.; resources, P.V., M.P., and D.J.O.; writing—original draft, L.S.P. and D.J.O.; writing—review and editing, all authors; visualization, L.S.P.; supervision, P.V., M.P., and D.J.O.; and funding acquisition, L.S.P., M.P., and D.J.O.

ACKNOWLEDGMENTS

We thank the members of the Odde and Piel laboratories for helpful discussions and M. Titus and C. Cadart for comments on the manuscript drafts. We thank R. Attia and M. Fisher for help with photomask design and G. Doak, J. Valdez, X. Lu, and D. Wood for their assistance with photolithography. We thank A. -M. Lennon-Duménil for use of laboratory equipment and the Bioimaging Cell and Tissue Core Facility at Institut Curie for use of microscopes during initial data collection. We thank the Minnesota Supercomputing Institute for use of the high-performance computing resources.

This work is supported by the National Science Foundation (NSF) through the National Nano Coordinated Infrastructure network under award number ECCS-1542202 (to the Minnesota Nano Center). L.S.P. was supported by a 3M Science & Technology Fellowship and an NSF Graduate Research Fellowship (0039202). M.R.S. was supported by the Undergraduate Research Opportunities Program through the University of Minnesota. Other funding support included an NSF Graduate Research Opportunities Worldwide grant and an STEM Chateaubriand Fellowship to L.S.P., Association Nationale pour la Recherche grant ANR-16-CE13-0009 to P.V., European Research Council consolidator grant 311205 PROMICO to M.P., and National Institutes of Health grants U54 CA210190 and R01 CA172986 to D.J.O. P.V. is an INSERM investigator.

SUPPORTING CITATIONS

References (64–73) appear in the Supporting Material.

REFERENCES

1. Lefranc, F., J. Brotchi, and R. Kiss. 2005. Possible future issues in the treatment of glioblastomas: special emphasis on cell migration and the resistance of migrating glioblastoma cells to apoptosis. *J. Clin. Oncol.* 23:2411–2422.
2. Lauffenburger, D. A., and A. F. Horwitz. 1996. Cell migration: a physically integrated molecular process. *Cell.* 84:359–369.
3. Prahl, L. S., and D. J. Odde. 2018. Modeling cell migration mechanics. *Adv. Exp. Med. Biol.* 1092:159–187.
4. DiMilla, P. A., K. Barbee, and D. A. Lauffenburger. 1991. Mathematical model for the effects of adhesion and mechanics on cell migration speed. *Biophys. J.* 60:15–37.
5. Danuser, G., J. Allard, and A. Mogilner. 2013. Mathematical modeling of eukaryotic cell migration: insights beyond experiments. *Annu. Rev. Cell Dev. Biol.* 29:501–528.
6. Chan, C. E., and D. J. Odde. 2008. Traction dynamics of filopodia on compliant substrates. *Science.* 322:1687–1691.
7. Elosegui-Artola, A., R. Oria, ..., P. Roca-Cusachs. 2016. Mechanical regulation of a molecular clutch defines force transmission and transduction in response to matrix rigidity. *Nat. Cell Biol.* 18:540–548.
8. Elosegui-Artola, A., X. Trepat, and P. Roca-Cusachs. 2018. Control of mechanotransduction by molecular clutch dynamics. *Trends Cell Biol.* 28:356–367.
9. Bangasser, B. L., G. A. Shamsan, ..., D. J. Odde. 2017. Shifting the optimal stiffness for cell migration. *Nat. Commun.* 8:15313.
10. Prahl, L. S., P. F. Bangasser, ..., D. J. Odde. 2018. Microtubule-based control of motor-clutch system mechanics in glioma cell migration. *Cell Rep.* 25:2591–2604.e8.
11. Klank, R. L., S. A. Decker Grunke, ..., D. J. Odde. 2017. Biphasic dependence of glioma survival and cell migration on CD44 expression level. *Cell Rep.* 18:23–31.
12. Estabridis, H. M., A. Jana, ..., D. J. Odde. 2018. Cell migration in 1D and 2D nanofiber microenvironments. *Ann. Biomed. Eng.* 46:392–403.
13. Liu, C. J., G. A. Shamsan, ..., D. J. Odde. 2019. Glioma cell migration dynamics in brain tissue assessed by multimodal optical imaging. *Biophys. J.* 117:1179–1188.
14. Beadle, C., M. C. Assanah, ..., P. Canoll. 2008. The role of myosin II in glioma invasion of the brain. *Mol. Biol. Cell.* 19:3357–3368.
15. Novak, U., and A. H. Kaye. 2000. Extracellular matrix and the brain: components and function. *J. Clin. Neurosci.* 7:280–290.
16. Cuddapah, V. A., S. Robel, ..., H. Sontheimer. 2014. A neurocentric perspective on glioma invasion. *Nat. Rev. Neurosci.* 15:455–465.
17. Gritsenko, P. G., O. Ilina, and P. Friedl. 2012. Interstitial guidance of cancer invasion. *J. Pathol.* 226:185–199.
18. Liu, Y.-J., M. Le Berre, ..., M. Piel. 2015. Confinement and low adhesion induce fast amoeboid migration of slow mesenchymal cells. *Cell.* 160:659–672.
19. Bergert, M., A. Erzberger, ..., E. K. Paluch. 2015. Force transmission during adhesion-independent migration. *Nat. Cell Biol.* 17:524–529.
20. Ruprecht, V., S. Wieser, ..., C.-P. Heisenberg. 2015. Cortical contractility triggers a stochastic switch to fast amoeboid cell motility. *Cell.* 160:673–685.
21. Davidson, P. M., C. Denais, ..., J. Lammerding. 2014. Nuclear deformability constitutes a rate-limiting step during cell migration in 3-D environments. *Cell. Mol. Bioeng.* 7:293–306.
22. Thiam, H.-R., P. Vargas, ..., M. Piel. 2016. Perinuclear Arp2/3-driven actin polymerization enables nuclear deformation to facilitate cell migration through complex environments. *Nat. Commun.* 7:10997.
23. Stroka, K. M., H. Jiang, ..., K. Konstantopoulos. 2014. Water permeation drives tumor cell migration in confined microenvironments. *Cell.* 157:611–623.
24. Doolin, M. T., and K. M. Stroka. 2018. Physical confinement alters cytoskeletal contributions towards human mesenchymal stem cell migration. *Cytoskeleton (Hoboken).* 75:103–117.
25. Balzer, E. M., Z. Tong, ..., K. Konstantopoulos. 2012. Physical confinement alters tumor cell adhesion and migration phenotypes. *FASEB J.* 26:4045–4056.
26. Li, Y., and S. X. Sun. 2018. Transition from actin-driven to water-driven cell migration depends on external hydraulic resistance. *Biophys. J.* 114:2965–2973.
27. Monzo, P., Y. K. Chong, ..., M. P. Sheetz. 2016. Mechanical confinement triggers glioma linear migration dependent on formin FHOD3. *Mol. Biol. Cell.* 27:1246–1261.
28. Smith, C. L., O. Kilic, ..., A. Levchenko. 2016. Migration phenotype of brain-cancer cells predicts patient outcomes. *Cell Rep.* 15:2616–2624.
29. Vargas, P., P. Maiuri, ..., A. M. Lennon-Duménil. 2016. Innate control of actin nucleation determines two distinct migration behaviours in dendritic cells. *Nat. Cell Biol.* 18:43–53.
30. Mekhdjian, A. H., F. Kai, ..., V. M. Weaver. 2017. Integrin-mediated traction force enhances paxillin molecular associations and adhesion dynamics that increase the invasiveness of tumor cells into a three-dimensional extracellular matrix. *Mol. Biol. Cell.* 28:1467–1488.
31. Lautscham, L. A., C. Kämmerer, ..., B. Fabry. 2015. Migration in confined 3D environments is determined by a combination of adhesiveness, nuclear volume, contractility, and cell stiffness. *Biophys. J.* 109:900–913.
32. Prentice-Mott, H. V., Y. Meroz, ..., J. V. Shah. 2016. Directional memory arises from long-lived cytoskeletal asymmetries in polarized chemotactic cells. *Proc. Natl. Acad. Sci. USA.* 113:1267–1272.
33. Irimia, D., and M. Toner. 2009. Spontaneous migration of cancer cells under conditions of mechanical confinement. *Integr. Biol.* 1:506–512.
34. Wilson, K., A. Lewalle, ..., G. Charras. 2013. Mechanisms of leading edge protrusion in interstitial migration. *Nat. Commun.* 4:2896.
35. Dickinson, R. B., and R. T. Tranquillo. 1993. Optimal estimation of cell movement indices from the statistical analysis of cell tracking data. *AIChE J.* 39:1995–2010.
36. Heuzé, M. L., O. Collin, ..., M. Piel. 2011. Cell migration in confinement: a micro-channel-based assay. *In* Cell Migration: Developmental

Methods and Protocols. C. M. Wells and M. Parsons, eds. Humana Press, pp. 415–434.

37. Gardel, M. L., B. Sabass, …, C. M. Waterman. 2008. Traction stress in focal adhesions correlates biphasically with actin retrograde flow speed. *J. Cell Biol.* 183:999–1005.
38. Craig, E. M., J. Stricker, …, A. Mogilner. 2015. Model for adhesion clutch explains biphasic relationship between actin flow and traction at the cell leading edge. *Phys. Biol.* 12:035002.
39. Steucke, K. E., P. V. Tracy, …, P. W. Alford. 2015. Vascular smooth muscle cell functional contractility depends on extracellular mechanical properties. *J. Biomech.* 48:3044–3051.
40. Bangasser, B. L., S. S. Rosenfeld, and D. J. Odde. 2013. Determinants of maximal force transmission in a motor-clutch model of cell traction in a compliant microenvironment. *Biophys. J.* 105:581–592.
41. Elosegui-Artola, A., E. Bazellières, …, P. Roca-Cusachs. 2014. Rigidity sensing and adaptation through regulation of integrin types. *Nat. Mater.* 13:631–637.
42. Weinberg, S. H., D. B. Mair, and C. A. Lemmon. 2017. Mechanotransduction dynamics at the cell-matrix interface. *Biophys. J.* 112:1962–1974.
43. Gong, Z., S. E. Szczesny, …, V. B. Shenoy. 2018. Matching material and cellular timescales maximizes cell spreading on viscoelastic substrates. *Proc. Natl. Acad. Sci. USA.* 115:E2686–E2695.
44. Chaudhuri, O., L. Gu, …, D. J. Mooney. 2015. Substrate stress relaxation regulates cell spreading. *Nat. Commun.* 6:6364.
45. Maiuri, P., J.-F. Rupprecht, …, R. Voituriez. 2015. Actin flows mediate a universal coupling between cell speed and cell persistence. *Cell.* 161:374–386.
46. Wu, P.-H., A. Giri, …, D. Wirtz. 2014. Three-dimensional cell migration does not follow a random walk. *Proc. Natl. Acad. Sci. USA.* 111:3949–3954.
47. Gardner, M. K., B. D. Charlebois, …, D. J. Odde. 2011. Rapid microtubule self-assembly kinetics. *Cell.* 146:582–592.
48. Castle, B. T., D. J. Odde, and D. K. Wood. 2019. Rapid and inefficient kinetics of sickle hemoglobin fiber growth. *Sci. Adv.* 5:eaau1086.
49. Brooks, P. C., A. M. Montgomery, …, D. A. Cheresh. 1994. Integrin alpha v beta 3 antagonists promote tumor regression by inducing apoptosis of angiogenic blood vessels. *Cell.* 79:1157–1164.
50. Ivkovic, S., C. Beadle, …, S. S. Rosenfeld. 2012. Direct inhibition of myosin II effectively blocks glioma invasion in the presence of multiple motogens. *Mol. Biol. Cell.* 23:533–542.
51. Sakamoto, T., J. Limouze, …, J. R. Sellers. 2005. Blebbistatin, a myosin II inhibitor, is photoinactivated by blue light. *Biochemistry.* 44:584–588.
52. Raman, P. S., C. D. Paul, …, K. Konstantopoulos. 2013. Probing cell traction forces in confined microenvironments. *Lab Chip.* 13:4599–4607.
53. Coué, M., S. L. Brenner, …, E. D. Korn. 1987. Inhibition of actin polymerization by latrunculin A. *FEBS Lett.* 213:316–318.
54. Etienne-Manneville, S. 2013. Microtubules in cell migration. *Annu. Rev. Cell Dev. Biol.* 29:471–499.
55. Castle, B. T., S. McCubbin, …, D. J. Odde. 2017. Mechanisms of kinetic stabilization by the drugs paclitaxel and vinblastine. *Mol. Biol. Cell.* 28:1238–1257.
56. Harpold, H. L. P., E. C. Alvord, Jr., and K. R. Swanson. 2007. The evolution of mathematical modeling of glioma proliferation and invasion. *J. Neuropathol. Exp. Neurol.* 66:1–9.
57. Stricker, J., T. Falzone, and M. L. Gardel. 2010. Mechanics of the F-actin cytoskeleton. *J. Biomech.* 43:9–14.
58. Jülicher, F., K. Kruse, …, J. F. Joanny. 2007. Active behavior of the cytoskeleton. *Phys. Rep.* 449:3–28.
59. Moreau, H. D., C. Blanch-Mercader, …, A. M. Lennon-Duménil. 2019. Macropinocytosis overcomes directional bias in dendritic cells due to hydraulic resistance and facilitates space exploration. *Dev. Cell.* 49:171–188.e5.
60. Pathak, A., and S. Kumar. 2012. Independent regulation of tumor cell migration by matrix stiffness and confinement. *Proc. Natl. Acad. Sci. USA.* 109:10334–10339.
61. Azoitei, M. L., J. Noh, …, G. Danuser. 2019. Spatiotemporal dynamics of GEF-H1 activation controlled by microtubule- and Src-mediated pathways. *J. Cell Biol.* 218:3077–3097.
62. Klank, R. L., S. S. Rosenfeld, and D. J. Odde. 2018. A Brownian dynamics tumor progression simulator with application to glioblastoma. *Converg. Sci. Phys. Oncol.* 4:015001.
63. Rockne, R., E. C. Alvord, Jr., …, K. R. Swanson. 2009. A mathematical model for brain tumor response to radiation therapy. *J. Math. Biol.* 58:561–578.
64. Bell, G. I. 1978. Models for the specific adhesion of cells to cells. *Science.* 200:618–627.
65. Tobacman, L. S., and E. D. Korn. 1983. The kinetics of actin nucleation and polymerization. *J. Biol. Chem.* 258:3207–3214.
66. Gillespie, D. T. 1977. Exact stochastic simulation of coupled chemical reactions. *J. Phys. Chem.* 81:2340–2361.
67. Seetapun, D., B. T. Castle, …, D. J. Odde. 2012. Estimating the microtubule GTP cap size in vivo. *Curr. Biol.* 22:1681–1687.
68. Prahl, L. S., B. T. Castle, …, D. J. Odde. 2014. Quantitative analysis of microtubule self-assembly kinetics and tip structure. *Methods Enzymol.* 540:35–52.
69. Molloy, J. E., J. E. Burns, …, D. C. White. 1995. Movement and force produced by a single myosin head. *Nature.* 378:209–212.
70. Jiang, G., G. Giannone, …, M. P. Sheetz. 2003. Two-piconewton slip bond between fibronectin and the cytoskeleton depends on talin. *Nature.* 424:334–337.
71. Bangasser, B. L., and D. J. Odde. 2013. Master equation-based analysis of a motor-clutch model for cell traction force. *Cell. Mol. Bioeng.* 6:449–459.
72. Lele, T. P., C. K. Thodeti, …, D. E. Ingber. 2008. Investigating complexity of protein-protein interactions in focal adhesions. *Biochem. Biophys. Res. Commun.* 369:929–934.
73. Schafer, D. A., P. B. Jennings, and J. A. Cooper. 1996. Dynamics of capping protein and actin assembly in vitro: uncapping barbed ends by polyphosphoinositides. *J. Cell Biol.* 135:169–179.

Biophysical Journal, Volume *118*

Supplemental Information

Predicting Confined 1D Cell Migration from Parameters Calibrated to a 2D Motor-Clutch Model

Louis S. Prahl, Maria R. Stanslaski, Pablo Vargas, Matthieu Piel, and David J. Odde

Supporting Methods

1D cell migration simulator

Previous implementations of the cell migration simulator (CMS) either considered a cell migrating on a 2D unconfined surface with varying adhesiveness or mechanics (1–3), or explicitly modeled the underlying fiber network geometry (4). In the present study, we modified the CMS to model cell migration in a single spatial dimension, hereafter referred to as the 1D CMS (schematic is shown in **Figure 1A**). As with previous versions, the 1D CMS contains a number of protrusion modules (j represents the module number), each functioning as an instance of the motor-clutch model (5). Modules contain $n_{clutch,j}$ adhesion clutches, each of which is modeled as an elastic spring with stiffness κ_{clutch}. Clutches bind to a compliant substrate (also an elastic spring with stiffness κ_{sub}) at a reference point $x_{ref,j}$. Clutch binding occurs at a rate k_{on}, which is assumed to be a first-order reaction. Unbinding of the ith clutch in a particular ensemble occurs at a rate $k_{off,i}$ that scales with force by a single exponential (6), also known as a slip bond.

$$k_{off,i} = k_{off} exp\left(\frac{F_{clutch,i}}{F_{bond}}\right) \qquad \textbf{Eqn. S1}$$

Here, k_{off} is the unloaded unbinding rate, F_{bond} is the characteristic bond force, and $F_{clutch,i}$ is the force on the ith clutch. Modules also contain $n_{motor,j}$ myosin II motors, each of which is capable of generating F_{motor} stall force. Motors slide an F-actin bundle to generate retrograde flow (v_{flow}), which extends clutches that are bound to the substrate. As forces build on the substrate through bound clutches, F-actin flow slows from the myosin II motor unloaded velocity v_{motor} by a linear force-velocity relationship (5).

$$v_{flow} = v_{motor}\left(1 - \frac{\kappa_{sub}\Delta x_{sub,j}}{F_{motor} n_{motor,j}}\right) \qquad \textbf{Eqn. S2}$$

We note that individual actin filaments polymerizing against a tensed plasma membrane are also capable of generating F-actin retrograde flow and traction forces, so each filament can be regarded as a motor capable of generating pN-scale forces and velocities of ~100 nm s^{-1} in the absence of load. In addition, polymerizing actin filaments also obey a monotonic force-velocity relationship that can be approximated by **Eqn. S2**. Substrate spring displacement on the jth module is $\Delta x_{sub,j}$. Each module contains a rigid bar of F-actin with length $A_{F,j}$. Modules extend by actin polymerization (v_{actin}) from a soluble pool of G-actin subunits, A_G. Actin polymerization (v_{actin}) is defined by a maximum actin polymerization velocity $v_{actin,max}$, A_G, and the total available actin length in the cell, A_{total}.

$$v_{actin} = v_{actin,max}\left(\frac{A_G}{A_{total}}\right) \qquad \textbf{Eqn. S3}$$

A mass balance constrains A_{total} and A_G in a simulated cell with n_{module} modules.

$$A_{total} = A_G + \sum_{j=1}^{n_{module}} A_{F,j} \qquad \textbf{Eqn. S4}$$

F-actin is depolymerized when it passes the motor position within modules ($x_{motor,j}$) and returns to the G-actin pool in the cell. Module clutches also unbind when their bound position ($x_{clutch,i}$) extends past $x_{motor,j}$. Modules contain a compliant cell spring with stiffness κ_{cell} that represents the nucleo-cytoskeletal compliance of the cell and connects the module motors to the central cell body. A force balance applies to each module spring, clutches, and cell spring.

$$F_{module,j} = \kappa_{cell}\Delta x_{cell,j} = \kappa_{clutch}\sum_{i=1}^{n_{clutch,j}}\Delta x_{clutch,i} = \kappa_{sub}\Delta x_{sub,j} \qquad \textbf{Eqn. S5}$$

Within the cell body, an ensemble of $n_{clutch,cell}$ clutches transmit force to the substrate under the cell body in the same way as module clutches. F_{cell} represents the force from extension of the cell substrate spring $\Delta x_{sub,cell}$

$$F_{cell} = \kappa_{clutch}\sum_{i=1}^{n_{clutch,j}}\Delta x_{clutch,i} = \kappa_{sub}\Delta x_{sub,cell} \qquad \textbf{Eqn. S6}$$

Cell body clutches are not subject to direct myosin II forces from F-actin flow but extend as the cell position x_{cell} is updated by force balance on modules and the cell body.

$$F_{cell} + \sum_{j=1}^{n_{module}} F_{module,j} = 0 \qquad \textbf{Eqn. S7}$$

Module capping occurs at a first-order rate k_{cap} and capped modules no longer extend by actin polymerization. Nucleation of new modules occurs at a rate k_{nuc}, and k_{nuc} is a function of the basal nucleation rate ($k_{nuc,0}$) and the available G-actin pool raised to the fourth power (7).

$$k_{nuc} = k_{nuc,0}\left(\frac{A_G}{A_{total}}\right)^4 \qquad \textbf{Eqn. S8}$$

New modules have initial length L_{cell} and are assigned motors and clutches as a fraction of the available pool of each within the cell (1). Modules that shorten past a minimum length L_{min} are eliminated and their contents (motors, clutches, F-actin) are returned to the common cellular pool.

In the previous CMS, modules are randomly nucleated at a uniformly distributed angle θ within the 2D plane. Module nucleation in 1D can only occur in the +x or –x direction, with the probability of a new module being generated in the +x direction defined by a cytoskeletal polarity factor ψ_{pol}. The complementary probability 1-ψ_{pol} is the probability that a new module is generated in the –x direction. The case where ψ_{pol} = 0.5 represents an equal likelihood of module generation in the +x and –x directions, which can be thought of as a "non-polarized" cell. In a cell where ψ_{pol} < 0.5, the polarity would bias module nucleation in the –x direction. Essentially, the probability of module nucleation in the +x direction follows a Bernoulli distribution with parameter ψ_{pol}. All simulation parameter values are reported in **Table S1** and changes for specific conditions are described in the figure legends.

Simulation implementation and analysis

Event selection was determined using kinetic Monte Carlo (8) using a unique, randomly generated seed for each run. The order of events in each step of the model algorithm is previously described (1). Simulation initial conditions included two modules oriented in the +x direction and one module oriented in the –x direction (each module of length L_{cell}) and all clutches unbound. Individual runs represent data collected over 5-7 hours of simulated time. Simulations were run in Matlab (The MathWorks Inc., Natick, MA) on in-house computing cores or using high performance computing cores in the Minnesota Supercomputing Institute.

To quantify simulated cell migration, cell body position (x_{cell}) was sampled every 5 minutes of simulated time. We excluded the first hour of simulated data from analysis and subsequent data were filtered to remove large displacements in cell position that result in velocities >1 μm s^{-1} (1). MSD was calculated from cell body position using the overlap method (1, 9) and fit to cell motility models in order to extract parameters related to cell migration (described in the main text) using a custom Matlab analysis script. For motility model fitting, we used the first half of MSD versus time lag data for each individual cell (1).

Cell culture and transfection

Human U251 glioma cells were cultured in DMEM/F-12 (Gibco, ThermoFisher Scientific, Waltham, MA) supplemented with 10% (v/v) fetal bovine serum (Gibco) and 100 μg mL^{-1} penicillin/streptomycin antibiotic (Corning, Inc., Corning, NY), maintained in a humid incubator at 37°C and 5% CO_2, and passaged using 0.25% trypsin-EDTA (Corning). Transfection of fluorescent proteins was performed using FuGENE® HD transfection reagent (Promega Corporation, Madison, WI) at least 24 hours prior to seeding cells in devices, as described previously (3). Fluorescent fusion plasmids included eGFP-β-actin (gift from Paul Letourneau, University of Minnesota) and EB1-eGFP (gift from Lynne Cassimeris, Lehigh University; (10)).

Pharmacological agents

Microtubule-targeting agents vinblastine sulfate (Sigma-Aldrich Corp., St. Louis, MO) and paclitaxel (Sigma) were stored in stock concentrations of 1 mM in DMSO, Y-27632 dihydrochloride (Sigma) was stored as 15 mM stock in water, cyclo-(Arg-Gly-Asp) peptide (cRGD; Enzo Life Sciences Inc., Farmingdale, NY) was stored at 4 mM in PBS, and latrunculin A (Sigma) was stored at 0.5 mM in DMSO. All drug stocks were kept frozen at -20°C. DMSO volume added to dishes did not exceed 0.1% in any condition.

Microchannel device design and assembly

Devices feature three rectangular seeding ports (shown in **Figure 2A**) with 15 µm diameter pillars evenly spaced at 25 µm intervals and with opposite rows of channels aligned towards the device exterior. Entrances to channels are 100 µm long funnels that taper from an initial width of 18 µm to a final channel width of 12 µm. Chromium-plated quartz photomasks were generated by the Minnesota Nano Center (MNC) facilities. Device molds were manufactured on 4” silicon wafers using photolithography with epoxy-based SU-8 2005 negative photoresist (MicroChem Corp., Wesborough, MA).

Briefly, a 5 µm thick photoresist layer was spin coated on wafers using a CEE 100 spin coater (Brewer Science, Rolla, MO), followed by a 2 minute soft bake at 95°C. Wafer surfaces were exposed to UV radiation at 12 mJ s cm^{-2} for 10 seconds using a MA6 mask aligner (Karl Süss MicroTec, Garching, Germany). Following a 3-minute post-exposure bake at 95°C, excess photoresist was removed using SU-8 developer (MicroChem). After curing, the height of individual photoresist structures was verified using a P-16 stylus profilometer (KLA-Tencor, Milpitas, CA). To minimize PDMS adhesion and facilitate device removal, wafers were treated with trichloro(1H,1H,2H,2H-perfluorooctyl)silane (Sigma, St. Louis, MO) under vacuum before casting.

Sylgard® 184 PDMS elastomer (Dow Corning, Midland, MI) was mixed in a 10:1 (base:curing agent) ratio, poured on wafers or epoxy replicas and allowed to cure at 75°C for 2 hours. Devices were cut and peeled from molds, seeding ports were added using a 3 mm radius circular punch (Ted Pella Inc., Redding, CA), and devices were manually resized to a 1.5 x 1.0 cm footprint centered around the seeding ports using a razor blade. Cut and resized devices were cleaned with adhesive tape and 70% ethanol in a sonic bath for 1 minute and then allowed to dry under a stream of compressed air. Devices and 35 mm glass bottom dishes (20 mm No. 0 coverglass, MatTek Corp., Waltham, MA) were activated with a plasma cleaner (PDC-32 G, Harrick, Ithaca, NY) for 30 seconds, then bonded at 75°C for 1 hour.

Assembled devices were plasma treated for 1 minute and a solution of 10 μg mL^{-1} bovine plasma fibronectin (Sigma) in water was directly added to seeding ports. After 1 hour, devices were washed with PBS and stored overnight at 4°C before seeding cells. Devices were pre-incubated in media (containing drugs or DMSO, if appropriate) at 37°C for at least 1 hour prior to cell seeding. Cells were detached from flasks using 0.25% trypsin-EDTA (Corning), re-suspended in culture media, and nuclei were labeled using a nucleus counterstain (NucBlue Live, ThermoFisher) according to manufacturer instructions. Following labeling, cells were re-suspended in media to a density of $20x10^6$ cells per mL. Media was removed from device dishes and seeding ports, and a 5 μL aliquot of cell suspension (10^5 cells) was added to each seeding port. Devices containing cells were returned to the incubator for 30 minutes before adding sufficient media to cover the device inlets.

Time-lapse imaging and image processing

Devices were pre-incubated in media (containing drugs or DMSO) at 37°C for at least 1 hour prior to cell seeding. Cells were detached from flasks using 0.25% trypsin-EDTA (Corning) and re-suspended in culture media. Nuclei were labeled with NucBlue Live counterstain (ThermoFisher) according to manufacturer instructions. After centrifuging and re-suspending cells to a density of $20x10^6$ cells per mL, a 5 μL aliquot (10^5 cells) was added to each seeding port. Devices containing cells were returned to the incubator for 30 minutes before adding sufficient drug-containing media (~2 mL) to cover the entire device.

Time-lapse images were acquired on either a Nikon TiE or Nikon Ti2 epifluorescence microscope under control of NIS Elements software (Nikon Instruments Inc., Melville, NY) a ProScan III motorized stage (Prior Scientific Inc., Rockland, MA), a white light transmitted LED (CoolLED Ltd., Andover, UK), LED fluorescence illumination (Spectra X; Lumencor, Beaverton, OR), and DAPI/FITC/TxRed filter set (Chroma, Cat#89014), and a Zyla 4.2 or 5.5 sCMOS camera (Andor Technologies Ltd., Belfast, UK). Stable environmental control (5% CO_2 at 37°C) was provided using a stagetop incubator (OkoLab Llc., Ottaviano, Italy). Time-lapse images of cells in channels were acquired in the transmitted and DAPI channels every 5 minutes over 8-18 hours using a 20x/0.45NA phase I lens with 2x2 pixel binning (645 nm per pixel).

Images for analyzing cell length and nucleus position were acquired using a 40x/0.95NA phase II lens with no pixel binning (163 nm per pixel) in the FITC and DAPI channels. Cell length and nucleus position were measured manually using Fiji (11). First, cell images were oriented, so the leading and trailing edges were on the right and left of the image panel, respectively. Direction of motion was ascertained from a short time-lapse acquisition. Next, we subtracted background, and

a rectangular box was manually drawn around the cell boundaries. Cell and nucleus coordinates were measured from the center of mass using the measurement tool in Fiji. Normalized cell coordinates were defined as 0 being the cell rear (leftmost protrusion) and 1 being the cell front (rightmost protrusion), and normalized nucleus position was calculated as the ratio of the distance between the cell rear and nucleus centroid and the total length of the cell. Movies for microtubule growth rate were obtained from the FITC channel through a 100x/1.49 NA lens using streaming acquisition at 300 milliseconds per frame. Microtubule tip position for individual EB1-eGFP decorated plus-ends were measured with sub-pixel accuracy using TipTracker_v3 without modification (12). Individual microtubule growth velocities were calculated by dividing the mean length increment change by the acquisition time (0.3 s) as previously described (3).

Confocal z-stacks

Confocal z-stacks were acquired on an LSM7 LIVE swept-field confocal microscope (Carl Zeiss Microscopy, LLC; Jena, Germany) using a 40x/0.95 NA objective. The 488 nm and 405 nm lasers and BP 520-555 and BP 415-480 filters enabled simultaneous acquisition of the blue and green channels. Images were digitized using a 512-pixel 11-bit linear detector. Zen software (Zeiss) controlled the microscope system during imaging.

Semi-automated nucleus tracking

Nucleus position coordinates were obtained from the DAPI channel of image sequences using custom semi-automated image segmentation and tracking Matlab scripts (MathWorks) as previously described for tracking cells expressing fluorescent labels (2). Image sequences were cropped to include a single cell that was not in contact with another cell, as ascertained from phase contrast images. Skipped frames (e.g. due to contact with another cell or object in the channel) were omitted during analysis, as described previously (1). MSD were calculated from cell coordinates using the overlap method (9) and cell trajectories were analyzed using the same motility models as simulated cell trajectories.

Statistical analysis

Experimental data represent accumulated numbers of cells from $N \geq 2$ independent experiments for each condition. Numbers of simulations are reported in the figure legends; simulations start from a randomly generated seed and each simulation is considered independent for the calculation of n. For comparisons, a non-parametric Kruskal-Wallis one-way analysis of variance (ANOVA) was used since data did not typically follow a normal distribution. The Dunn-Sidăk test was employed to correct for multiple comparisons. Statistical significance for a particular comparison was reached when $p < 0.01$. All statistical analysis of experimental or simulated data was performed using the Matlab Statistics Toolbox (MathWorks).

Supplemental Notes

Mechanical properties and elastic behavior of F-actin, clutches and the cell spring

As with previous iterations of the motor-clutch model (5), F-actin is assumed to be rigid and inextensible. The cell spring (κ_{cell}) is connected in series with the F-actin bar, clutches, and substrate, representing the nucleo-cytoskeletal compliance of the cell. The chosen cell spring constant value ($\kappa_{cell} = 10^4$ pN nm^{-1}; **Table S1**) was used in previous studies employing the CMS (1), and is consistent with the rigid modulus measured for isolated actin filaments and stress fibers (E ~ 10^4 kPa) (13).

We assumed elastic behavior of the cell spring, since simulations generate ~10-100 pN forces (based on the total number of myosin II motors allocated to a module), which result in small (<1 nm) deformations. Larger forces that could significantly deform the cell may induce non-linear (strain stiffening) or visco-elastic behaviors of F-actin networks (13). Modeling these would require more detailed theoretical treatment of the F-actin properties, but in the present study we did not observe any cell deformations that could not be explained due to actin-based extension and retraction of cell protrusions. The microfluidic devices used in the present study are assembled from rigid PDMS (E = 10^3 kPa as measured in (14)) and glass (E ~ 10^6 kPa) and thus are not significantly deformed by cells, so we assumed an elastic spring best captured their behavior. Clutches are similarly treated as elastic springs, since individual clutch binding and unbinding occurs on the milliseconds timescale, allowing us to ignore short viscous relaxation times (5).

Estimation of hydrodynamic drag within channels

Some cells are reportedly able to use osmotic pressure-based motility to drive actin-independent confined migration (15). This enables them to migrate even through micrometer-scale pores that limit protrusion by F-actin, and where hydrostatic pressure would typically preclude movement. In the present study, we report that U251 cells are sensitive to actin polymerization inhibition by latrunculin A (**Figure 6**) causing us to conclude that these cells do not use an osmotic pressure-based migration mechanism. Nevertheless, we sought to determine the magnitude of hydrodynamic drag in U251 cells to evaluate whether it should be included as a force in the model.

Li and Sun (16) estimate the hydrodynamic drag coefficient (d_g) for a cell within a square channel with dimension b and length l, filled with a medium of viscosity μ.

$$d_g = \frac{12\mu L}{b^2} \qquad \textbf{Eqn. S9}$$

The channels in our devices have a horizontal width of 12 μm and 5 μm height, yielding a 60 μm^2 cross-sectional area. Approximating the channel cross section as a 7.5 μm square, and estimating 1 mm total channel length, and medium viscosity equal to water ($\mu = 10^{-3}$ Pa s), **Eqn. S9** yields $d_g = 0.21$ pN s μm^{-3} for hydrodynamic drag coefficient.

Hydrodynamic force can be calculated for a cell moving with velocity v_{cell} through a channel with cross sectional area A.

$$F_{drag} = -d_g A v_{cell}$$ **Eqn. S10**

Using the value of d_g calculated by **Eqn. S9**, the mean velocity obtained from experiments ($v_{cell} = 0.51\ \mu m\ s^{-1}$; **Figure 3B**) and assuming the same cross-sectional area as before, **Eqn. S10** produces a drag force $F_{drag} = -6.5$ pN. This value is of similar magnitude to the force generated by ~4 myosin II motors, and much smaller than cellular traction force estimates (17), so we consider hydrodynamic drag as negligible in our experimental system.

Supporting Tables

Table S1. 1D CMS base parameter set.

Parameter	Description	Value	Source
Motor parameters			
n_{motor}	Number of myosin II motors per cell	10^3	Adjustable, ~10^2-10^5 required for nN total traction force (17)
$n_{motor,module}$	Maximum number of myosin II motors per module	100	Adjustable, constrained to 0.1*n_{motor} (1)
F_{motor}	Stall force for a single myosin II motor	2 pN	Measured *in vitro* by optical tweezers (18)
v_{motor}	Maximum (unloaded) myosin II sliding velocity on F-actin	120 nm s^{-1}	Measured in (1); ~100 nm s^{-1} observed in other cells (5, 19)
Clutch parameters			
n_{clutch}	Number of clutch bonds (e.g. integrin-mediated adhesion complexes) per cell	750	Adjustable, $n_{clutch} \approx n_{motor}$ by theoretical estimate (20)
$n_{clutch,module}$	Maximum number of clutch bonds per module	75	Adjustable, 0.1*n_{clutch} (1)
F_{bond}	Characteristic slip bond force for clutches	2 pN	Measured *in vitro* by optical tweezers (21)
κ_{clutch}	Spring constant of the clutch spring	0.8 pN nm^{-1}	Theoretical estimate, on the order of 1 pN nm^{-1} (20)
k_{on}	Pseudo-first order association rate between clutches and F-actin	1 s^{-1}	Theoretical estimate, 10*k_{off} (22)
k_{off}	Basal (unloaded) first-order clutch dissociation rate from F-actin	10^{-1} s^{-1}	Minimum unbinding rate measured by photobleaching (23)
Substrate parameters			
$\kappa_{csubstrate}$	Substrate spring stiffness	10^3 pN nm^{-1}	Estimated PDMS modulus (14)
Cell body and actin parameters			
k_{cap}	Module capping rate	10^{-3} s^{-1}	Experimental estimate (1, 24)
$k_{nuc,0}$	Maximum module nucleation rate	1 s^{-1}	Experimental estimate (1, 3)
$v_{actin,max}$	Maximum actin polymerization velocity	200 nm s^{-1}	Experimental estimate (3, 5)
A_{total}	Total actin pool available for protrusions	100 µm	Estimate of total cell protrusion length (1)
κ_{cell}	Spring constant of the cell spring	10^4 pN nm^{-1}	Adjustable, estimated in (1)
L_{cell}	Initial module F-actin length	5 µm	Simulation initial condition
L_{min}	Minimum module F-actin length	100 nm	Adjustable
$n_{clutch,cell}$	Number of cell body clutches	10	Adjustable, estimated in (1)
ψ_{pol}	Cell polarity factor	0.9	This study

Table S2. Pairwise p-values for the experimental and simulation conditions involving inhibitors of integrin clutches and myosin II activation. Related to **Figure 4** and **Figure 5**.

Figure 6A. 1D CMS motility (n_{motor} = 1000, n_{clutch} varies; ψ_{pol} = 0.9)

n_{clutch}	25	75	250	750
8	0.95	0.02	1	0.06
25		0.01	0.98	0.63
75			0.80	10^{-4}
250				0.21

Figure 6B. 1D CMS velocity (n_{motor} = 1000; n_{clutch} varies; ψ_{pol} = 0.9)

n_{clutch}	25	75	250	750
8	0.05	10^{-8}	0.63	0.56
25		0.37	0.99	0.007
75			0.39	10^{-5}
250				0.09

Figure 6C. cRGD experiment motility

[cRGD]	0.1	0.3	1
0	0.54	0.97	0.67
0.1		0.99	0.09
0.3			0.41
1			

Figure 6D. cRGD experiment velocity

[cRGD]	0.1	0.3	1
0	0.0012	0.00088	10^{-14}
0.1		0.99	$<10^{-15}$
0.3			$<10^{-15}$
1			

Figure 6E. 1D CMS motility (n_{motor} = 1000; n_{clutch} = 750; ψ_{pol} = 0.9)

n_{motor}	500	750
100	0.99	10^{-4}
500		0.0028
750		

Figure 6F. 1D CMS velocity (n_{motor} = 1000; n_{clutch} = 750; ψ_{pol} = 0.9)

n_{motor}	500	750
100	0.099	0.0079
500		0.88
750		

Supporting Figures

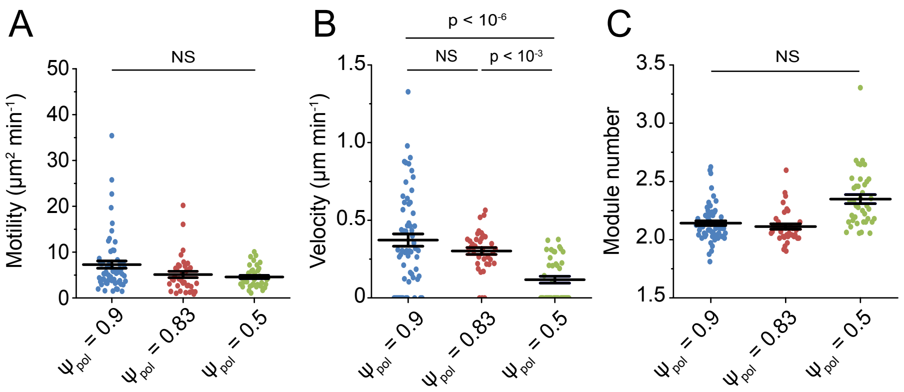

Figure S1. Simulated motility coefficient, velocity, and module number for a 1D cell migration simulator with variable polarity. Related to **Figure 1**.

Motility coefficients (**left**), velocities (**center**), and module number (**right**) for the simulations in **Figure 1** where ψ_{pol} = 0.5, 0.83, or 0.9, n = 60, 36, 40 simulations. Individual motility coefficients and velocities were obtained from fits to **Eqn. 2** in the **Main Text**. Error bars represent mean ± SEM. Pairwise statistics were computed by a Kruskal-Wallis one-way ANOVA with Dunn-Sidăk correction for multiple comparisons, NS = no significant difference between groups, $p > 0.01$. All simulations were run with n_{motor} = 1000 and n_{clutch} = 750, all other parameter values reported in **Table S1**.

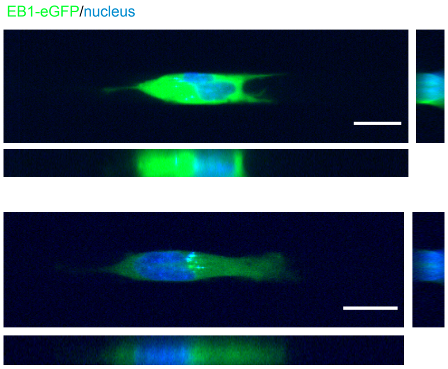

Figure S2. Confocal image stacks of cells in microchannels. Related to **Figure 2**.

A. Example confocal z-stacks of U251 cells expressing EB1-eGFP (green) and nucleus stain (blue). Images were acquired at 40x magnification. Images were oriented such that the channel inlets are to the left, while the outlets are to the right and adjusted for brightness/contrast in both green and blue channels. A view of the x-y plane is shown left, and an x-y slice through the center is shown at right. Total z-stack height, 11.43 μm; horizontal scale, 20 μm.

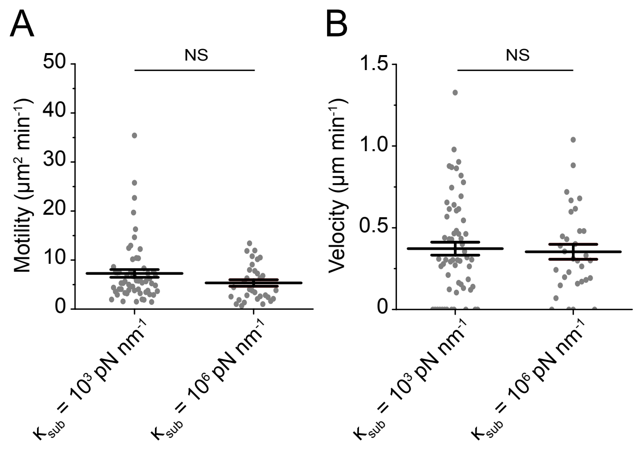

Figure S3. Effects of varying substrate spring constant on simulated cell motility coefficient and speed. Related to **Figure 1**.

Motility coefficients (**left**) and velocities (**right**) for simulations where $\kappa_{sub} = 10^3$ pN nm^{-1} or $\kappa_{sub} = 10^6$ pN nm^{-1}, n = 60, 24 simulations. Individual motility coefficients and velocities were obtained from fits to **Eqn. 2** in the **Main Text**. Error bars represent mean ± SEM. Pairwise statistics were computed by the Mann-Whitney U test, NS = no significant difference between groups, $p > 0.01$. All simulations were run with $n_{motor} = 1000$, $n_{clutch} = 750$, and $\psi_{pol} = 0.9$, all other parameter values reported in **Table S1**.

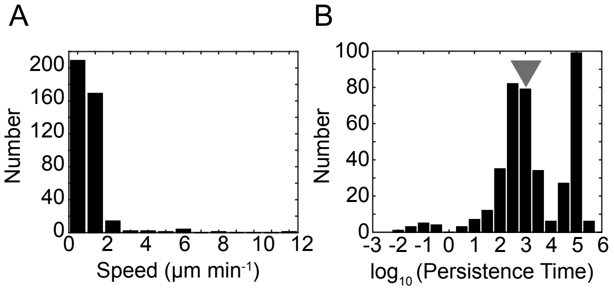

Figure S4. Persistent random walk fits predict persistence times that are longer than the experimental imaging duration. Related to **Figure 2** and **Figure 3.**

A. Speeds obtained from fits to a persistent random walk model (**Eqn. 1** in the **Main Text**), n = 403 cells from 12 independent experiments. S = 0.74 ± 0.05 μm min^{-1} or S = 12.3 ± 0.8 nm s^{-1} (mean ± SEM).

B. Persistence time (log_{10}-transformed) for the data in panel **A**. Arrow indicates the maximum experimental imaging duration of 18 hours or 1080 minutes ($log_{10}(1080) = 3.03$). Individual cell trajectories yielded a mean log_{10}-transformed persistence time of 3.1 ± 0.25 (mean ± SEM), corresponding to 1258 minutes.

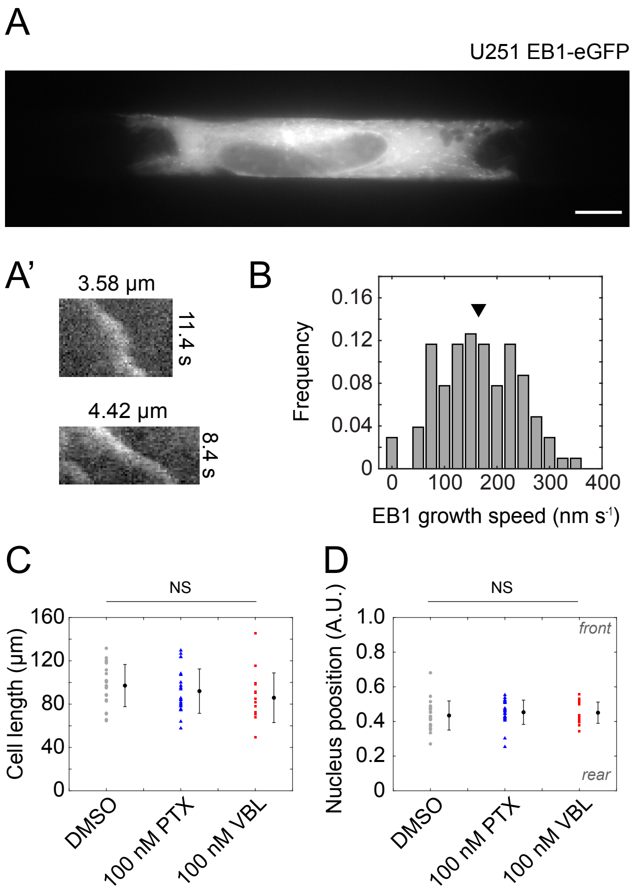

Figure S5. Measurements of microtubule dynamics as related to cell length and nucleus position in channels. Related to **Figure 7**.

A. Example image of a U251 cell in a microchannel device expressing EB1-eGFP. Image was captured at 100x magnification. Scale bar, 10 μm. **A'**. Example kymographs showing microtubule plus-end dynamics in U251 cells within channels. Microtubule growth speeds were measured for n = 103 microtubules in N = 5 cells using TipTracker software (12).

B. Histogram of microtubule growth speeds measured in U251 cells expressing EB1-eGFP. Black arrow marks a mean microtubule growth speed of 165 ± 7.5 nm s^{-1} (± S.E.M.).

C. Lengths of cells expressing EB1-eGFP and stained with NucBlue nucleus dye and treated with vehicle (DMSO), 100 nM PTX, or 100 nM VBL. Data points represent n = 21, 22, 15 measurements collected from 3 independent experiments.

D. Nucleus position for the cells in panel **C**. Cell lengths are normalized so that 0 marks the cell rear (trailing protrusion) and 1 denotes the cell front (leading protrusion). Error bars in panels **C** and **D** are mean ± standard deviation. Kruskal-Wallis one-way ANOVA revealed no significant differences (NS) between groups in panels **C** and **D**.

Supporting Movies

Movie S1. U251 glioma cells with fluorescently labeled nuclei migrating in microchannel devices.

Time-lapse images were collected every 5 minutes at 20x magnification with 2x2 binning (645 nm spatial sampling). Images were acquired in both the transmitted channel using phase contrast optics and LED fluorescence excitation (395 nm) using a DAPI/FITC/TxRed filter set. Scale bar, 50 μm.

Movie S2. U251 glioma cells expressing EGFP-actin and treated with vehicle control or latrunculin A migrating in microchannel devices.

Time-lapse images were collected every 5 minutes at 20x magnification with 2x2 binning (645 nm spatial sampling). Images were acquired in both the transmitted channel using phase contrast optics and using LED fluorescence excitation (395 nm and 470 nm) through a DAPI/FITC/TxRed filter set. Conditions include DMSO vehicle (top), 50 nM latrunculin A (middle), and 500 nM latrunculin A (bottom). Scale bar, 50 μm.

Supporting References

1. Bangasser, B.L., G.A. Shamsan, C.E. Chan, K.N. Opoku, E. Tüzel, B.W. Schlichtmann, J.A. Kasim, B.J. Fuller, B.R. McCullough, S.S. Rosenfeld, and D.J. Odde. 2017. Shifting the optimal stiffness for cell migration. Nat. Commun. 8: 1–10.
2. Klank, R.L., S.A. Decker Grunke, B.L. Bangasser, C.L. Forster, M.A. Price, T.J. Odde, K.S. SantaCruz, S.S. Rosenfeld, P. Canoll, E.A. Turley, J.B. McCarthy, J.R. Ohlfest, and D.J. Odde. 2017. Biphasic Dependence of Glioma Survival and Cell Migration on CD44 Expression Level. Cell Rep. 18: 23–31.
3. Prahl, L.S., P.F. Bangasser, L.E. Stopfer, M. Hemmat, F.M. White, S.S. Rosenfeld, and D.J. Odde. 2018. Microtubule-Based Control of Motor-Clutch System Mechanics in Glioma Cell Migration. Cell Rep. 25: 2591-2604.e8.
4. Estabridis, H.M., A. Jana, A. Nain, and D.J. Odde. 2018. Cell Migration in 1D and 2D Nanofiber Microenvironments. Ann. Biomed. Eng. 46: 392–403.
5. Chan, C.E., and D.J. Odde. 2008. Traction dynamics of filopodia on compliant substrates. Science (80-.). 322: 1687–1691.
6. Bell, G.I. 1978. Models for the specific adhesion of cells to cells. Science. 200: 618–27.
7. Tobacman, L.S., and E.D. Korn. 1983. The kinetics of actin nucleation and polymerization. J. Biol. Chem. . 258: 3207–3214.
8. Gillespie, D.T. 1977. Exact stochastic simulation of coupled chemical reactions. J. Phys. Chem. 81: 2340–2361.
9. Dickinson, R.B., and R.T. Tranquillo. 1993. Optimal estimation of cell movement indices

from the statistical analysis of cell tracking data. AIChE J. 39: 1995–2010.

10. Seetapun, D., B.T. Castle, A.J. McIntyre, P.T. Tran, and D.J. Odde. 2012. Estimating the microtubule GTP cap size in vivo. Curr. Biol. 22: 1681–1687.

11. Schindelin, J., I. Arganda-Carreras, E. Frise, V. Kaynig, M. Longair, T. Pietzsch, S. Preibisch, C. Rueden, S. Saalfeld, B. Schmid, J.-Y. Tinevez, D.J. White, V. Hartenstein, K. Eliceiri, P. Tomancak, and A. Cardona. 2012. Fiji: an open-source platform for biological-image analysis. Nat. Methods. 9: 676–82.

12. Prahl, L.S., B.T. Castle, M.K. Gardner, and D.J. Odde. 2014. Quantitative analysis of microtubule self-assembly kinetics and tip structure. Methods Enzymol. 540: 35–52.

13. Stricker, J., T. Falzone, and M.L. Gardel. 2010. Mechanics of the F-actin cytoskeleton. J. Biomech. 43: 9–14.

14. Steucke, K.E., P. V Tracy, E.S. Hald, J.L. Hall, and P.W. Alford. 2015. Vascular smooth muscle cell functional contractility depends on extracellular mechanical properties. J. Biomech. 48: 3044–3051.

15. Stroka, K.M., H. Jiang, S.-H.H. Chen, Z. Tong, D. Wirtz, S.X. Sun, and K. Konstantopoulos. 2014. Water permeation drives tumor cell migration in confined microenvironments. Cell. 157: 611–623.

16. Li, Y., and S.X. Sun. 2018. Transition from actin-driven to water-driven cell migration depends on external hydraulic resistance. Biophys. J. 114: 2965–2973.

17. Mekhdjian, A.H., F. Kai, M.G. Rubashkin, L.S. Prahl, L.M. Przybyla, A.L. McGregor, E.S. Bell, J.M. Barnes, C.C. DuFort, G. Ou, A.C. Chang, L. Cassereau, S.J. Tan, M.W. Pickup, J.N. Lakins, X. Ye, M.W. Davidson, J. Lammerding, D.J. Odde, A.R. Dunn, and V.M. Weaver. 2017. Integrin-mediated traction force enhances paxillin molecular associations and adhesion dynamics that increase the invasiveness of tumor cells into a three-dimensional extracellular matrix. Mol. Biol. Cell. 28: 1467–1488.

18. Molloy, J.E., J.E. Burns, B. Kendrick-Jones, R.T. Tregear, and D.C.S. White. 1995. Movement and force produced by a single myosin head. Nature. 378: 209–212.

19. Elosegui-Artola, A., R. Oria, Y. Chen, A. Kosmalska, C. Pérez-González, N. Castro, C. Zhu, X. Trepat, and P. Roca-Cusachs. 2016. Mechanical regulation of a molecular clutch defines force transmission and transduction in response to matrix rigidity. Nat. Cell Biol. 18: 540–548.

20. Bangasser, B.L., S.S. Rosenfeld, and D.J. Odde. 2013. Determinants of maximal force transmission in a motor-clutch model of cell traction in a compliant microenvironment. Biophys. J. 105: 581–592.

21. Jiang, G., G. Giannone, D.R. Critchley, E. Fukumoto, and M.P. Sheet. 2003. Two-piconewton slip bond between fibronectin and the cytoskeleton depends on talin. Nature. 424: 334–337.

22. Bangasser, B.L., and D.J. Odde. 2013. Master equation-based analysis of a motor-clutch model for cell traction force. Cell. Mol. Bioeng. 6: 449–459.
23. Lele, T.P., C.K. Thodeti, J. Pendse, and D.E. Ingber. 2008. Investigating complexity of protein-protein interactions in focal adhesions. Biochem. Biophys. Res. Commun. 369: 929–934.
24. Schafer, D.A., P.B. Jennings, and J.A. Cooper. 1996. Dynamics of capping protein and actin assembly in vitro: Uncapping barbed ends by polyphosphoinositides. J. Cell Biol. 135: 169–179.
